# Metatranscriptomics reveals temperature-driven functional changes in microbiome impacting cheese maturation rate

**DOI:** 10.1038/srep21871

**Published:** 2016-02-25

**Authors:** Francesca De Filippis, Alessandro Genovese, Pasquale Ferranti, Jack A. Gilbert, Danilo Ercolini

**Affiliations:** 1 Department of Agricultural Sciences, University of Naples Federico II, Via Università 100, 80055 Portici, Italy; 2 Biosciences Division (BIO), Argonne National Laboratory, Argonne, IL, USA; 3 Departmen of Ecology & Evolution, University of Chicago, Chicago, IL, USA; 4 Department of Surgery, University of Chicago, Chicago, IL, USA; 5 Institute for Genomic and Systems Biology, University of Chicago, Chicago, IL, USA; 6 The Marine Biological Laboratory, Woods Hole, MA, USA.

## Abstract

Traditional cheeses harbour complex microbial consortia that play an important role in shaping typical sensorial properties. However, the microbial metabolism is considered difficult to control. Microbial community succession and the related gene expression were analysed during ripening of a traditional Italian cheese, identifying parameters that could be modified to accelerate ripening. Afterwards, we modulated ripening conditions and observed consistent changes in microbial community structure and function. We provide concrete evidence of the essential contribution of non-starter lactic acid bacteria in ripening-related activities. An increase in the ripening temperature promoted the expression of genes related to proteolysis, lipolysis and amino acid/lipid catabolism and significantly increases the cheese maturation rate. Moreover, temperature-promoted microbial metabolisms were consistent with the metabolomic profiles of proteins and volatile organic compounds in the cheese. The results clearly indicate how processing-driven microbiome responses can be modulated in order to optimize production efficiency and product quality.

Cheese is a biologically and biochemically dynamic matrix, where the microbiota structure and activity are influenced by manufacture practices and environmental conditions. Traditional cheeses either employ naturally selected starter cultures with extensive genetic diversity, or go without any starter^1^,^2^. Therefore, traditional cheeses harbour a rich and complex microbiota, arising from the use of undefined natural whey cultures (NWCs) used according to a back-slopping procedure, but also from raw milk and from the dairy environment^3^, whose role during manufacture and ripening is recognized but often under-explored. Cheese manufacture is characterized by a succession of different lactic acid bacteria (LAB) species. Curd acidification is mainly driven by thermophilic LAB, mostly *Streptococcus thermophilus*, *Lactobacillus delbrueckii* and *Lb. helveticus*^2^,^4^,^5^, while mesophilic non-starter LAB (NSLAB) take over during the ripening and can be involved in the development of cheese flavour and texture^6^,^7^,^8^,^9^,^10^,^11^. The microbiota plays an important role in shaping the typical sensorial properties of cheese^12^,^13^. Nevertheless, these complex microbial consortia are often difficult to control and their dynamics and evolution during cheese making and ripening cannot be easily predicted. Understanding microbial behaviour during cheese ripening, and how the technological parameters applied can influence the cheese making dynamics and the quality of the final product, are pivotal steps in order to ensure safety and quality^3^,^14^. Moreover, accelerating the ripening kinetics through the modulation of microbial activities can be extremely important for traditional, small-scale, and more industrialized, dairies. With this control they can reduce operating costs, increase the turnover of ripening chambers and optimize the process efficiency having cheese quality as priority. Studying the dynamic microbial ecology and metabolism during cheese making using multi-omic approaches may help to improve efficiency^14^,^15^. Metatranscriptomics has been recently used to study inoculated strain cultures in model cheeses^16^,^17^, yet the transcript dynamics of complex communities remains unexplored. On the other hand, fermented food microbial communities are not so complex as those of other natural environments and may be a replicable and tractable system to study the dynamics of microbial assembly and how they may be manipulated by abiotic factors^18^.

Longitudinal characterisation of the microbial community structure and gene expression was performed during ripening of the traditional Italian Caciocavallo Silano cheese. This is a typical medium-ripened pasta-filata cheese produced with the addition of NWC as natural starter. Initial investigation, reported here, identified parameters that could be modified to accelerate ripening. Therefore, we set up a second experiment, in which the ripening conditions were modulated, and consistent changes in microbial community structure and function were observed. We reveal how increasing ripening temperature affects microbial metabolism, which significantly increases the cheese maturation rate.

## Results

Cheese ripening was explored in two separate experiments. Firstly, microbial community structure (16S rRNA amplicon) analysis and a shallow exploration of gene expression (metatranscriptomics) were performed on raw and thermized cow milk, natural whey culture, curd after 5 hours of fermentation and before stretching, cheeses after brining, and during the ripening at 0, 10, 20, 30 and 60 days. In this way, we identified which bacterial activities/pathways were highly expressed during production and ripening of a pasta-filata cheese, which provided the parameters necessary to understand how to accelerate ripening.

Then, we set up a second experiment, characterising the microbiome across 30 days, for cheeses ripened in three different conditions: (A) standard conditions in the dairy (16 °C and 75% RH), (B) increasing the temperature to 20 °C, and (C) decreasing the RH to 65%.

We obtained a total of 3.3 Gbp in the first experiment, and 19.8 Gbp in the second experiment. In the first experiment, an average of 876,295 reads/sample (±409,657) were obtained, and an average of 22% of reads uniquely mapped to the reference genomes (ranging from 12 to 27.8%).

In the second experiment we obtained an average of 1,180,7044 reads/sample (±5.9 millions) and an average of 35.5% of reads uniquely mapped to the reference genomes (ranging from 23.9 to 58.6%). The normalized abundance (reads per million) of the genes identified for each species and the OTU relative abundance (%) are reported as Supplementary Table S1 and S2.

### Non-Starter Lactic Acid Bacteria (NSLAB) increase in relative abundance both during cheese maturation and with ripening temperature

Cheese ripening was driven by few non-starter lactobacilli, e.g. *Lb. casei* and *Lb. buchneri,* groups whose abundance was greater in the cheese core compared to the crust. This result was consistent in both the experiments carried out in this study. Firmicutes were the phylum that most significantly differentiated the core and crust microbiota (Supplementary Fig. 1). Microbial loads on MRS agar incubated at 30 °C increased during the ripening, with greater values in the cheese core, while thermophilic lactobacilli and streptococci progressively decreased (Supplementary Table 3). When we compared three different ripening conditions (second experiment), NSLAB at 10 days of ripening were significantly more abundant when relative humidity was reduced (C–17.2%) and when ripening temperature was increased (B–15.3%) compared to the control conditions (A–8.3%) (P < 0.05). However, in the reduced relative humidity condition (C), NSLAB abundance rapidly decreased after 10 days of ripening. Interestingly, *Lb. fermentum*, which was not detected in the control condition, appeared in the increased temperature condition (B) after 10 days and continued to increase in abundance throughout ripening.

### Ripening-associated microbial metabolism is greater in the cheese core, increases with temperature, and is consistent with aroma profiles

**Figure 1:**
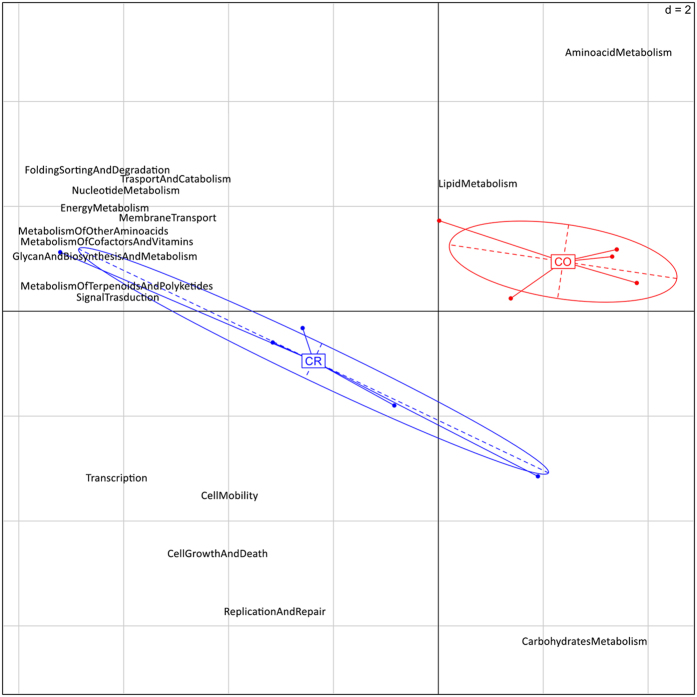
Metatranscriptome drives cheese core and crust separation. PCA based on the abundance of the KEGG annotations at level 2 of hierarchy of the samples of cheese core and crust from the first experiment carried out in this study. The first component (horizontal) accounts for the 65.7% of the variance and the second component (vertical) accounts for the 12.2%. CO, samples of cheese core; CR, samples of cheese crust.

**Figure 2:**
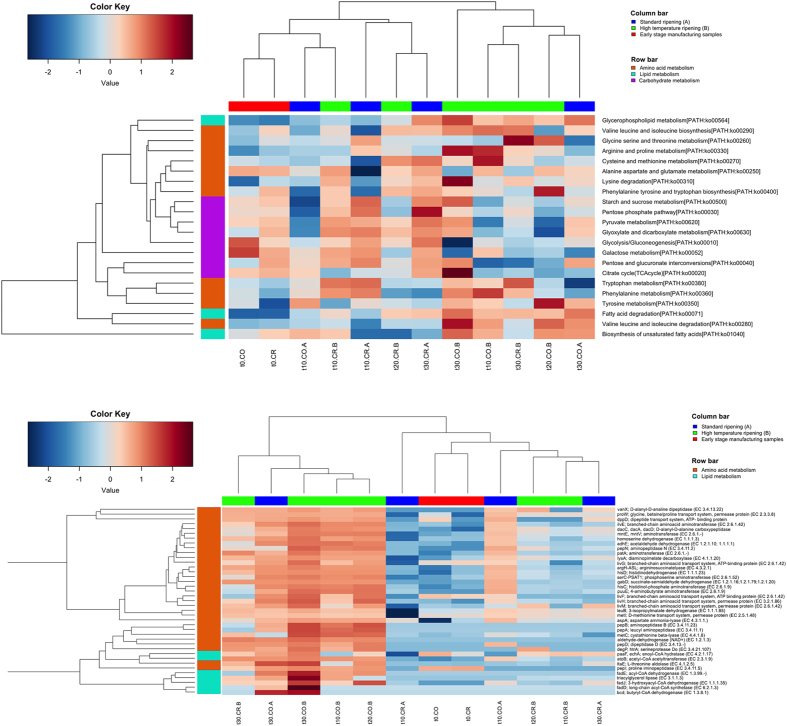
Higher temperature promotes ripening-related gene expression. Average-linkage clustering of samples from the second experiment based on the Euclidean distance of the proportion of the KEGG pathways (**upper**) or genes (**lower**) belonging to carbohydrates (violet), amino acids (orange) and lipids (cyan) metabolisms. Column bar is color-coded as follows: red, cheese at t0; blue, cheese ripened in standard conditions, green, cheese ripened at higher temperature. The color scale represents the scaled abundance of each variable, denoted as Z-score, with red indicating high abundance and blue indicating low abundance. In the sample IDs, the first part indicates the time of ripening (from t0, cheese after brining and drying, to 30 days of ripening); CO, core or CR, crust; A, ripening at standard conditions; B, higher temperature.

**Figure 3:**
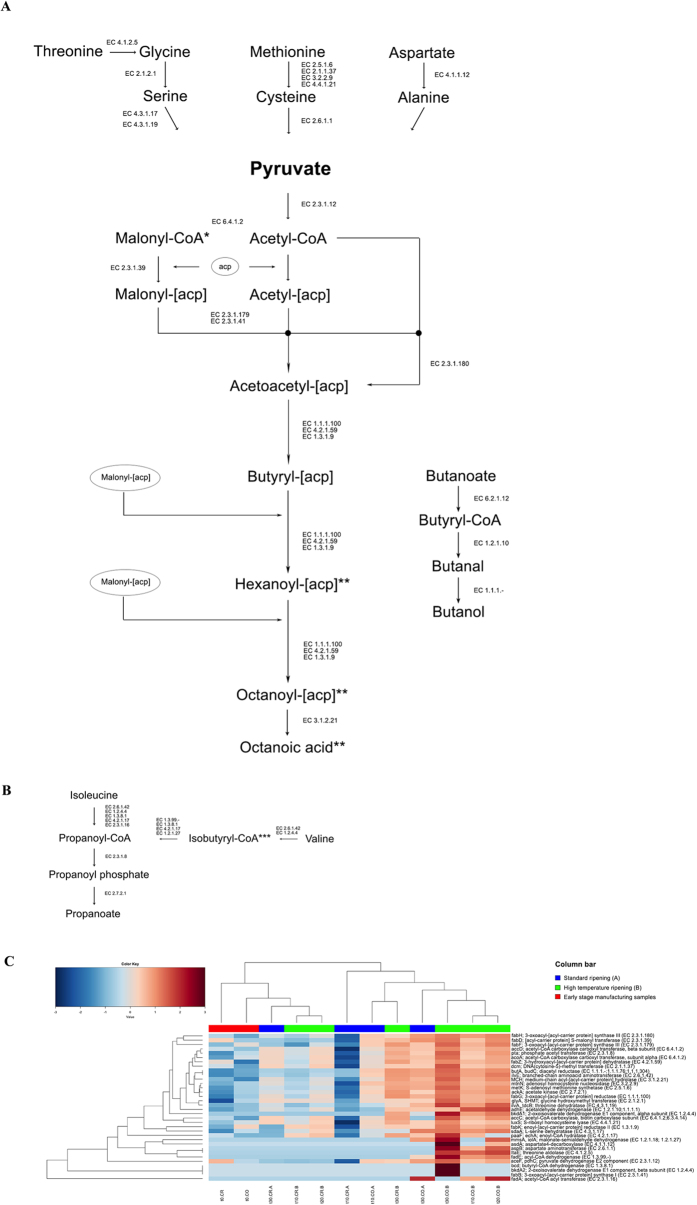
Pathways leading to fatty acids production from amino acids. Biosynthesis of fatty acids from pyruvate (**A**) and from branched-chain amino acids catabolism (**B**). Only KEGG genes identified in the samples analyzed are reported and their abundance is shown in the heatplot (**C**). Average-linkage clustering is based on the Spearman distance of the proportion of the KEGG genes. Only samples from the second experiment are shown. Column bar is color-coded as follows: red, cheese at t0; blue, cheese ripened in standard conditions; green, cheese ripened at higher temperature. The color scale represents the scaled abundance of each variable, denoted as Z-score, with red indicating high abundance and blue indicating low abundance. In the sample IDs, the first part indicates the time of ripening (from t0, cheese after brining and drying, to 30 days of ripening); CO, core or CR, crust; A, ripening at standard conditions; B, higher temperature. *Malonyl-CoA can be substituted by propanoyl-CoA, leading to odd-numbered chain fatty acids. **For each cycle, 2 carbon atoms are added.

The results from the first experiment showed that curd and cheese after moulding and brining were characterized by the prevalence of genes involved in carbohydrate metabolism. During the ripening, carbohydrate metabolism (pentose-phosphate pathway, glycolysis) and cellular division (genetic information processing and core cellular processes) were enriched on cheese crusts, while cheese cores were characterized by significantly elevated amino acid and lipid metabolism (Fig. 1). In addition, samples ripened at higher temperature (B) during the second experiment showed an increase in the expression of pathways related to amino acid and lipid metabolism (Fig. 2a) and associated genes (Fig. 2b) compared to the control (A) at the same ripening times. Genes related to the Leloir pathway of galactose degradation were over-expressed compared to the tagatose-6-phosphate pathway. Enzymes responsible for lactose break-down (*lacZ*, EC 3.2.1.23 and *lacG*, EC 3.2.1.85), Leloir and tagatose pathways showed maximum expression in the early stages of manufacture, and decreased during ripening (Supplementary Fig. 2). Enzymes leading to acetoin and diacetyl production were up-regulated on the crust and had significantly greater expression at the higher temperature (B compared to A; Supplementary Fig. 3). Moreover, a number of peptidases, amino acid and peptide permeases, lipases and genes involved in amino acid catabolism and β-oxidation of fatty acids were over-expressed in the core of cheeses ripened at higher temperature (Fig. 2b and Supplementary Table 4). Finally, higher temperature boosted the expression of genes involved in fatty acids biosynthesis from pyruvate (Fig. 3). The DESeq analysis identified 651 genes differentially expressed between the cheese cores ripened in condition A and B (P value < 0.05) regardless of the ripening time (Supplementary Table 4). Among them, the proteases, peptidases, dipeptide transporters, amino acid permeases, amino acid catabolism genes, fatty acid β-oxidation, and biosynthesis were over-expressed in the core of samples B compared to A (Supplementary Table 4).

**Figure 4:**
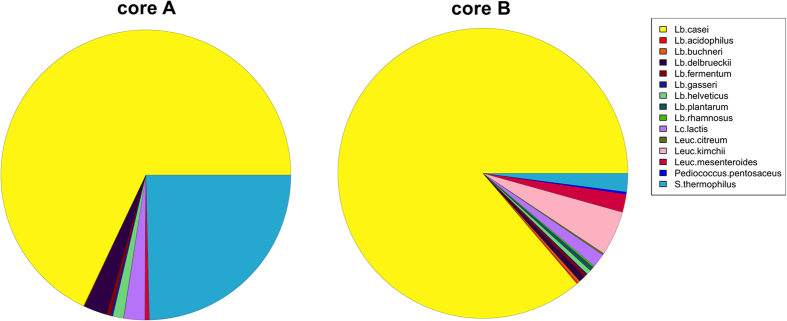
Higher temperature boosts NSLAB amino acid metabolism. Taxonomic assignment of the genes belonging to KEGG amino acid metabolism in the samples of cheese core from the second experiment, at 30 days of ripening. Only species belonging to Firmicutes are reported. A, ripening at standard conditions; B, higher temperature.

**Figure 5:**
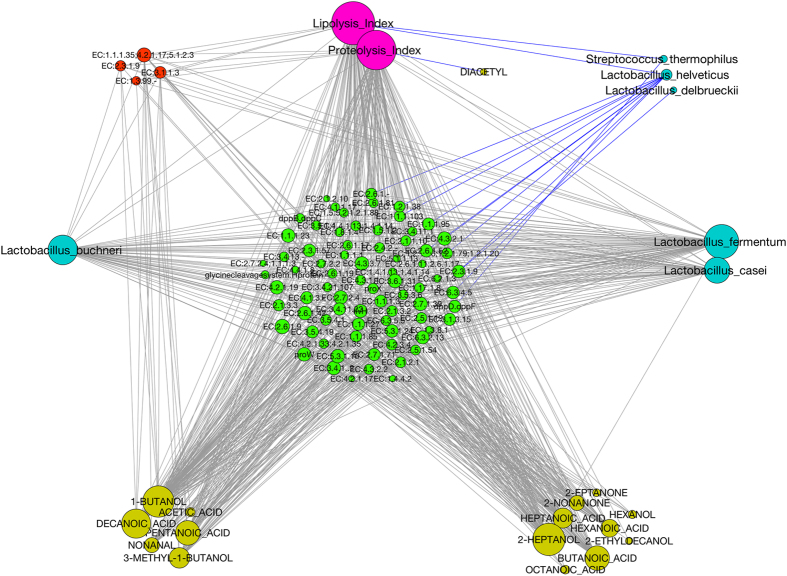
NSLAB abundance, ripening-related gene expression and metabolome are strongly linked. Network showing significant (FDR < 0.1) Spearman’s correlations between KEGG genes belonging to amino acid and lipid metabolism, VOCs, lipolysis and proteolysis indices and OTUs belonging to Firmicutes identified through 16S rRNA sequencing. Node size was made proportional to the number of significant correlations. Edge color indicates negative (blue) or positive (grey) correlations. Node color was assigned as follows: green, KEGG genes related to aminoacid metabolism; red, KEGG genes related to lipid metabolism; yellow, VOCs; magenta, chemical indices; cyan, Firmicutes OTUs.

Amino acid metabolism expression was predominantly associated with the Firmicutes, suggesting a clear effect of temperature on the metabolism of this phylum (Fig. 4). Specifically, the amino acid metabolism expression of *Lb. casei* appeared to be significantly increased under the elevated temperature. However, other NSLAB taxa (such as *Lb. buchneri, Lb. plantarum, Lb. gasseri, Lb. fermentum, Lb. rhamnosus*, *Leuconostoc kimchii*, *Leuc. mesenteroides*, *Leuc. citreum*, *Pediococcus pentosaceus*) also showed increased amino acid metabolism activity at the higher temperature. A network showing significant (FDR < 0.05) Spearman’s correlations between KEGG genes, OTUs and metabolome clearly suggests that the abundance of NSLAB was positively correlated to genes involved in amino acid and fatty acid metabolism, as well as the abundance of volatile compounds, lipolysis and proteolysis indices (Fig. 5). Moreover, ripening-associated activities were inversely related to the abundance of thermophilic LAB from the natural starter (Fig. 5).

The higher ripening temperature also promoted the development of flavour compounds, both in the cheese core and on the crust (Table 1). The metabolomic profiles were highly consistent with the expression profiles from the metatranscriptomic analysis. Volatile short-chain fatty acids (butanoic, pentanoic, hexanoic, heptanoic, octanoic and decanoic acids) were the most abundant volatiles, together with 2-methyl ketones and alcohol compounds, such as hexanol, 2-heptanol, 1-butanol and 3-methyl butanol. Methyl ketones, secondary alcohols and free fatty acids increased in abundance during ripening, and were elevated at the higher temperature, which was also observed for 3-methyl butanol (P < 0.05). Free fatty acids may arise from direct lipolysis of triacylglycerols, or they may be synthesized *ex-novo* from pyruvate evolved from amino acid catabolism (Fig. 3). Lipolysis and proteolysis indices were greater in the core, and increased in cheese ripened at higher temperature (Table 1). Accordingly, LC/MS analysis of the pH 4.6 insoluble nitrogen fraction showed an extensive degradation of either αs1- and β-casein in the cheese ripened at higher temperature, while the pH 4.6-soluble fraction showed a greater amount of soluble peptides derived from the two proteins (Supplementary Fig. 4).

## Discussion

The cheese ripening process is very complex and involves microbiological and biochemical changes leading to the development of a specific flavour and texture. The cheese microbiome is shaped by the abiotic factors encountered during ripening (pH, a_w_, NaCl concentration, O_2_ availability). Therefore, different dynamics may occur in the cheese core and crust, due to the different environmental conditions^3^,^19^. The importance of fungi- and bacteria-associated metabolic activities in surface-ripened cheeses was recently highlighted^16^,^17^,^20^, while an increase in NSLAB in ripened cheeses was previously observed^7^,^10^. NSLAB are known as proteolytic^21^,^22^,^23^, and the adjunct of selected cultures was shown to increase proteolysis levels^8^,^24^,^25^, suggesting they may play a key role during cheese ripening. We provide concrete evidence of their essential contribution, showing that a different structure and functionality of the microbiome evolves in cheese core and crust, and that the ripening proceeds with a gradient starting from the core towards the crust, driven by NSLAB succession. On the cheese surface, where lower a_w_ and higher O_2_ concentrations make the environment adverse, NSLAB development is delayed and the cellular metabolism is directed towards multiplication and energy production from carbohydrates. Consistently, lactose break-down and galactose metabolism were retarded on the cheese crust, and their levels kept constantly higher on the crust compared to the core. Moreover, microbial activities are strongly influenced by the conditions applied during ripening. We cannot exclusively link the higher proteolysis levels to bacterial activity, and endogenous enzymes may be still active in cheese without a curd-cooking step. However, a clear bacterial contribution to ripening activities is supported by the consistency between metatranscriptome and metabolome data, and by the significant degree of correlation between the occurrence of NSLAB, expression profiles and metabolic patterns (Fig. 5). Particularly, bacterial gene expression of ripening-associated activities is significantly linked to metabolite profiles and proteolysis and lipolysis indices.

The increase in proteolysis, lipolysis and amino acids/fatty acids catabolism boosted by the higher temperature definitely promoted the production of volatile compounds. The observed free fatty acids may arise from direct lipolysis of triacylglycerols, as well as from *ex novo* biosynthesis from pyruvate produced by amino acid catabolism^26^. Moreover, 3-methyl butanol is produced by leucine degradation, and methyl ketones and secondary alcohols come from fatty acids catabolism^27^,^28^.

Recently, the importance of exploring microbial assembly mechanisms in fermented foods (MCoFFs) was highlighted^18^. Fermented foods give tantalizing opportunities to study microbial metabolic patterns and ecological dynamics, identifying the major drivers and testing hypotheses that can be easily extrapolated to more complex environments^18^. We give a clear example of how they might be used to understand the effect of abiotic factors on microbial community assembly and metabolism. This supports the potential for modelling microbial community dynamics in more complex environments^18^.

The increase in ripening temperature promotes the expression of genes related to proteolysis and amino acid/lipid transport and catabolism, leading to a higher concentration of free amino acids and fatty acids and boosting the production of VOC typically found in Caciocavallo cheese volatilome^7^, possibly accelerating the ripening and enhancing the potential flavour impact of the cheese. Indeed, cheeses ripened at higher temperature for only 20 days had very similar transcriptomic profiles as those ripened for longer at standard temperature, indicating that the ripening-related activity is boosted by the change in ripening conditions. Finally, taxonomic assignment of genes related to KEGG amino acid metabolism pathways confirmed a shift in the amino acid metabolism due to the higher temperature and emphasized the role of NSLAB as fundamental players in cheese maturation.

The results clearly indicate how changes in ripening conditions are important to manipulate cheese microbiome and its activities and that the response of the microbiome can be promptly used to improve processing conditions and product quality.

## Methods

### Cheese manufacture and sampling

The cheese manufacture and ripening was carried out in a dairy located in Southern Italy and producing Caciocavallo Silano cheese granted the Protected Designation of Origin (PDO, CE Reg. 1263/96). In a first experiment, raw and thermized cow milk, NWC, curd after 5 h of fermentation and before stretching (when pH reached ca. 5.25), cheeses after molding, after brining and during the ripening (at 10, 20, 30 and 60 days) were collected and analyzed through 16S rRNA sequencing and RNA-seq (Supplementary Table 5). In a second experiment, in order to evaluate the effect of ripening parameters on the microbiome, we decided to ripe the cheeses from the same manufacturing in three different conditions: a control ripening (A) by using the standard conditions in the dairy (16 °C and 75% RH, condition A), increasing the temperature to 20 °C (B) or decreasing the RH to 65% (C). In this second experiment, the ripened cheeses were collected up to 30 days. Sample IDs reported in Supplementary Table 5 and throughout the text and the figures were assigned as follows: the first part of the ID indicates the time of ripening (from t0, cheese after brining and drying, to 60 days of ripening); CO, core or CR, crust; A, ripening in standard conditions, B, ripening at higher temperature, C, ripening at lower RH.

Water activity (a_w_) was measured by using a HygroPalm23-AW instrument (Rotronic AG, Basserdorf).

Samples from the core (the inner part, collected in the middle of the cheese) and crust (the outer part, after peeling the external layer) were cut (in slices of ~0.5 cm thick) in sterile conditions and analyzed separately. Different aliquots from each sample were collected in sterile plastic bags for the different analyses and transported to the laboratory under refrigerated conditions (4 °C). RNAlater (Ambion, Foster City, California) was added in 1:6 ratio to the aliquot to be used for RNA extraction before transport. Samples were stored at −80 °C prior to analyses, except for microbial counts, that were carried out within 2 h from the sampling, as follows.

Twenty-five grams (solid) or 25 ml (liquid) of sample at each time of sampling and under each ripening condition were homogenized in 225 ml of quarter-strength Ringer’s solution (Oxoid, Milan, Italy) for 2 min in a stomacher (LAB Blender 400) by using Sto-Circul-Bag stomacher bags (both from PBI, Milan, Italy) at room temperature. Decimal dilutions in quarter-strength Ringer’s solution were prepared, and 1 ml aliquots of the appropriate dilutions were inoculated in triplicate in M17 or MRS agar (Oxoid, Milan, Italy) and incubated aerobically and anaerobically, respectively, at 30 or 42 °C for 48 h to obtain the viable counts of mesophilic and thermophilic streptococci or lactobacilli. Results were calculated as the means of log colony forming units (CFU)/g for three independent determinations.

### Samples preparation and RNA extraction

Samples stored in RNA later were homogenized and 10 ml of the mixture was centrifuged for 10 min at 4 °C (12,000 × g). Two biological replicates (two different cheeses at each sampling point and for each condition) were subjected to RNA extraction and total RNA was pooled before further processing. The pellet was washed twice in PBS (Phosphate-Buffered Saline, pH 7.4) and RNA extraction was carried out in duplicate by using the PowerMicrobiome RNA Isolation kit (MoBio, Carlsbad, California). DNA was removed by a treatment with TURBO-DNase (Ambion, Foster City, California) for 3 h at 37 °C. The absence of DNA was checked by PCR and the treatment repeated if necessary. The quality of the RNA was checked by agarose gel electrophoresis and by the 2100 Bioanalyzer (Agilent Technologies, Palo Alto, California). The Qubit and the Qubit RNA Assay kit (Life Technologies, Carlsbad, California) were used for quantification.

### 16S rRNA library preparation and amplicon sequencing

Complimentary DNA (cDNA) was synthetized by using the High-Capacity cDNA Reverse Transcription kit (Applied Biosystems, Carlsbad, California), starting from 200 ng of total RNA. The microbial diversity was studied by pyrosequencing of the amplified V1–V3 region of the 16S rRNA as reported elsewhere^29^. PCR conditions, library preparation and sequencing were carried out as recently described^30^.

### Ribosomal RNA depletion, library preparation and shotgun metatranscriptome sequencing

Metatranscriptome was studied for all the samples from the first experiment and for selected samples for the second experiment (Supplementary Table 3). Two replicates were sequenced for all samples (each replicate was obtained by pooling the RNA extracted from two different cheeses). Ribosomal RNA (rRNA) was depleted by using the Ribo-Zero Magnetic kit (Epicentre, Charlotte, North Carolina) and the mRNA enriched RNA was purified by Agencourt RNAClean XP (Beckman Coulter, Milano, Italy) following the manufacturer’s instruction. Then, library preparation and sample multiplexing were carried out by using the ScriptSeq v2 RNA-Seq Library Preparation Kit (Epicentre, Charlotte, North Carolina) (insert size around 300 bp). The quality of the library was checked by 2100 Bioanalyzer (Agilent Technologies, Palo Alto, California). Sequencing was carried out on a Next Seq 500 Sequencer with the Mid Output Kit (both from Illumina, San Diego, California), yielding 150 bp single-end reads.

### Bioinformatics and data analysis

16S rRNA amplicon reads were analyzed by using QIIME 1.9.0 software^31^, as previously reported^30^. OTUs defined by a 99% of similarity were picked using the uclust pipeline^32^ and the representative sequences were submitted to the RDPII classifier^33^ to obtain the taxonomy assignment and the relative abundance of each OTU using the Greengenes 16S rRNA gene database^34^.

The whole metatranscriptome data analysis was carried out as follows: raw reads quality (Phred scores) was evaluated by using the FastQC toolkit (http://www.bioinformatics.babraham.ac.uk/projects/fastqc/). Adaptor and primer contamination was eliminated with CutAdapt^35^. Then, low quality bases (Phred score <20) were trimmed and reads shorter than 60 bp were discarded with the SolexaQA++ software^36^. Reads were aligned to a reference database by using Bowtie2^37^ in end-to-end, sensitive mode. The database used was built downloading the protein coding portions of the genomes (.ffn files) from the NCBI RefSeq database (ftp://ftp.ncbi.nlm.nih.gov/genomes/ASSEMBLY_BACTERIA/) and from http://patricbrc.org/portal/. The species included were chosen according to the 16S sequencing results and picking species commonly found in food ecosystems (listed in Supplementary Table 6). Since we did not carry out metagenomics and we did not have the genomes of strains directly isolated from those samples, all the available sequenced genomes were included. The concatenated .ffn files were aligned against the Kyoto Encyclopedia of Genes and Genomes (KEGG) database^38^ version April 2011 by using mblastx^39^ in order to obtain the functional annotation and the gene taxonomy. The number of reads uniquely mapped to each gene in the database was extracted by using SAMtools^40^ and normalized according to the library size using custom scripts built under R environment (www.r-project.org). Only genes to which at least 5 reads/sample mapped were kept for subsequent analyses. Statistical analysis and plotting were carried out in R environment. Differential gene expression analysis was done by using the Bioconductor package *DESeq*^41^. P-values were adjusted for multiple testing using the Benjamini-Hochberg procedure^42^ and a false discovery rate (FDR) <0.05 considered as significant. Pairwise Spearman’s correlations between OTUs, KEGG genes, volatile organic compounds and biochemical indices were computed by using the R package *psych* and the significant ones (FDR < 0.05) were plotted in a correlative network by using Cytoscape v. 2.8.1^43^. Principal Component Analysis (PCA) and Hierarchical Clustering were carried out by using the *made4* package in R. All the results are reported as mean values of two replicates.

### Extraction and analysis of volatile compounds by SPME-GC/MS

The extraction of volatile compounds was performed according to Lee *et al.*^44^. The frozen cheese was finely grated and 25 g were transferred into 100 mL bottle, with 25 mL of deionised water, 50 μL of 2-methyl-3-heptenone (99% purity, Sigma-Aldrich, St. Louis, Missouri) as internal standard (950 mg L^−1^) and 12.5 g of sodium phosphate (NaH_2_PO_4_). The bottle was kept at 50 °C for 10 min in a water bath to melt the cheese. Then melted cheese was magnetically stirred for 20 min at 50 °C to accelerate the equilibrium of the volatile compounds in the headspace. The SPME fiber (50/30 μm thickness divinyl-benzene/carboxen/polydimethylsiloxane, 2 cm; Sigma-Aldrich, St. Louis, Missouri) was inserted through a Teflon septum in the bottle and exposed to sample headspace for 30 min at 40 °C during stirring. The same type of fiber was reported to be highly efficient for cheese aroma analysis^45^. Volatile compounds were analyzed by GC coupled with a mass spectrometer using a GC/MS Hewlett-Packard 6890N (Agilent Technologies, Palo Alto, California) equipped with a J&W HP-5MS capillary column (30 m × 0,25 mm i.d. × 0,25 μm Film Thickness; J&W Scientific, Folsom, California). Temperature was set at 40 °C for 2 min and increased from 40 to 160 °C at the rate of 6 °C/min and from 160 to 210 °C min^−1^ at 10 °C min^−1^. The injector was kept at 250 °C. Helium was used as carrier gas (0.9 mL min^−1^). Mass spectra were recorded at 70 eV. The source temperature was 230 °C and the interface temperature was 250 °C. Volatile compounds thermal desorption was carried out by exposing the SPME fiber in the injector for 10 min. Compounds identification was performed by comparing retention times and mass spectra with those of pure reference compounds in the same conditions and with those of NIST database. Prior to use, the fiber was conditioned at 270 °C for 1 h. A blank test was carried out before each analysis to prevent the release of undesirable compounds. The quantitative analysis was carried out by normalizing the peak area of each compound respect to the area of the peak of the internal standard. Peak areas were processed by the software Chemstation (Agilent Technologies, Palo Alto, California). The analysis was performed in triplicate.

Significant differences among the different samples were determined for each compound by one-way ANOVA statistical analysis. Tukey’s test was used to discriminate among the means of the variables. Differences with P < 0.05 were considered significant. The data elaboration was carried out using XLStat (version 2009.03.02), an add-in software package for Microsoft Excel (Addinsoft Corp., Paris, France).

### Proteolysis and lipolysis analyses

Grated cheese (5 g) was dispersed in 100 mL 0.4 M sodium citrate buffer (pH 8.0) previously heated to 40 °C and homogenised for 1 min with an Ultra-Turrax apparatus (Ultra-Turrax model T25, Ika Labortechnik, Staufen, Germany). The degree of hydrolysis (DH) was determined with the 2,4,6-trinitrobenzene sulfonic acid method, according to Hermanson^46^ with the modifications reported by Picariello *et al.*^47^. For LC-MS analysis, the pH of the citrate soluble fraction was adjusted to 4.6. The resulting suspension was centrifuged for 10 min at 3000 g, the fat layer removed, and the insoluble and soluble fractions were separated. The two fractions were then analyzed by LC/ESI-Q/TOF MS/MS using a Q TOF Ultima hybrid mass spectrometer (Waters, Manchester, UK), equipped with an ESI source and operating in positive ion mode, as previously reported^48^.

The lipolysis index was expressed as the quantity of KOH (in mg) required to neutralize the free fatty acids contained in 1 g of sample. Twenty grams of each frozen sample were finely grated and three grams of the grated cheese were incubated for 30 min with 50 ml of ethanol/diethyl ether (1:1 v/v) while stirring, in order to extract the fat. The suspension was filtered through Watman paper n° 42 and the acidity of the filtrate was measured by titration as described by Dugat-Bony *et al.*^16^. Each sample was analysed in triplicate.

### Sequences accession numbers

The 16S rRNA and the metatranscriptome sequences are available at the Sequence Read Archive (SRA) of the National Center for Biotechnology Information (NCBI) under accession numbers SRP061555 and SRP061556, respectively.

## Additional Information

**How to cite this article**: De Filippis, F. *et al.* Metatranscriptomics reveals temperature-driven functional changes in microbiome impacting cheese maturation rate. *Sci. Rep.* **6**, 21871; doi: 10.1038/srep21871 (2016).

## Supplementary Material

Supplementary Information

## Figures and Tables

**Table 1:** Volatile compounds (μg/kg) and chemical indices determined on the cheese samples analysed in the second experiment carried out in this study.

	t0.CR	t10.CR.A	t10.CR.B	t20.CR.B	t30.CR.A	t30.CR.B
Volatile compound						
Acetic acid	135.4 ± 5.0^a,A^	293.8 ± 90.4^a*^	726.4 ± 195.1^A*^	2793.0 ± 559.0^A^	9930.0 ± 1951.3^b^	9097.9 ± 3956.9^B^
Diacetyl	51.3 ± 6.2^a,A^	58.7 ± 2.4^a*^	tr^*^	tr	tr	tr
1-Butanol	nf	nf	nf	nf	nf	nf
Acetoin	245.7 ± 26.2^a,A^	214.7 ± 9.8^a*^	437.7 ± 78.3^A*^	803.7 ± 478.2^A^	1664.8 ± 382.4^b^	1179.8 ± 537.5^A^
3-Methyl-1-butanol	31.4 ± 1.2^a,A^	29.4 ± 5.4^a*^	56.8 ± 11.5^A*^	64.1 ± 10.0^A^	222.5 ± 34.7^b^	177.8 ± 62.8^B^
Butanoic acid	1792.9 ± 363.3^a,A^	8575.0 ± 705.6^a*^	23298.5 ± 969.1^A*^	101533.7 ± 50545.2^B^	214814.4 ± 49100.2^b^	222723.7 ± 81019.1^B^
Hexanol	nf	nf	nf	nf	nf	nf
Pentanoic acid	26.2 ± 0.5^a,A^	92.4 ± 8.7^a*^	241.2 ± 25.8^A.B*^	1310.4 ± 679.4^B^	2525.4 ± 878.9^b^	2753.8 ± 692.0^C^
2-Heptanone	32.0 ± 7.8^a,A^	81.6 ± 16.8^a*^	314.8 ± 90.2^B^	1583.4 ± 452.6^C^	1467.8 ± 254.2^b*^	4076.3 ± 1144.1^D^
2-Heptanol	nf	nf	nf	nf	nf	nf
Hexanoic acid	856.9 ± 80.6^a,A^	4560.7 ± 300.4^b*^	13607.7 ± 1201.5^B*^	38264.0 ± 16106.1^B^	60861.2 ± 21869.7^c^	91962.6 ± 26958.0^C^
Heptanoic acid	tr	21.3 ± 0.5a^*^	59.5 ± 10.2^A*^	258.4 ± 92.8^B^	474.0 ± 106.1^b^	544.0 ± 169.7^C^
2-Nonanone	nf	17.0 ± 0.5^a*^	75.0 ± 12.1^B*^	440.8 ± 146.3^C^	485.5 ± 53.1^b*^	1295.5 ± 299.3^D*^
Nonanal	10.8 ± 0.3^a,A^	19.5 ± 1.8^a*^	57.0 ± 10.2^B*^	177.1 ± 99.3B^C^	230.5 ± 62.3^b^	428.0 ± 168.4^D^
Octanoic acid	60.7 ± 14.7^a,A^	211.2 ± 45.0^b*^	527.8 ± 68.5^B*^	2854.3 ± 834.9^C^	2665.5 ± 545.5^c*^	6773.4 ± 1867.3^D*^
Ethyl octanoate	nf	nf	nf	nf	nf	nf
Decanoic acid	12.4 ± 3.1^a,A^	30.2 ± 0.3^b*^	105.3 ± 28.0^B*^	414.3 ± 89.6^C^	466.3 ± 125.2^c^	941.3 ± 320.7^D^
Methyl dodecanoate	11.6 ± 0.3^a,A^	15.4 ± 0.6^a*^	52.9 ± 3.2^B*^	85.6 ± 23.8^B^	116.9 ± 14.2^b^	126.3 ± 48.7^C^
Chemical determinations						
Proteolysis index	2.76 ± 0.23^a,A^	4.15 ± 0.16^b*^	5.71 ± 0.24^B*^	10.74 ± 0.58^C^	8.75 ± 0.39^c*^	11.59 ± 0.34^D*^
Lipolysis index	1.57 ± 0.04^a,A^	2.03 ± 0.04^b*^	2.28 ± 0.06^B*^	2.57 ± 0.07^C^	2.10 ± 0.06^b*^	2.66 ± 0.18^C*^
a_w_	0.965 ± 0.003^a,A^	0.950 ± 0.004^b*^	0.940 ± 0.001^B*^	0.927 ± 0.004^C^	0.907 ± 0.006^c^	0.890 ± 0.003^D^
pH	5.50 ± 0.02^a,A^	5.79 ± 0.09^b^	5.80 ± 0.03^B^	5.88 ± 0.04^C^	5.15 ± 0.06^c*^	5.79 ± 0.09^D*^
Volatile compound						
Acetic acid	171.0 ± 25.2^a,A^	234.8 ± 58.5^b*^	1653.8 ± 67.7^B*^	7710.1 ± 593.8^C^	2569.2 ± 364.0^c*^	12136.3 ± 1377.7^D*^
Diacetyl	44.3 ± 5.4^a^	65.0 ± 5.0^b*^	tr^*^	tr	tr	tr
1-Butanol	tr	tr	tr	202.4 ± 20.2^B^	85.5 ± 14.7^b*^	666.9 ± 153.8^C*^
Acetoin	207.7 ± 21.4^a,A^	92.0 ± 13.0^b*^	nf^*^	nf	nf	nf
3-Methyl-1-butanol	33.4 ± 3.5^a,A^	27.8 ± 3.3^a*^	48.3 ± 0.9^A*^	74.1 ± 15.2^B^	97.3 ± 1.1^b*^	133.4 ± 17.7^C*^
Butanoic acid	1910.0 ± 32.0^a,A^	6346.7 ± 87.2^b*^	17209.7 ± 732.5^B*^	49756.5 ± 3872.5^C^	42067.4 ± 1851.9^c*^	104098.6 ± 17061.3^D*^
Hexanol	nf	nf	nf	115.5 ± 8.5^A^	nf^*^	608.7 ± 83.7^B*^
Pentanoic acid	20.3 ± 0.7^a,A^	74.8 ± 8.6^b*^	208.2 ± 20.9^B*^	600.5 ± 18.3^C^	509.6 ± 18.5^c*^	1401.9 ± 366.3^D*^
2-Heptanone	44.2 ± 6.6^a,A^	83.5 ± 19.6^b^	100.2 ± 6.2^B^	211.3 ± 4.9^C^	384.1 ± 14.4^c^	463.3 ± 118.0^D^
2-Heptanol	nf	nf	49.6 ± 13.0^A^	169.4 ± 9.8^B^	110.4 ± 2.7^b*^	308.7 ± 53.3^C*^
Hexanoic acid	2776.8 ± 661.4^a,A^	4538.2 ± 376.1^b*^	11765.9 ± 616.1^B*^	29079.4 ± 1501.4^C^	21978.8 ± 1405.4^c*^	68903.8 ± 20525.5^D*^
Heptanoic acid	20.0 ± 3.4^a,A^	32.6 ± 7.1^a^	44.2 ± 11.1^B^	199.0 ± 26.3^C^	198.8 ± 30.9^b*^	426.4 ± 82.0^D*^
2-Nonanone	nf	27.1 ± 0.3^b*^	47.0 ± 6.0^B*^	127.2 ± 15.9^C^	165.1 ± 18.5^c*^	225.5 ± 14.8^D*^
Nonanal	15.1 ± 3.1^a,A^	28.5 ± 12.4^a^	32.2 ± 7.6^B^	74.4 ± 15.7^C^	76.6 ± 9.6^b*^	163.8 ± 29.4^D*^
Octanoic acid	98.6 ± 17.1^a,A^	216.6 ± 26.9^b*^	596.8 ± 63.7^B*^	2132.9 ± 159.6^C^	2044.7 ± 412.2^c*^	5127.1 ± 1364.4^D*^
Ethyl octanoate	nf	nf	nf	nf	nf^*^	190.5 ± 28.5^*^
Decanoic acid	16.9 ± 4.3^a,A^	34.7 ± 5.3^b*^	105.0 ± 8.0^B*^	311.3 ± 73.6^C^	293.5 ± 36.4^c*^	752.2 ± 175.1^D*^
Methyl dodecanoate	11.9 ± 2.9^a,A^	13.3 ± 0.2^a*^	31.7 ± 4.8^B*^	44.0 ± 1.7^C^	64.1 ± 13.2^b*^	156.8 ± 24.8^D*^
Chemical determinations						
Proteolysis index	2.98 ± 0.45^a,A^	4.18 ± 0.06^b*^	5.78 ± 0.31^B*^	10.02 ± 0.28^C^	7.18 ± 0.17^c*^	11.96 ± 0.47^D*^
Lipolysis index	1.63 ± 0.03^a,A^	2.75 ± 0.04^b*^	3.03 ± 0.03^B*^	3.65 ± 0.06^C^	2.94 ± 0.03^c*^	4.18 ± 0.07^D*^
a_w_	0.968 ± 0.001^a,A^	0.961 ± 0.005^b^	0.959 ± 0.004^B^	0.958 ± 0.004^c*^	0.951 ± 0.003^C^	0.949 ± 0.003^D^
pH	6.02 ± 0.05^a,A^	5.50 ± 0.06^b^	5.56 ± 0.10^B^	5.80 ± 0.02^C^	5.60 ± 0.05^b*^	5.78 ± 0.09^C*^

nf: not found.

tr: traces.

Different letters indicate significant differences (p < 0.05) between core or crust samples at different ripening time (capital letters, samples B; lowercase letters, samples A). Asterisk indicates significant differences between A and B samples at the same ripening time.
