# Supplementary material for: Metatranscriptomics reveals temperature-driven functional changes in microbiome impacting cheese maturation rate
Source: Sci Rep. 2016 Feb 25;6:21871. doi: 10.1038/srep21871 (PMC4766472; doi:10.1038/srep21871)
Supplement: Supplementary Information [file srep21871-s1.pdf]

Supplementary Material

Metatranscriptomics reveals temperature-driven functional changes in microbiome impacting cheese maturation rate

Francesca De Filippis[1], Alessandro Genovese[1], Pasquale Ferranti[1], Jack A. Gilbert[2,3,4,5,6], Danilo Ercolini[1*]

[1]Department of Agricultural Sciences, University of Naples Federico II, Via Università 100, 80055 Portici, Italy

[2]Biosciences Division (BIO), Argonne National Laboratory, Argonne, IL;

[3]Departmen of Ecology & Evolution, University of Chicago, Chicago, IL;

[4]Department of Surgery, University of Chicago, Chicago, IL;

[5]Institute for Genomic and Systems Biology, University of Chicago, Chicago, IL.;

[6]The Marine Biological Laboratory, Woods Hole, MA.

*Corresponding author.

Department of Agricultural Sciences, Division of Microbiology, University of Naples Federico II, Via Università 100, 80055 Portici, Italy

Phone: +39 081 2539449. Fax: +39 081 2539407.

E-mail: ercolini@unina.it.

Figure S1 – Cheese core and crust have different microbiota. Average-linkage clustering based on the Spearman correlation coefficients of the proportion of OTUs belonging to the Firmicutes *phylum* identified in the samples analysed in the first (A) and second (B) experiment carried out in this study. Only OTUs with abundance > 0.1% in at least 1 sample were included. The color scale represents the scaled abundance of each variable, denoted as Z-score, with red indicating high abundance and blue indicating low abundance. Column bar is coloured according to sample type: red, intermediates of production; blue, cheese core; green, cheese crust. In the sample IDs, the first part indicates the time of ripening (from t0, cheese after brining and drying, to 60 or 30 days of ripening, in the first and second experiment, respectively); CO, core or CR, crust; A, ripening at standard conditions; B, higher temperature; C, lower RH.

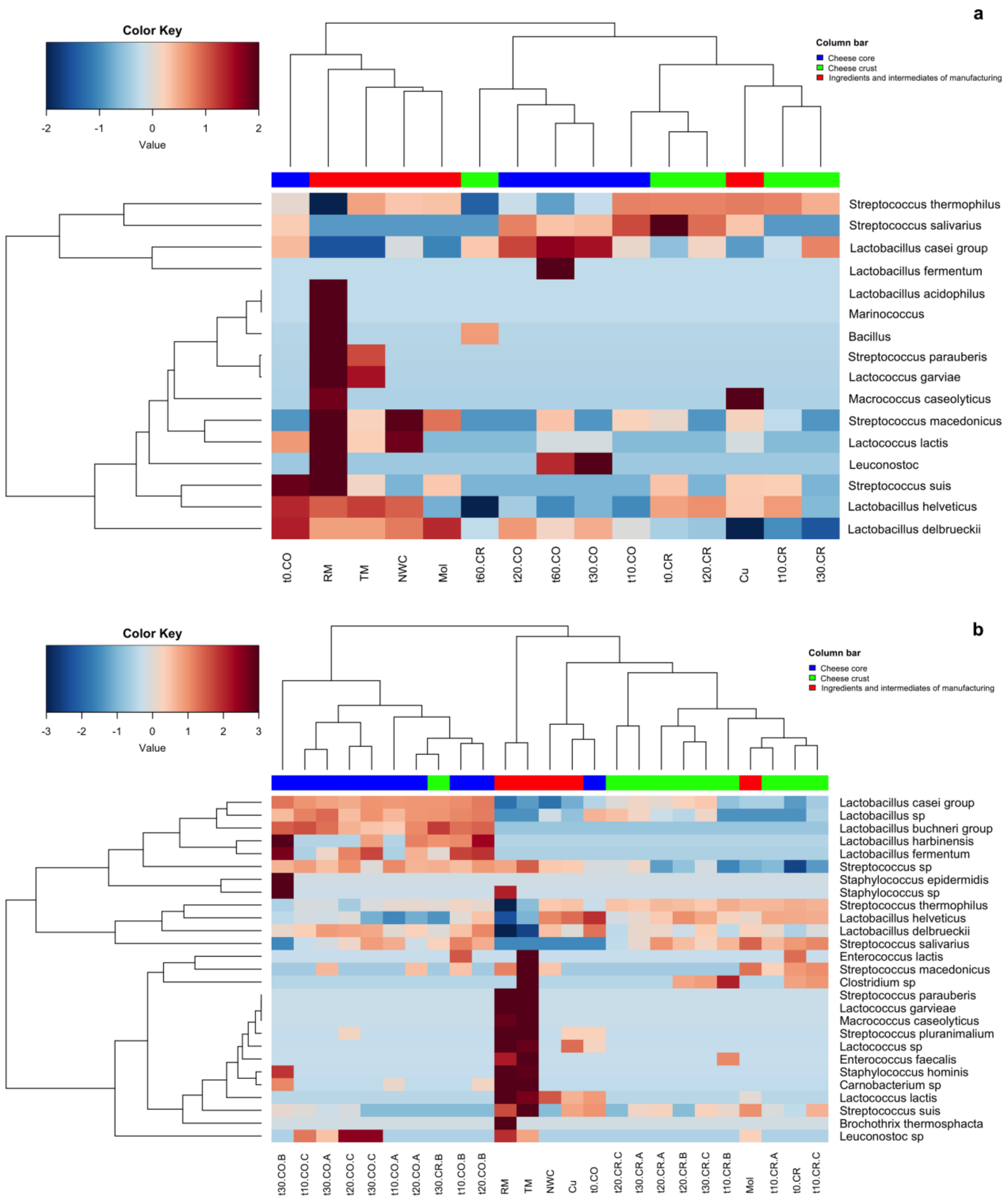

Figure S2 – Gene expression in galactose catabolism pathways. Leloir and tagatose 6P pathways with related expression data in the samples of cheese core and crust analyzed in the second experiment. In the sample IDs, the first part indicates the time of ripening (from t0, cheese after brining and drying, to 30 days of ripening); CO, core or CR, crust; A, ripening at standard conditions; B, higher temperature. Only samples from the second experiment are shown.

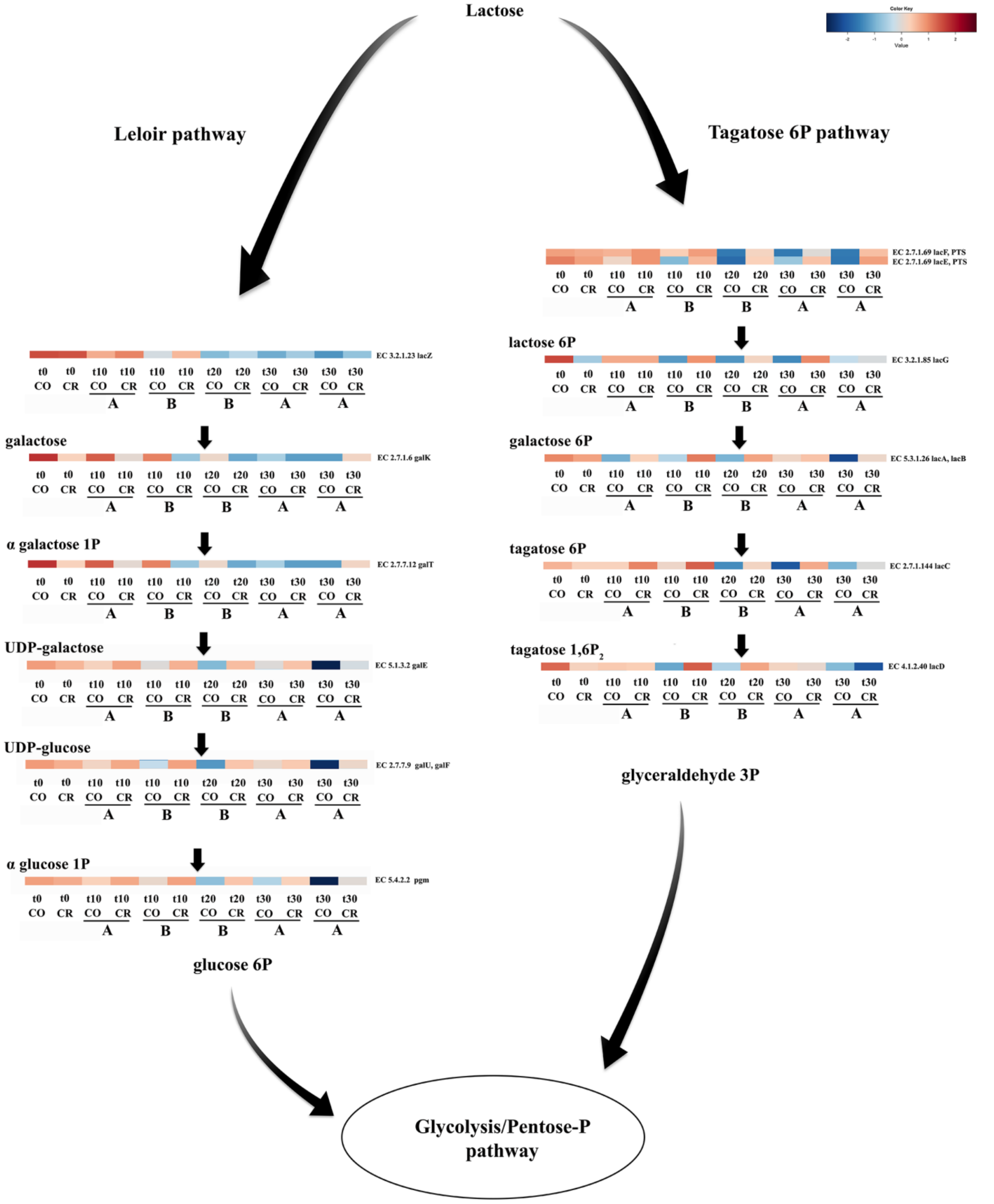

Figure S3 - Gene expression in pyruvate catabolism pathways. Acetoin and lactate production from piruvate, with related gene expression data in the samples of cheese core and crust analyzed in the second experiment. Dashed line indicates chemical reaction. Only samples from the second experiment are shown. In the sample IDs, the first part indicates the time of ripening (from t0, cheese after brining and drying, to 30 days of ripening); CO, core or CR, crust; A, ripening at standard conditions; B, higher temperature.

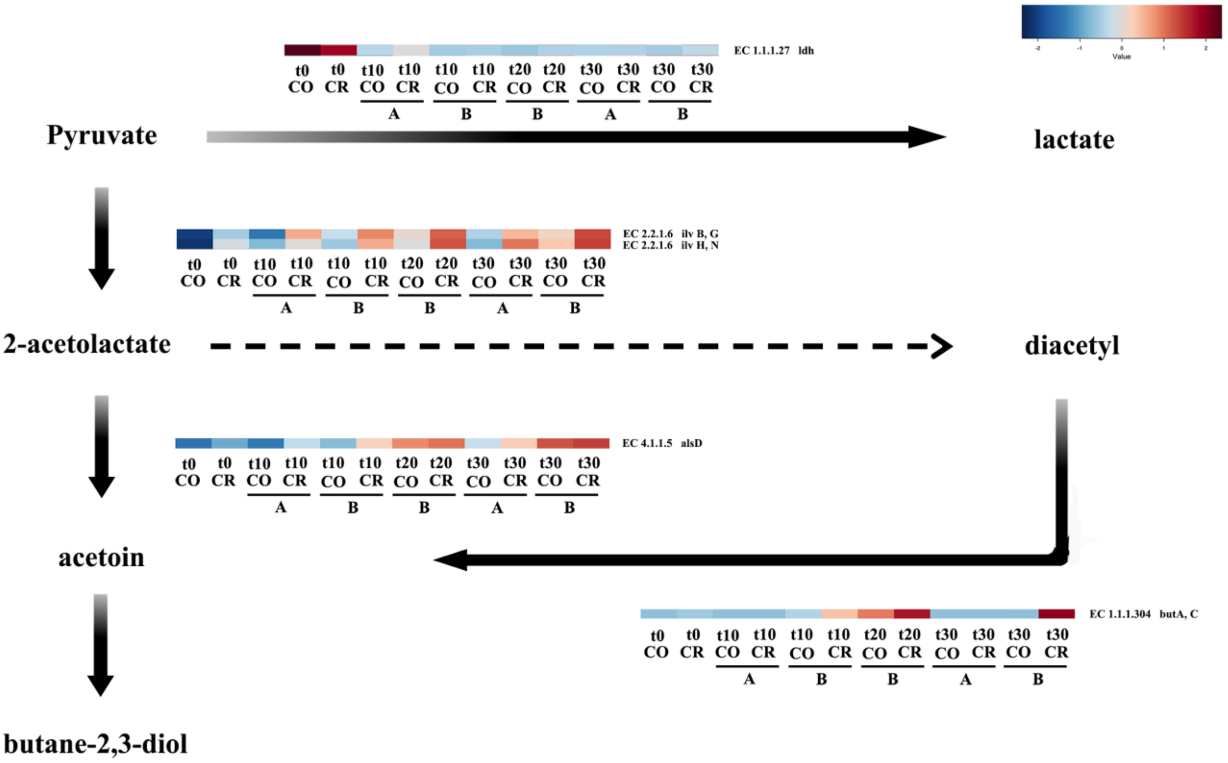

Figure S4 – **Higher temperature increases proteolysis levels.** LC/MS analysis of the pH 4.6 soluble nitrogen fraction from Caciocavallo cheese core at 30 days of ripening. A (dashed line), cheese ripened at standard conditions; B (continuous line), cheese ripened at higher temperature.

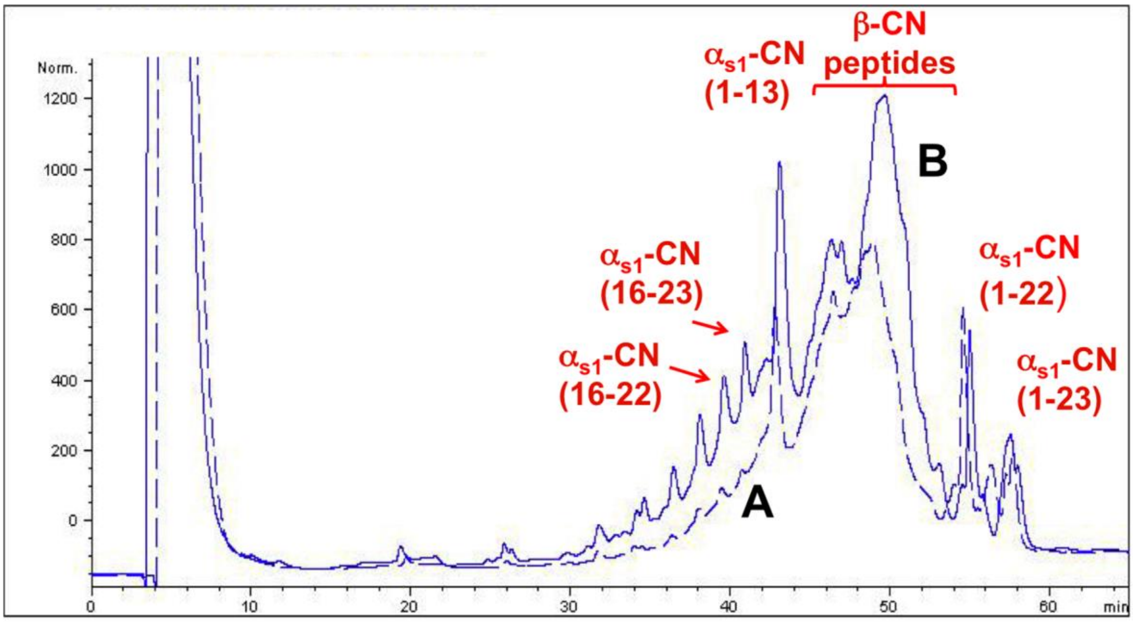

Table S3. Viable counts of presumptive lactobacilli and streptococci in the samples analyzed in the second experiment carried out in this study.

	Log CFU/g ± SD			
**Sample**	Mesophilic lactobacilli	Thermophilic lactobacilli	Mesophilic cocci	Thermophilic cocci
	MRS agar (30°C)	*MRS agar* (42°C)	*M17 agar* (30°C)	*M17 agar* (42°C)
**RM**	5.17±0.32	5.30±0.43	5.86±0.22	5.26±0.35
**TM**	4.87±0.96	5.33±0.38	6.11±0.36	4.61±0.22
**NWC**	5.61±0.42	6.65±0.62	7.30±0.46	5.76±0.36
**Cu**	5.48±0.12	6.81±0.23	7.30±0.22	7.06±0.75
**Mol**	5.26±0.22	5.08±0.35	7.48±0.15	7.48±0.90
**t0.CO**	5.56±0.24	5.48±0.41	7.18±0.16	7.44±1.16
**t0.CR**	4.27±0.62	5.74±0.31	7.17±0.78	7.44±0.35
**t10.CO.A**	6.05±0.29	5.44±1.30	7.19±0.63	6.22±0.26
**t10.CR.A**	6.57±0.45	5.27±0.28	7.14±0.28	5.25±0.39
**t10.CO.B**	6.91±0.17	6.58±0.55	7.14±0.22	6.45±0.73
**t10.CR.B**	6.63±0.15	4.36±0.31	7.48±0.26	5.31±0.64
**t10.CO.C**	6.7±0.36	4.68±0.36	6.84±0.32	5.65±0.45
**t10.CR.C**	5.26±0.22	4.53±0.25	7.50±0.48	5.32±0.15
**t20.CO.A**	6.61±0.13	5.84±0.60	7.58±0.84	5.83±0.32
**t20.CR.A**	5.66±0.57	4.20±0.21	7.44±0.37	6.33±0.69
**t20.CO.B**	8.22±0.11	6.49±0.81	7.42±0.18	6.45±0.21
**t20.CR.B**	7.32±0.22	4.15±0.07	7.34±0.25	6.37±0.77
**t20.CO.C**	8.01±0.52	5.69±0.24	7.48±0.26	5.55±0.95
**t20.CR.C**	5.21±0.30	5.35±0.22	7.71±0.96	5.54±0.86
**t30.CO.A**	7.34±0.25	5.16±0.98	7.15±0.65	5.46±1.01
**t30.CR.A**	7.60±0.23	5.05±0.93	6.58±0.36	6.30±0.61
**t30.CO.B**	8.87±0.29	4.81±0.30	6.40±0.28	5.40±0.32
**t30.CR.B**	7.82±0.75	3.33±0.28	6.91±0.95	3.61±0.89
**t30.CO.C**	6.71±0.26	4.72±1.36	7.30±1.20	4.40±0.77
**t30.CR.C**	4.66±0.33	5.35±0.22	7.38±0.39	4.29±0.29

Values are reported as mean ± standard deviation (SD) of three determinations.

Table S4. KEGG genes differentially expressed* between cheese core samples ripened in standard condition and at higher temperature.

KEGG genes	log_2Fold Change	Adjusted P value
Environmentalinformationprocessing;Membranetransport;Ctransporters[PATH:ko02010];aapQ.bztB;generalL-aminoacidtransportsystempermeaseprotein	-5.79445058	0.021004259
Environmentalinformationprocessing;Membranetransport;Ctransporters[PATH:ko02010];ABC.FEV.S;ironcomplextransportsystemsubstrate-bindingprotein	-5.825832424	0.000379778
Environmentalinformationprocessing;Membranetransport;Ctransporters[PATH:ko02010];ABC.GLN1.A;putativeglutaminetransportsystemATP-bindingprotein[EC:3.6.3.-]	0.827662713	0.033036164
Environmentalinformationprocessing;Membranetransport;Ctransporters[PATH:ko02010];ABC.VB12.S1.btuF;vitaminB12transportsystemsubstrate-bindingprotein	-6.041019046	0.02302363
Environmentalinformationprocessing;Membranetransport;Ctransporters[PATH:ko02010];afuC.fbpC;iron(III)transportsystemATP-bindingprotein[EC:3.6.3.30]	-5.042539965	0.020748409
Environmentalinformationprocessing;Membranetransport;Ctransporters[PATH:ko02010];araF;L-arabinosetransportsystemsubstrate-bindingprotein	-4.699202506	0.043755579
Environmentalinformationprocessing;Membranetransport;Ctransporters[PATH:ko02010];artM;argininetransportsystempermeaseprotein	-5.162185444	0.04356273
Environmentalinformationprocessing;Membranetransport;Ctransporters[PATH:ko02010];bioY;biotintransportsystemsubstrate-specificcomponent	-1.557141374	0.017244007
Environmentalinformationprocessing;Membranetransport;Ctransporters[PATH:ko02010];dppB;dipeptidetransportsystempermeaseprotein	-6.360916658	3.08E-05
Environmentalinformationprocessing;Membranetransport;Ctransporters[PATH:ko02010];dppD;dipeptidetransportsystemATP-bindingprotein	-5.020346936	0.014271914
Environmentalinformationprocessing;Membranetransport;Ctransporters[PATH:ko02010];gspL;generalsecretionpathwayproteinL	-5.162975173	0.044283932
Environmentalinformationprocessing;Membranetransport;Ctransporters[PATH:ko02010];livM;branched-chainaminoacidtransportsystempermeaseprotein	-5.761707722	0.000730056
Environmentalinformationprocessing;Membranetransport;Ctransporters[PATH:ko02010];malE;maltose/maltodextrintransportsystemsubstrate-bindingprotein	-4.394973416	0.018152474
Environmentalinformationprocessing;Membranetransport;Ctransporters[PATH:ko02010];malF;maltose/maltodextrintransportsystempermeaseprotein	-4.078495417	0.042930947
Environmentalinformationprocessing;Membranetransport;Ctransporters[PATH:ko02010];malG;maltose/maltodextrintransportsystempermeaseprotein	-3.617921368	0.037642814
Environmentalinformationprocessing;Membranetransport;Ctransporters[PATH:ko02010];malK.mtlK.thuK;multiplesugartransportsystemATP-bindingprotein[EC:3.6.3.-]	-4.686140514	0.019589753
Environmentalinformationprocessing;Membranetransport;Ctransporters[PATH:ko02010];mglA;methyl-galactosidetransportsystemATP-bindingprotein[EC:3.6.3.17]	-5.520371977	0.040303703
Environmentalinformationprocessing;Membranetransport;Ctransporters[PATH:ko02010];mglB;methyl-galactosidetransportsystemsubstrate-bindingprotein	-5.802875091	0.029813011
Environmentalinformationprocessing;Membranetransport;Ctransporters[PATH:ko02010];msbA;ATP-bindingcassette.subfamilyB.bacterialMsbA[EC:3.6.3.-]	-6.223001057	0.005758234
Environmentalinformationprocessing;Membranetransport;Ctransporters[PATH:ko02010];msmX.msmK.malK.sugC.ggtA.msiK;multiplesugartransportsystemATP-bindingprotein	-5.08078914	0.000571588
Environmentalinformationprocessing;Membranetransport;Ctransporters[PATH:ko02010];nodI;lipooligosaccharidetransportsystemATP-bindingprotein	-5.508787084	0.002031792
Environmentalinformationprocessing;Membranetransport;Ctransporters[PATH:ko02010];nodJ;lipooligosaccharidetransportsystempermeaseprotein	-4.923850342	0.008424869

Environmentalinformationprocessing;Membranetransport;Ctransporters[PATH:ko02010];opuBD;osmoprotectanttransportsystempermeaseprotein	-3.826399874	0.012048496
Environmentalinformationprocessing;Membranetransport;Ctransporters[PATH:ko02010];opuC;osmoprotectanttransportsystemsubstrate-bindingprotein	-3.905138751	0.010739859
Environmentalinformationprocessing;Membranetransport;Ctransporters[PATH:ko02010];phnC;phosphonatetransportsystemATP-bindingprotein[EC:3.6.3.28]	-4.855635021	0.000308086
Environmentalinformationprocessing;Membranetransport;Ctransporters[PATH:ko02010];phnD;phosphonatetransportsystemsubstrate-bindingprotein	-5.147908585	3.13E-05
Environmentalinformationprocessing;Membranetransport;Ctransporters[PATH:ko02010];phnE;phosphonatetransportsystempermeaseprotein	-4.682026418	1.46E-05
Environmentalinformationprocessing;Membranetransport;Ctransporters[PATH:ko02010];proV;glycinebetaine/prolinetransportsystemATP-bindingprotein[EC:3.6.3.32]	-4.342908887	7.99E-06
Environmentalinformationprocessing;Membranetransport;Ctransporters[PATH:ko02010];proW;glycinebetaine/prolinetransportsystempermeasep rotein	-4.533502532	2.35E-05
Environmentalinformationprocessing;Membranetransport;Ctransporters[PATH:ko02010];proX;glycinebetaine/prolinetransportsystemsubstrate-bindingprotein	-4.50135696	2.04E-05
Environmentalinformationprocessing;Membranetransport;Ctransporters[PATH:ko02010];pstB;phosphatetransportsystemATP-bindingprotein[EC:3.6.3.27]	0.767093067	0.02975298
Environmentalinformationprocessing;Membranetransport;Ctransporters[PATH:ko02010];PTS-Aga-EIIA.agaF;PTSsystem.N-acetylgalactosamine-specificIIAcomponent[EC:2.7.1.69]	-4.846802103	3.93E-05
Environmentalinformationprocessing;Membranetransport;Ctransporters[PATH:ko02010];PTS-Bgl-EIIB.bglF;PTSsystem.beta-glucosides-specificIIBcomponent[EC:2.7.1.69]	-4.046032474	9.44E-05
Environmentalinformationprocessing;Membranetransport;Ctransporters[PATH:ko02010];PTS-Cel-EIIA.celC;PTSsystem.cellobiose-specificIIAcomponent[EC:2.7.1.69]	-5.152633964	7.50E-07
Environmentalinformationprocessing;Membranetransport;Ctransporters[PATH:ko02010];PTS-Cel-EIIB.celA;PTSsystem.cellobiose-specificIIBcomponent[EC:2.7.1.69]	-4.698040454	5.05E-06
Environmentalinformationprocessing;Membranetransport;Ctransporters[PATH:ko02010];PTS-Cel-EIIC.celB;PTSsystem.cellobiose-specificIICcomponent	-2.463007798	0.00032332
Environmentalinformationprocessing;Membranetransport;Ctransporters[PATH:ko02010];PTS-EI.PTSI.ptsI;phosphotransferasesystem.enzymeI.PtsI[EC:2.7.3.9]	-6.120948801	3.02E-05
Environmentalinformationprocessing;Membranetransport;Ctransporters[PATH:ko02010];PTS-Fru-EIIA.fruB;PTSsystem.fructose-specificIIAcomponent[EC:2.7.1.69]	-5.587499802	0.00039653
Environmentalinformationprocessing;Membranetransport;Ctransporters[PATH:ko02010];PTS-Fru-EIIB.fruA;PTSsystem.fructose-specificIIBcomponent[EC:2.7.1.69]	-5.588803896	0.00039653
Environmentalinformationprocessing;Membranetransport;Ctransporters[PATH:ko02010];PTS-Fru-EIIC.fruA;PTSsystem.fructose-specificIICcomponent	-5.588803891	0.00039653
Environmentalinformationprocessing;Membranetransport;Ctransporters[PATH:ko02010];PTS-Gat-EIIC.gatC;PTSsystem.galactitol-specificIICcomponent	-3.263125977	0.018152474
Environmentalinformationprocessing;Membranetransport;Ctransporters[PATH:ko02010];PTS-Man-EIID.manZ;PTSsystem.mannose-specificIIDcomponent	1.688426934	0.029533322
Environmentalinformationprocessing;Membranetransport;Ctransporters[PATH:ko02010];PTS-Ula-EIIA.ulaC.sgaA;PTSsystem.ascorbate-specificIIAcomponent[EC:2.7.1.69]	-5.441430331	1.37E-05
Environmentalinformationprocessing;Membranetransport;Ctransporters[PATH:ko02010];PTS-Ula-EIIB.ulaB.sgaB;PTSsystem.ascorbate-specificIIBcomponent[EC:2.7.1.69]	-4.012856339	0.000231184
Environmentalinformationprocessing;Membranetransport;Ctransporters[PATH:ko02010];PTS-Ula-EIIC.ulaA.sgaT;PTSsystem.ascorbate-specificIICcomponent	-5.228916806	5.96E-06

Environmentalinformationprocessing;Membranetransport;Ctransporters[PATH:ko02010];rbsA;ribosetransportsystemATP-bindingprotein[EC:3.6.3.17]	-4.812403961	9.48E-05
Environmentalinformationprocessing;Membranetransport;Ctransporters[PATH:ko02010];rbsB;ribosetransportsystemsubstrate-bindingprotein	-5.604951479	2.42E-07
Environmentalinformationprocessing;Membranetransport;Ctransporters[PATH:ko02010];rbsC;ribosetransportsystempermeaseprotein	-5.017029039	1.30E-05
Environmentalinformationprocessing;Membranetransport;Ctransporters[PATH:ko02010];rbsD;D-ribosepyranase[EC:5.-.-.-]	-4.680373806	0.000234784
Environmentalinformationprocessing;Membranetransport;Ctransporters[PATH:ko02010];secE;preproteintranslocasesubunitSecE	1.14660634	0.012808524
Environmentalinformationprocessing;Membranetransport;Ctransporters[PATH:ko02010];secF;preproteintranslocasesubunitSecF	-4.676083612	0.032725985
Environmentalinformationprocessing;Membranetransport;Ctransporters[PATH:ko02010];tagG;teichoicacidtransportsystempermeaseprotein	-5.653678072	3.55E-07
Environmentalinformationprocessing;Membranetransport;Ctransporters[PATH:ko02010];tagH;teichoicacidtransportsystemATP-bindingprotein[EC:3.6.3.40]	-4.624431029	1.55E-05
Environmentalinformationprocessing;Membranetransport;Ctransporters[PATH:ko02010];ugpA;sn-glycerol3-phosphatetransportsystempermeaseprotein	-5.240890087	2.53E-06
Environmentalinformationprocessing;Membranetransport;Ctransporters[PATH:ko02010];ugpB;sn-glycerol3-phosphatetransportsystemsubstrate-bindingprotein	-4.806639566	3.71E-07
Environmentalinformationprocessing;Membranetransport;Ctransporters[PATH:ko02010];ugpC;sn-glycerol3-phosphatetransportsystemATP-bindingprotein[EC:3.6.3.20]	-5.28324773	5.02E-05
Environmentalinformationprocessing;Membranetransport;Ctransporters[PATH:ko02010];ugpE;sn-glycerol3-phosphatetransportsystempermeaseprotein	-4.922403666	1.17E-05
Environmentalinformationprocessing;Membranetransport;Ctransporters[PATH:ko02010];virD4.lvhD4;typeIVsecretionsystemproteinVirD4	-5.371159934	0.007319264
Environmentalinformationprocessing;Membranetransport;Ctransporters[PATH:ko02010];xylG;D-xylosetransportsystemATP-bindingprotein[EC:3.6.3.17]	-5.134973505	0.02302363
Environmentalinformationprocessing;Membranetransport;Ctransporters[PATH:ko02010];yojI;putativeATP-bindingcassettetransporter	-5.794042408	0.022225319
Environmentalinformationprocessing;Signaltrasduction;HIF-1signalingpathway[PATH:ko04066];ENO.eno;enolase[EC:4.2.1.11]	-5.950546634	6.12E-05
Environmentalinformationprocessing;Signaltrasduction;HIF-1signalingpathway[PATH:ko04066];GAPDH.gapA;glyceraldehyde3-phosphatedehydrogenase[EC:1.2.1.12]	-5.458656315	0.000134338
Environmentalinformationprocessing;Signaltrasduction;PI3K-Aktsignalingpathway[PATH:ko04151];htpG.HSP90A;molecularchaperoneHtpG	-5.070633139	0.037642814
Environmentalinformationprocessing;Signaltrasduction;Two-componentsystem[PATH:ko02020];agrA;two-componentsystem.AgrAfamily.responseregulatorAgrA	-3.436225515	0.000243456
Environmentalinformationprocessing;Signaltrasduction;Two-componentsystem[PATH:ko02020];agrC;two-componentsystem.AgrAfamily.sensorhistidinekinaseAgrC[EC:2.7.13.-]	-3.668420447	2.98E-05
Environmentalinformationprocessing;Signaltrasduction;Two-componentsystem[PATH:ko02020];atoE;short-chainfattyacidtransporter	-5.163257353	0.043141763
Environmentalinformationprocessing;Signaltrasduction;Two-componentsystem[PATH:ko02020];cheR;chemotaxisproteinmethyltransferaseCheR[EC:2.1.1.80]	-6.032709365	0.016092491
Environmentalinformationprocessing;Signaltrasduction;Two-componentsystem[PATH:ko02020];ciaH;two-componentsystem.OmpRfamily.sensorhistidinekinaseCiaH[EC:2.7.13.3]	-5.148019687	7.83E-05
Environmentalinformationprocessing;Signaltrasduction;Two-componentsystem[PATH:ko02020];ciaR;two-componentsystem.OmpRfamily.responseregulatorCiaR	-3.678644298	3.62E-06
Environmentalinformationprocessing;Signaltrasduction;Two-componentsystem[PATH:ko02020];citC;[citrate(pro-3S)-lyase]ligase[EC:6.2.1.22]	-6.306858006	2.32E-08

Environmentalinformationprocessing;Signaltrasduction;Two-componentsystem[PATH:ko02020];citD;citratelyasesubunitgamma(acyl carrierprotein)	-5.161523661	7.19E-05
Environmentalinformationprocessing;Signaltrasduction;Two-componentsystem[PATH:ko02020];citE;citratelyasesubunitbeta/citryl-CoAlyase[EC:4.1.3.34]	-5.705675918	2.75E-07
Environmentalinformationprocessing;Signaltrasduction;Two-componentsystem[PATH:ko02020];citF;citratelyasesubunitalpha/citrate CoA-transferase[EC:2.8.3.10]	-6.662554821	9.13E-08
Environmentalinformationprocessing;Signaltrasduction;Two-componentsystem[PATH:ko02020];citXG;holo-ACPsynthase/triphosphoribosyl-dephospho-CoAsynthase[EC:2.7.7.612.4.2.52]	-6.569294057	2.23E-08
Environmentalinformationprocessing;Signaltrasduction;Two-componentsystem[PATH:ko02020];cusA.silA;Cu(I)/Ag(I)effluxsystem membraneproteinCusA/SilA	-5.136698074	0.024592108
Environmentalinformationprocessing;Signaltrasduction;Two-componentsystem[PATH:ko02020];cydA;cytochromedubiquinoloxidase subunitI[EC:1.10.3.-]	-5.064875327	1.51E-05
Environmentalinformationprocessing;Signaltrasduction;Two-componentsystem[PATH:ko02020];cydB;cytochromedubiquinoloxidase subunitII[EC:1.10.3.-]	-5.137486528	1.48E-06
Environmentalinformationprocessing;Signaltrasduction;Two-componentsystem[PATH:ko02020];degP.htrA;serineproteaseDo[EC:3.4.21.107]	-6.579035273	1.53E-05
Environmentalinformationprocessing;Signaltrasduction;Two-componentsystem[PATH:ko02020];E2.3.1.9.atoB;acetyl-CoAC-acetyltransferase[EC:2.3.1.9]	-3.373422428	0.00346328
Environmentalinformationprocessing;Signaltrasduction;Two-componentsystem[PATH:ko02020];frdA;fumaratereductaseflavoproteinsubunit[EC:1.3.5.4]	-3.39479139	0.012913123
Environmentalinformationprocessing;Signaltrasduction;Two-componentsystem[PATH:ko02020];kdpA;K+-transportingATPaseATPaseAchain[EC:3.6.3.12]	-7.220993578	0.004572006
Environmentalinformationprocessing;Signaltrasduction;Two-componentsystem[PATH:ko02020];lrgA;holin-likeprotein	-4.837480365	0.000430229
Environmentalinformationprocessing;Signaltrasduction;Two-componentsystem[PATH:ko02020];lrgB;holin-likeproteinLrgB	-6.39764061	0.000312268
Environmentalinformationprocessing;Signaltrasduction;Two-componentsystem[PATH:ko02020];malR;two-componentsystem.CitBfamily.responseregulatorMalR	-5.823963576	0.001678448
Environmentalinformationprocessing;Signaltrasduction;Two-componentsystem[PATH:ko02020];mcp;methyl-acceptingchemotaxisprotein	-4.13974575	0.043106084
Environmentalinformationprocessing;Signaltrasduction;Two-componentsystem[PATH:ko02020];mdtC;RNDsuperfamily.multidrugtransportproteinMdtC	-12.23880014	0.004523313
Environmentalinformationprocessing;Signaltrasduction;Two-componentsystem[PATH:ko02020];narX;two-componentsystem.NarLfamily.nitrate/nitritesensorhistidinekinaseNarX[EC:2.7.13.3]	-11.80991106	0.016700344
Environmentalinformationprocessing;Signaltrasduction;Two-componentsystem[PATH:ko02020];phoB1.phoP;two-componentsystem.OmpRfamily.alkalinephosphatasesynthesisresponseregulatorPhoP	-6.000304319	2.31E-07
Environmentalinformationprocessing;Signaltrasduction;Two-componentsystem[PATH:ko02020];phoR;two-componentsystem.OmpRfamily.phosphateregulonsensorhistidinekinasePhoR[EC:2.7.13.3]	-5.97650818	2.36E-08
Environmentalinformationprocessing;Signaltrasduction;Two-componentsystem[PATH:ko02020];rstB;two-componentsystem.OmpRfamily.sensorhistidinekinaseRstB[EC:2.7.13.3]	-8.54976537	0.005903541
Environmentalinformationprocessing;Signaltrasduction;Two-componentsystem[PATH:ko02020];trg;methyl-acceptingchemotaxisproteinIII.riboseandgalactosesensorreceptor	-13.0990089	0.001572765

Environmentalinformationprocessing;Signaltrasduction;Two-componentsystem[PATH:ko02020];uhpC;MFStransporter.OPAfamily.sugarphosphatesensorproteinUhpC	-11.76854005	0.002995451
Environmentalinformationprocessing;Signaltrasduction;Two-componentsystem[PATH:ko02020];uhpT;MFStransporter.OPAfamily.hexosephosphatetransportproteinUhpT	-13.30306937	0.001164129
Environmentalinformationprocessing;Signaltrasduction;Two-componentsystem[PATH:ko02020];vraR;two-componentsystem.NarLfamily.vancomycinresistanceassociatedresponseregulatorVraR	-8.025730609	0.01063941
Environmentalinformationprocessing;Signaltrasduction;Two-componentsystem[PATH:ko02020];yufL.malK;two-componentsystem.CitBfamily.sensorhistidinekinaseMalK[EC:2.7.13.3]	-5.484163521	0.048249327
GeneticinformationandprocessingTranscription;Aminoacyl-tRNAbiosynthesis[PATH:ko00970];EARS.gltX;glutamyl-tRNAsynthetase[EC:6.1.1.17]	-8.769486459	0.039283267
GeneticinformationandprocessingTranscription;Aminoacyl-tRNAbiosynthesis[PATH:ko00970];gltX;nondiscriminatingglutamyl-tRNAsynthetase[EC:6.1.1.24]	-1.073337721	0.047440494
GeneticinformationandprocessingTranscription;Aminoacyl-tRNAbiosynthesis[PATH:ko00970];glyQ;glycyl-tRNAsynthetasealphachain[EC:6.1.1.14]	0.973594969	0.011586317
GeneticinformationandprocessingTranscription;Aminoacyl-tRNAbiosynthesis[PATH:ko00970];glyS;glycyl-tRNAsynthetasebetachain[EC:6.1.1.14]	-6.116192416	8.78E-05
GeneticinformationandprocessingTranscription;Aminoacyl-tRNAbiosynthesis[PATH:ko00970];MTFMT.fmt;methionyl-tRNAformyltransferase[EC:2.1.2.9]	0.89200961	0.002995451
GeneticinformationandprocessingTranscription;Aminoacyl-tRNAbiosynthesis[PATH:ko00970];QARS.glnS;glutaminyl-tRNAsynthetase[EC:6.1.1.18]	-11.75388851	0.001912743
GeneticinformationandprocessingTranscription;Aminoacyl-tRNAbiosynthesis[PATH:ko00970];RARS.argS;arginyl-tRNAsynthetase[EC:6.1.1.19]	0.727250117	0.004349097
GeneticinformationandprocessingTranscription;Aminoacyl-tRNAbiosynthesis[PATH:ko00970];WARS.trpS;tryptophanyl-tRNAsynthetase[EC:6.1.1.2]	-1.687480796	0.00540414
GeneticinformationandprocessingTranscription;Aminoacyl-tRNAbiosynthesis[PATH:ko00970];YARS.tyrS;tyrosyl-tRNAsynthetase[EC:6.1.1.1]	-3.021644096	0.005746486
GeneticInformationProcessing;Folding.sortinganddegradation;RNAdegradation[PATH:ko03018];dnaK;molecularchaperoneDnaK	-8.078495426	2.34E-08
GeneticInformationProcessing;Folding.sortinganddegradation;RNAdegradation[PATH:ko03018];ENO.eno;enolase[EC:4.2.1.11]	-5.951624563	6.12E-05
GeneticInformationProcessing;Folding.sortinganddegradation;RNAdegradation[PATH:ko03018];groEL.HSPD1;chaperoninGroEL	-9.178187903	1.46E-09
GeneticInformationProcessing;Folding.sortinganddegradation;RNAdegradation[PATH:ko03018];ppk;polyphosphatekinase[EC:2.7.4.1]	-5.057087872	2.54E-05
GeneticInformationProcessing;Folding.sortinganddegradation;RNAdegradation[PATH:ko03018];recQ;ATP-dependentDNAhelicaseRecQ[EC:3.6.4.12]	-6.427476407	1.42E-08
GeneticInformationProcessing;Folding.sortinganddegradation;RNAdegradation[PATH:ko03018];rhlB;ATP-dependentRNAhelicaseRhlB[EC:3.6.4.13]	-12.23457804	0.00346328
GeneticInformationProcessing;Folding.sortinganddegradation;RNAdegradation[PATH:ko03018];rho;Transcription;terminationfactorRho	-11.8085478	0.015753168
GeneticInformationProcessing;Folding.sortinganddegradation;RNAdegradation[PATH:ko03018];rnj;ribonucleaseJ[EC:3.1.-.-]	0.836349493	0.011983039
GeneticInformationProcessing;Folding.sortinganddegradation;Sulfurrelaysystem[PATH:ko04122];tusE.dsrC;tRNA2-thiouridinesynthesizingproteinE[EC:2.8.1.-]	-12.24908257	0.007708466
GeneticInformationProcessing;ReplicationandRepair;Baseexcisionrepair[PATH:ko03410];UNG.UDG;uracil-DNAglycosylase[EC:3.2.2.27]	-2.156528146	0.002100338
GeneticInformationProcessing;ReplicationandRepair;DNAreplication[PATH:ko03030];DPO3D1.holA;DNApolymeraseIIIsubunitdelta[EC:2.7.7.7]	-2.057134442	0.001769558

GeneticInformationProcessing;ReplicationandRepair;DNAreplication[PATH:ko03030];DPO3E.dnaQ;DNApolymeraseIIIsubunitepsilon[EC:2.7.7.7]	-2.281934658	0.014968485
GeneticInformationProcessing;ReplicationandRepair;DNAreplication[PATH:ko03030];E3.1.26.4A.RNASEH1.rnhA;ribonucleaseHI[EC:3.1.26.4]	-6.887346204	2.76E-07
GeneticInformationProcessing;ReplicationandRepair;Homologousrecombination[PATH:ko03440];DPO3D1.holA;DNApolymeraseIIIsubunitdelta[EC:2.7.7.7]	-2.057134442	0.001769558
GeneticInformationProcessing;ReplicationandRepair;Homologousrecombination[PATH:ko03440];DPO3E.dnaQ;DNApolymeraseIIIsubunitepsilon[EC:2.7.7.7]	-2.281934658	0.014968485
GeneticInformationProcessing;ReplicationandRepair;Homologousrecombination[PATH:ko03440];recA;recombinationproteinRecA	0.836567563	0.0271357
GeneticInformationProcessing;ReplicationandRepair;Homologousrecombination[PATH:ko03440];recB;exodeoxyribonucleaseVbetasubunit[EC:3.1.11.5]	-11.77951562	0.004389403
GeneticInformationProcessing;ReplicationandRepair;Homologousrecombination[PATH:ko03440];recC;exodeoxyribonucleaseVgammasubunit[EC:3.1.11.5]	-13.29246399	0.000592897
GeneticInformationProcessing;ReplicationandRepair;Homologousrecombination[PATH:ko03440];recF;DNAReplicationandRepair;proteinRecF	-1.245840594	0.034583942
GeneticInformationProcessing;ReplicationandRepair;Homologousrecombination[PATH:ko03440];recO;DNArepairproteinRecO(recombinationproteinO)	-3.194746372	0.000124049
GeneticInformationProcessing;ReplicationandRepair;Homologousrecombination[PATH:ko03440];recR;recombinationproteinRecR	-2.826461128	0.000868497
GeneticInformationProcessing;ReplicationandRepair;Homologousrecombination[PATH:ko03440];ruvB;hollidayjunctionDNAhelicaseRuvB[EC:3.6.4.12]	1.152675189	0.004963488
GeneticInformationProcessing;ReplicationandRepair;Mismatchrepair[PATH:ko03430];dam;DNAadeninemethylase[EC:2.1.1.72]	-5.547933664	0.001180211
GeneticInformationProcessing;ReplicationandRepair;Mismatchrepair[PATH:ko03430];DPO3D1.holA;DNApolymeraseIIIsubunitdelta[EC:2.7.7.7]	-2.057134442	0.001769558
GeneticInformationProcessing;ReplicationandRepair;Mismatchrepair[PATH:ko03430];DPO3E.dnaQ;DNApolymeraseIIIsubunitepsilon[EC:2.7.7.7]	-2.281934658	0.014968485
GeneticInformationProcessing;ReplicationandRepair;Mismatchrepair[PATH:ko03430];uvrD.pcrA;DNAhelicaseII/ATP-dependentDNAhelicasePcrA[EC:3.6.4.12]	-2.393805795	0.023107112
GeneticInformationProcessing;ReplicationandRepair;Nucleotideexcisionrepair[PATH:ko03420];uvrD.pcrA;DNAhelicaseII/ATP-dependentDNAhelicasePcrA[EC:3.6.4.12]	-2.393805795	0.023107112
GeneticInformationProcessing;Transcription;Ribosome[PATH:ko03010];RP-L20.MRPL20.rplT;largesubunitribosomalproteinL20	-2.496163215	0.007765704
GeneticInformationProcessing;Transcription;Ribosome[PATH:ko03010];RP-L24.MRPL24.rplX;largesubunitribosomalproteinL24	1.468966868	0.005713358
GeneticInformationProcessing;Transcription;Ribosome[PATH:ko03010];RP-L27.MRPL27.rpmA;largesubunitribosomalproteinL27	1.156896883	0.040207839
GeneticInformationProcessing;Transcription;Ribosome[PATH:ko03010];RP-L28.MRPL28.rpmB;largesubunitribosomalproteinL28	-2.253513018	0.012913123
GeneticInformationProcessing;Transcription;Ribosome[PATH:ko03010];RP-L34.MRPL34.rpmH;largesubunitribosomalproteinL34	-4.33611587	0.00057019
GeneticInformationProcessing;Transcription;Ribosome[PATH:ko03010];RP-L35.MRPL35.rpmI;largesubunitribosomalproteinL35	-1.924366964	0.043979192
GeneticInformationProcessing;Transcription;Ribosome[PATH:ko03010];RP-L36.MRPL36.rpmJ;largesubunitribosomalproteinL36	-2.800870899	0.00874987
GeneticInformationProcessing;Transcription;Ribosome[PATH:ko03010];RP-S10.MRPS10.rpsJ;smallsubunitribosomalproteinS10	-2.954335511	6.24E-05
GeneticInformationProcessing;Transcription;Ribosome[PATH:ko03010];RP-S13.rpsM;smallsubunitribosomalproteinS13	-1.519798236	0.033036164
GeneticInformationProcessing;Transcription;Ribosome[PATH:ko03010];RP-S18.MRPS18.rpsR;smallsubunitribosomalproteinS18	-2.63168196	0.003201241
GeneticInformationProcessing;Transcription;Ribosome[PATH:ko03010];RP-S20.rpsT;smallsubunitribosomalproteinS20	1.226072852	0.020069631

GeneticInformationProcessing;Transcription;Ribosome[PATH:ko03010];RP-S3.rpsC;smallsubunitribosomalproteinS3	1.272642324	0.007708466
GeneticInformationProcessing;Transcription;Ribosome[PATH:ko03010];RP-S4.rpsD;smallsubunitribosomalproteinS4	-1.822719232	0.016700344
GeneticInformationProcessing;Transcription;Ribosome[PATH:ko03010];RP-S6.MRPS6.rpsF;smallsubunitribosomalproteinS6	-2.771933346	0.00346328
GeneticInformationProcessing;Transcription;RNApolymerase[PATH:ko03020];rpoZ;DNA-directedRNApolymerasesubunitomega[EC:2.7.7.6]	1.140001556	0.003577006
Metabolism;Aminoacidmetabolism;Alanine.aspartateandglutamatemetabolism[PATH:ko00250];ald;alaninedehydrogenase[EC:1.4.1.1]	-5.521648089	0.009264107
Metabolism;Aminoacidmetabolism;Alanine.aspartateandglutamatemetabolism[PATH:ko00250];argG.ASS1;argininosuccinatesynthase[EC:6.3.4.5]	-2.488905339	0.001164129
Metabolism;Aminoacidmetabolism;Alanine.aspartateandglutamatemetabolism[PATH:ko00250];argH.ASL;argininosuccinatelyase[EC:4.3.2.1]	-3.714692095	1.59E-05
Metabolism;Aminoacidmetabolism;Alanine.aspartateandglutamatemetabolism[PATH:ko00250];aspA;aspartateammonia-lyase[EC:4.3.1.1]	-6.313900145	2.95E-06
Metabolism;Aminoacidmetabolism;Alanine.aspartateandglutamatemetabolism[PATH:ko00250];carA.CPA1;carbamoyl-phosphatesynthasesmallsubunit[EC:6.3.5.5]	-6.395428566	6.52E-05
Metabolism;Aminoacidmetabolism;Alanine.aspartateandglutamatemetabolism[PATH:ko00250];E5.1.1.13;aspartateracemase[EC:5.1.1.13]	-4.942524921	0.012913123
Metabolism;Aminoacidmetabolism;Alanine.aspartateandglutamatemetabolism[PATH:ko00250];gabD;succinate-semialdehydedehydrogenase/glutarate-semialdehydedehydrogenase[EC:1.2.1.161.2.1.791.2.1.20]	-4.549986885	1.37E-05
Metabolism;Aminoacidmetabolism;Alanine.aspartateandglutamatemetabolism[PATH:ko00250];gltB;glutamatesynthase(NADPH/NADH)largechain[EC:1.4.1.131.4.1.14]	-7.841446025	0.000132326
Metabolism;Aminoacidmetabolism;Alanine.aspartateandglutamatemetabolism[PATH:ko00250];gltD;glutamatesynthase(NADPH/NADH)smallchain[EC:1.4.1.131.4.1.14]	-12.19765424	0.00100271
Metabolism;Aminoacidmetabolism;Alanine.aspartateandglutamatemetabolism[PATH:ko00250];purB.ADSL;adenylosuccinatelyase[EC:4.3.2.2]	1.408979944	0.000660017
Metabolism;Aminoacidmetabolism;Alanine.aspartateandglutamatemetabolism[PATH:ko00250];putA;prolinedehydrogenase/delta1-pyrroline-5-carboxylatedehydrogenase[EC:1.5.5.21.2.1.88]	-13.47998144	0.000831604
Metabolism;Aminoacidmetabolism;Alanine.aspartateandglutamatemetabolism[PATH:ko00250];puuE;4-aminobutyrateaminotransferase[EC:2.6.1.19]	-5.839008597	1.17E-05
Metabolism;Aminoacidmetabolism;Alanine.aspartateandglutamatemetabolism[PATH:ko00250];pyrB.PYR2;aspartatecarbamoyltransferasecatalyticsubunit[EC:2.1.3.2]	0.766457577	0.047424644
Metabolism;Aminoacidmetabolism;Arginineandprolinemetabolism[PATH:ko00330];arcC;carbamatekinase[EC:2.7.2.2]	-11.15287326	0.003472935
Metabolism;Aminoacidmetabolism;Arginineandprolinemetabolism[PATH:ko00330];argC;N-acetyl-gamma-glutamyl-phosphatereductase[EC:1.2.1.38]	-3.656685122	0.004196998
Metabolism;Aminoacidmetabolism;Arginineandprolinemetabolism[PATH:ko00330];argD;acetylornithine/N-succinyldiaminopimelateaminotransferase[EC:2.6.1.112.6.1.17]	-11.79756423	0.009303631
Metabolism;Aminoacidmetabolism;Arginineandprolinemetabolism[PATH:ko00330];argG.ASS1;argininosuccinatesynthase[EC:6.3.4.5]	-2.488905339	0.001164129
Metabolism;Aminoacidmetabolism;Arginineandprolinemetabolism[PATH:ko00330];argH.ASL;argininosuccinatelyase[EC:4.3.2.1]	-3.714692095	1.59E-05
Metabolism;Aminoacidmetabolism;Arginineandprolinemetabolism[PATH:ko00330];astC;succinylornithineaminotransferase[EC:2.6.1.81]	-12.25415839	0.010072111
Metabolism;Aminoacidmetabolism;Arginineandprolinemetabolism[PATH:ko00330];codA;cytosinedeaminase[EC:3.5.4.1]	-3.331975975	0.02302363
Metabolism;Aminoacidmetabolism;Arginineandprolinemetabolism[PATH:ko00330];E1.5.1.2.proC;pyrroline-5-carboxylatereductase[EC:1.5.1.2]	-1.183816916	0.024547842
Metabolism;Aminoacidmetabolism;Arginineandprolinemetabolism[PATH:ko00330];E2.3.1.57.speG;diamineN-acetyltransferase[EC:2.3.1.57]	-6.028212858	2.42E-07
Metabolism;Aminoacidmetabolism;Arginineandprolinemetabolism[PATH:ko00330];E3.5.1.4.amiE;amidase[EC:3.5.1.4]	-9.159795129	0.012495682
Metabolism;Aminoacidmetabolism;Arginineandprolinemetabolism[PATH:ko00330];E3.5.3.6.arcA;argininedeiminase[EC:3.5.3.6]	-7.950039654	0.000119513

Metabolism;Aminoacidmetabolism;Arginineandprolinemetabolism[PATH:ko00330];E4.1.1.17.ODC1.speC.speF;ornithinedecarboxylase[EC:4.1.1.17]	-9.436250667	0.002995451
Metabolism;Aminoacidmetabolism;Arginineandprolinemetabolism[PATH:ko00330];OTC.argF.argI;ornithinecarbamoyltransferase[EC:2.1.3.3]	-2.288340744	0.002121995
Metabolism;Aminoacidmetabolism;Arginineandprolinemetabolism[PATH:ko00330];prr;aminobutyraldehydedehydrogenase[EC:1.2.1.19]	-11.81146043	0.018385998
Metabolism;Aminoacidmetabolism;Arginineandprolinemetabolism[PATH:ko00330];putA;prolinedehydrogenase/delta1-pyrroline-5-carboxylatedehydrogenase[EC:1.5.5.21.2.1.88]	-13.47998144	0.000831604
Metabolism;Aminoacidmetabolism;Cysteineandmethioninemetabolism[PATH:ko00270];E1.1.1.3;homoserinedehydrogenase[EC:1.1.1.3]	-1.457338016	0.02429635
Metabolism;Aminoacidmetabolism;Cysteineandmethioninemetabolism[PATH:ko00270];LDH.ldh;L-lactatedehydrogenase[EC:1.1.1.27]	1.073131866	0.038702186
Metabolism;Aminoacidmetabolism;Cysteineandmethioninemetabolism[PATH:ko00270];lysC;aspartatekinase[EC:2.7.2.4]	-2.470426967	0.000868497
Metabolism;Aminoacidmetabolism;Cysteineandmethioninemetabolism[PATH:ko00270];metC;cystathioninebeta-lyase[EC:4.4.1.8]	-5.996518004	0.000275868
Metabolism;Aminoacidmetabolism;Cysteineandmethioninemetabolism[PATH:ko00270];mmuM;homocysteineS-methyltransferase[EC:2.1.1.10]	-2.741641883	0.000767427
Metabolism;Aminoacidmetabolism;Cysteineandmethioninemetabolism[PATH:ko00270];thrA;bifunctionalaspartokinase/homoserinedehydrogenase1[EC:2.7.2.41.1.1.3]	-13.32943573	0.014525754
Metabolism;Aminoacidmetabolism;Glycine.serineandthreoninemetabolism[PATH:ko00260];DLD.lpd.pdhD;dihydrolipoamidedehydrogenase[EC:1.8.1.4]	-6.375720181	2.61E-05
Metabolism;Aminoacidmetabolism;Glycine.serineandthreoninemetabolism[PATH:ko00260];E1.1.1.3;homoserinedehydrogenase[EC:1.1.1.3]	-1.457338016	0.02429635
Metabolism;Aminoacidmetabolism;Glycine.serineandthreoninemetabolism[PATH:ko00260];gcvH.GCSH;glycinecleavagesystemHprotein	-7.572877698	0.003467562
Metabolism;Aminoacidmetabolism;Glycine.serineandthreoninemetabolism[PATH:ko00260];gcvT.AMT;aminomethyltransferase[EC:2.1.2.10]	-12.21984444	0.002190529
Metabolism;Aminoacidmetabolism;Glycine.serineandthreoninemetabolism[PATH:ko00260];GLDC.gcvP;glycinedehydrogenase[EC:1.4.4.2]	-8.292996831	0.024593612
Metabolism;Aminoacidmetabolism;Glycine.serineandthreoninemetabolism[PATH:ko00260];glyA.SHMT;glycinehydroxymethyltransferase[EC:2.1.2.1]	1.57364268	6.61E-07
Metabolism;Aminoacidmetabolism;Glycine.serineandthreoninemetabolism[PATH:ko00260];lysC;aspartatekinase[EC:2.7.2.4]	-2.470426967	0.000868497
Metabolism;Aminoacidmetabolism;Glycine.serineandthreoninemetabolism[PATH:ko00260];serA.PHGDH;D-3-phosphoglyceratedehydrogenase[EC:1.1.1.95]	-3.36727392	5.99E-05
Metabolism;Aminoacidmetabolism;Glycine.serineandthreoninemetabolism[PATH:ko00260];serC.PSAT1;phosphoserineaminotransferase[EC:2.6.1.52]	-2.871256379	0.000521046
Metabolism;Aminoacidmetabolism;Glycine.serineandthreoninemetabolism[PATH:ko00260];tdh;threonine3-dehydrogenase[EC:1.1.1.103]	-10.01944238	0.005129919
Metabolism;Aminoacidmetabolism;Glycine.serineandthreoninemetabolism[PATH:ko00260];thrA;bifunctionalaspartokinase/homoserinedehydrogenase1[EC:2.7.2.41.1.1.3]	-13.32943573	0.014525754
Metabolism;Aminoacidmetabolism;Glycine.serineandthreoninemetabolism[PATH:ko00260];thrB1;homoserinekinase[EC:2.7.1.39]	-1.925287818	0.00346328
Metabolism;Aminoacidmetabolism;Histidinemetabolism[PATH:ko00340];E3.1.3.15B;histidinol-phosphatase(PHPfamily)[EC:3.1.3.15]	-7.431575909	1.14E-08
Metabolism;Aminoacidmetabolism;Histidinemetabolism[PATH:ko00340];hisA;phosphoribosylformimino-5-aminoimidazolecarboxamideribotideisomerase[EC:5.3.1.16]	-1.487524168	0.046606821
Metabolism;Aminoacidmetabolism;Histidinemetabolism[PATH:ko00340];hisB;imidazoleglycerol-phosphatedehydratase[EC:4.2.1.19]	-2.975566053	0.000500234
Metabolism;Aminoacidmetabolism;Histidinemetabolism[PATH:ko00340];hisC;histidinol-phosphateaminotransferase[EC:2.6.1.9]	-4.325886491	1.99E-06
Metabolism;Aminoacidmetabolism;Histidinemetabolism[PATH:ko00340];hisD;histidinoldehydrogenase[EC:1.1.1.23]	-2.518577829	0.001771241
Metabolism;Aminoacidmetabolism;Histidinemetabolism[PATH:ko00340];hisE;phosphoribosyl-ATPpyrophosphohydrolase[EC:3.6.1.31]	-2.744707468	0.001947491
Metabolism;Aminoacidmetabolism;Histidinemetabolism[PATH:ko00340];hisF;cyclase[EC:4.1.3.-]	-2.060923175	0.010567335

Metabolism;Aminoacidmetabolism;Histidinemetabolism[PATH:ko00340];hisH;glutamineamidotransferase[EC:2.4.2.-]	-2.214467993	0.004442549
Metabolism;Aminoacidmetabolism;Histidinemetabolism[PATH:ko00340];hisI;phosphoribosyl-AMPcyclohydrolase[EC:3.5.4.19]	-2.184568523	0.008514636
Metabolism;Aminoacidmetabolism;Lysinebiosynthesis[PATH:ko00300];argD;acetylornithine/N-succinyldiaminopimelateaminotransferase[EC:2.6.1.112.6.1.17]	-11.79756423	0.009303631
Metabolism;Aminoacidmetabolism;Lysinebiosynthesis[PATH:ko00300];dapA;4-hydroxy-tetrahydrodipicolinatesynthase[EC:4.3.3.7]	-1.322063751	0.038521461
Metabolism;Aminoacidmetabolism;Lysinebiosynthesis[PATH:ko00300];dapB;4-hydroxy-tetrahydrodipicolinatereductase[EC:1.17.1.8]	1.3854821	2.54E-05
Metabolism;Aminoacidmetabolism;Lysinebiosynthesis[PATH:ko00300];dapC;N-succinyldiaminopimelateaminotransferase[EC:2.6.1.17]	-11.141225	0.001653568
Metabolism;Aminoacidmetabolism;Lysinebiosynthesis[PATH:ko00300];E1.1.1.3;homoserinedehydrogenase[EC:1.1.1.3]	-1.457338016	0.02429635
Metabolism;Aminoacidmetabolism;Lysinebiosynthesis[PATH:ko00300];lysC;aspartatekinase[EC:2.7.2.4]	-2.470426967	0.000868497
Metabolism;Aminoacidmetabolism;Lysinebiosynthesis[PATH:ko00300];murE;UDP-N-acetylmuramoyl-L-alanyl-D-glutamate--2.6-diaminopimelateligase[EC:6.3.2.13]	-1.319193837	0.035849171
Metabolism;Aminoacidmetabolism;Lysinebiosynthesis[PATH:ko00300];patA;aminotransferase[EC:2.6.1.-]	-3.048929303	3.84E-05
Metabolism;Aminoacidmetabolism;Lysinebiosynthesis[PATH:ko00300];thrA;bifunctionalaspartokinase/homoserinedehydrogenase1[EC:2.7.2.41.1.1.3]	-13.32943573	0.014525754
Metabolism;Aminoacidmetabolism;Lysinedegradation[PATH:ko00310];E2.3.1.9.atoB;acetyl-CoAC-acetyltransferase[EC:2.3.1.9]	-3.373422428	0.00346328
Metabolism;Aminoacidmetabolism;Lysinedegradation[PATH:ko00310];E4.1.1.18.ldcC.cadA;lysinedecarboxylase[EC:4.1.1.18]	-11.79107023	0.007257142
Metabolism;Aminoacidmetabolism;Lysinedegradation[PATH:ko00310];fadJ;3-hydroxyacyl-CoAdehydrogenase/enoyl-CoAhydratase/3-hydroxybutyryl-CoAepimerase[EC:1.1.1.354.2.1.175.1.2.3]	-12.25582329	0.010739859
Metabolism;Aminoacidmetabolism;Lysinedegradation[PATH:ko00310];gabD;succinate-semialdehydedehydrogenase/glutarate-semialdehydedehydrogenase[EC:1.2.1.161.2.1.791.2.1.20]	-4.549986885	1.37E-05
Metabolism;Aminoacidmetabolism;Phenylalanine.tyrosineandtryptophanbiosynthesis[PATH:ko00400];aroA;3-phosphoshikimate1-carboxyvinyltransferase[EC:2.5.1.19]	1.24780215	0.005802781
Metabolism;Aminoacidmetabolism;Phenylalanine.tyrosineandtryptophanbiosynthesis[PATH:ko00400];AROA2.aroA;3-deoxy-7-phosphoheptulonatesynthase[EC:2.5.1.54]	-5.965682707	3.03E-05
Metabolism;Aminoacidmetabolism;Phenylalanine.tyrosineandtryptophanbiosynthesis[PATH:ko00400];aroB;3-dehydroquinatesynthase[EC:4.2.3.4]	0.925983145	0.048249327
Metabolism;Aminoacidmetabolism;Phenylalanine.tyrosineandtryptophanbiosynthesis[PATH:ko00400];E2.7.1.71.aroK.aroL;shikimatekinase[EC:2.7.1.71]	1.050467488	0.021690776
Metabolism;Aminoacidmetabolism;Phenylalanine.tyrosineandtryptophanbiosynthesis[PATH:ko00400];hisC;histidinol-phosphateaminotransferase[EC:2.6.1.9]	-4.325886491	1.99E-06
Metabolism;Aminoacidmetabolism;Phenylalanine.tyrosineandtryptophanbiosynthesis[PATH:ko00400];trpC;indole-3-glycerolphosphatesynthase[EC:4.1.1.48]	-4.542391636	0.000331713
Metabolism;Aminoacidmetabolism;Phenylalanine.tyrosineandtryptophanbiosynthesis[PATH:ko00400];trpF;phosphoribosylanthranilateisomerase[EC:5.3.1.24]	0.705447491	0.034342378
Metabolism;Aminoacidmetabolism;Phenylalaninemetabolism[PATH:ko00360];E3.5.1.4.amiE;amidase[EC:3.5.1.4]	-9.159795129	0.012495682
Metabolism;Aminoacidmetabolism;Phenylalaninemetabolism[PATH:ko00360];hcaD;3-phenylpropionate/trans-cinnamatedioxygenaseferredoxinreductasesubunit[EC:1.18.1.3]	-11.80808924	0.015307575
Metabolism;Aminoacidmetabolism;Phenylalaninemetabolism[PATH:ko00360];hisC;histidinol-phosphateaminotransferase[EC:2.6.1.9]	-4.325886491	1.99E-06
Metabolism;Aminoacidmetabolism;Phenylalaninemetabolism[PATH:ko00360];katG;catalase-peroxidase[EC:1.11.1.21]	-11.79536398	0.007765704
Metabolism;Aminoacidmetabolism;Phenylalaninemetabolism[PATH:ko00360];paaJ;acetyl-CoAacetyltransferase[EC:2.3.1.-]	-11.80808924	0.015307575

Metabolism;Aminoacidmetabolism;Tryptophanmetabolism[PATH:ko00380];E2.3.1.9.atoB;acetyl-CoAC-acetyltransferase[EC:2.3.1.9]	-3.373422428	0.00346328
Metabolism;Aminoacidmetabolism;Tryptophanmetabolism[PATH:ko00380];E3.5.1.4.amiE;amidase[EC:3.5.1.4]	-9.159795129	0.012495682
Metabolism;Aminoacidmetabolism;Tryptophanmetabolism[PATH:ko00380];fadJ;3-hydroxyacyl-CoAdehydrogenase/enoyl-CoAhydratase/3-hydroxybutyryl-CoAepimerase[EC:1.1.1.354.2.1.175.1.2.3]	-12.25582329	0.010739859
Metabolism;Aminoacidmetabolism;Tryptophanmetabolism[PATH:ko00380];katG;catalase-peroxidase[EC:1.11.1.21]	-11.79536398	0.007765704
Metabolism;Aminoacidmetabolism;Tyrosinemetabolism[PATH:ko00350];adhE;acetaldehydedehydrogenase/alcoholdehydrogenase[EC:1.2.1.10 1.1.1.1]	-2.237328876	0.038521461
Metabolism;Aminoacidmetabolism;Tyrosinemetabolism[PATH:ko00350];adhP;alcoholdehydrogenase.propanol-preferring[EC:1.1.1.1]	-3.523029673	2.61E-05
Metabolism;Aminoacidmetabolism;Tyrosinemetabolism[PATH:ko00350];gabD;succinate-semialdehydedehydrogenase/glutarate-semialdehydedehydrogenase[EC:1.2.1.161.2.1.791.2.1.20]	-4.549986885	1.37E-05
Metabolism;Aminoacidmetabolism;Tyrosinemetabolism[PATH:ko00350];hisC;histidinol-phosphateaminotransferase[EC:2.6.1.9]	-4.325886491	1.99E-06
Metabolism;Aminoacidmetabolism;Valine.leucineandisoleucinebiosynthesis[PATH:ko00290];E2.6.1.42.ilvE;branched-chainaminoacidaminotransferase[EC:2.6.1.42]	-2.752257097	0.000346456
Metabolism;Aminoacidmetabolism;Valine.leucineandisoleucinebiosynthesis[PATH:ko00290];leuB;3-isopropylmalatedehydrogenase[EC:1.1.1.85]	-1.446471659	0.024792564
Metabolism;Aminoacidmetabolism;Valine.leucineandisoleucinebiosynthesis[PATH:ko00290];leuD;3-isopropylmalate/(R)-2-methylmalatedehydratasesmallsubunit[EC:4.2.1.334.2.1.35]	-2.749564252	0.000118783
Metabolism;Aminoacidmetabolism;Valine.leucineandisoleucinedegradation[PATH:ko00280];DLD.lpd.pdhD;dihydrolipoamidedehydrogenase[EC:1.8.1.4]	-6.375717808	2.61E-05
Metabolism;Aminoacidmetabolism;Valine.leucineandisoleucinedegradation[PATH:ko00280];E2.3.1.9.atoB;acetyl-CoAC-acetyltransferase[EC:2.3.1.9]	-3.373422428	0.00346328
Metabolism;Aminoacidmetabolism;Valine.leucineandisoleucinedegradation[PATH:ko00280];E2.6.1.42.ilvE;branched-chainaminoacidaminotransferase[EC:2.6.1.42]	-8.117069891	1.09E-07
Metabolism;Aminoacidmetabolism;Valine.leucineandisoleucinedegradation[PATH:ko00280];E6.4.1.4B;3-methylcrotonyl-CoAcarboxylasebetasubunit[EC:6.4.1.4]	-11.79536398	0.007765704
Metabolism;Aminoacidmetabolism;Valine.leucineandisoleucinedegradation[PATH:ko00280];fadJ;3-hydroxyacyl-CoAdehydrogenase/enoyl-CoAhydratase/3-hydroxybutyryl-CoAepimerase[EC:1.1.1.354.2.1.175.1.2.3]	-12.25582329	0.010739859
Metabolism;Biosynthesisofothersecondarymetabolites;Carbapenembiosynthesis[PATH:ko00332];proA;glutamate-5-semialdehydedehydrogenase[EC:1.2.1.41]	-6.929452986	8.78E-05
Metabolism;Biosynthesisofothersecondarymetabolites;Flavoneandflavonolbiosynthesis[PATH:ko00944];uidA.GUSB;beta-glucuronidase[EC:3.2.1.31]	-11.78042809	0.003902356
Metabolism;Biosynthesisofothersecondarymetabolites;Novobiocinbiosynthesis[PATH:ko00401];hisC;histidinol-phosphateaminotransferase[EC:2.6.1.9]	-4.325886491	1.99E-06
Metabolism;Biosynthesisofothersecondarymetabolites;Phenylpropanoidbiosynthesis[PATH:ko00940];bglX;beta-glucosidase[EC:3.2.1.21]	-11.77203009	0.003118189
Metabolism;Biosynthesisofothersecondarymetabolites;Phenylpropanoidbiosynthesis[PATH:ko00940];katG;catalase-peroxidase[EC:1.11.1.21]	-11.79536398	0.007765704
Metabolism;Biosynthesisofothersecondarymetabolites;Streptomycinbiosynthesis[PATH:ko00521];E4.2.1.46.rfbB.rffG;dTDP-glucose4.6-dehydratase[EC:4.2.1.46]	1.072919012	0.035526824
Metabolism;Biosynthesisofothersecondarymetabolites;Tropane.piperidineandpyridinealkaloidbiosynthesis[PATH:ko00960];E4.1.1.18.ldcC.cadA;lysinedecarboxylase[EC:4.1.1.18]	-11.79107023	0.007257142
Metabolism;Biosynthesisofothersecondarymetabolites;Tropane.piperidineandpyridinealkaloidbiosynthesis[PATH:ko00960];hisC;histidinol-phosphateaminotransferase[EC:2.6.1.9]	-4.325886491	1.99E-06

Metabolism;Carbohydratemetabolism;Aminosugarandnucleotidesugarmetabolism[PATH:ko00520];arnA.pmrI;UDP-4-amino-4-deoxy-L-arabinoseformyltransferase/UDP-glucuronicaciddehydrogenase(UDP-4-keto-hexauronicaciddecarboxylating)[EC:2.1.2.131.1.1.305]	-9.206051617	0.024593612
Metabolism;Carbohydratemetabolism;Aminosugarandnucleotidesugarmetabolism[PATH:ko00520];arnC.pmrF;undecaprenyl-phosphate4-deoxy-4-formamido-L-arabinosetransferase[EC:2.4.2.53]	-12.59923004	0.00766499
Metabolism;Carbohydratemetabolism;Aminosugarandnucleotidesugarmetabolism[PATH:ko00520];glmU;bifunctionalUDP-N-acetylglucosaminepyrophosphorylase/Glucosamine-1-phosphateN-acetyltransferase[EC:2.7.7.232.3.1.157]	-1.289772237	0.042564457
Metabolism;Carbohydratemetabolism;Aminosugarandnucleotidesugarmetabolism[PATH:ko00520];PTS-Man-EIID.manZ;PTSsystem.mannose-specificIIDcomponent	1.689056976	0.029533322
Metabolism;Carbohydratemetabolism;Aminosugarandnucleotidesugarmetabolism[PATH:ko00520];UGDH.ugd;UDPglucose6-dehydrogenase[EC:1.1.1.22]	-10.06115434	0.000112561
Metabolism;Carbohydratemetabolism;Aminosugarandnucleotidesugarmetabolism[PATH:ko00520];UGP2.galU.galF;UTP--glucose-1-phosphateuridylyltransferase[EC:2.7.7.9]	0.713947129	0.032725985
Metabolism;Carbohydratemetabolism;Aminosugarandnucleotidesugarmetabolism[PATH:ko00520];wbpO;UDP-N-acetyl-D-galactosaminedehydrogenase[EC:1.1.1.-]	-12.24908257	0.007708466
Metabolism;Carbohydratemetabolism;Aminosugarandnucleotidesugarmetabolism[PATH:ko00520];wecB;UDP-N-acetylglucosamine2-epimerase(non-hydrolysing)[EC:5.1.3.14]	-5.899726023	0.006457012
Metabolism;Carbohydratemetabolism;Aminosugarandnucleotidesugarmetabolism[PATH:ko00520];xynB;xylan1.4-beta-xylosidase[EC:3.2.1.37]	-6.215045099	1.45E-06
Metabolism;Carbohydratemetabolism;Citratecycle(TCAcycle)[PATH:ko00020];aceE;pyruvatedehydrogenaseE1component[EC:1.2.4.1]	-11.79245895	0.00766499
Metabolism;Carbohydratemetabolism;Citratecycle(TCAcycle)[PATH:ko00020];DLD.lpd.pdhD;dihydrolipoamidedehydrogenase[EC:1.8.1.4]	-6.375720181	2.61E-05
Metabolism;Carbohydratemetabolism;Citratecycle(TCAcycle)[PATH:ko00020];E4.2.1.2B.fumC;fumaratehydratase.classII[EC:4.2.1.2]	-3.270061347	0.027939722
Metabolism;Carbohydratemetabolism;Citratecycle(TCAcycle)[PATH:ko00020];frdA;fumaratereductaseflavoproteinsubunit[EC:1.3.5.4]	-3.39479139	0.012913123
Metabolism;Carbohydratemetabolism;Citratecycle(TCAcycle)[PATH:ko00020];IDH1.IDH2.icd;isocitratedehydrogenase[EC:1.1.1.42]	-2.763414464	0.038521461
Metabolism;Carbohydratemetabolism;Citratecycle(TCAcycle)[PATH:ko00020];sdhB;succinatedehydrogenaseiron-sulfursubunit[EC:1.3.5.1]	-9.543035019	0.016387029
Metabolism;Carbohydratemetabolism;Fructoseandmannosemetabolism[PATH:ko00051];FBA.fbaA;fructose-bisphosphatealdolase.classII[EC:4.1.2.13]	-6.482770493	1.53E-05
Metabolism;Carbohydratemetabolism;Fructoseandmannosemetabolism[PATH:ko00051];FBP.fbp;fructose-1.6-bisphosphataseI[EC:3.1.3.11]	-11.79608401	0.008749896
Metabolism;Carbohydratemetabolism;Fructoseandmannosemetabolism[PATH:ko00051];fruK;1-phosphofructokinase[EC:2.7.1.56]	1.272668773	0.001504431
Metabolism;Carbohydratemetabolism;Fructoseandmannosemetabolism[PATH:ko00051];fucA;L-fuculose-phosphatealdolase[EC:4.1.2.17]	-12.24908257	0.007708466
Metabolism;Carbohydratemetabolism;Fructoseandmannosemetabolism[PATH:ko00051];fucI;L-fucoseisomerase[EC:5.3.1.25]	-11.79719596	0.007765704
Metabolism;Carbohydratemetabolism;Fructoseandmannosemetabolism[PATH:ko00051];PTS-Fru-EIIA.fruB;PTSsystem.fructose-specificIIAcomponent[EC:2.7.1.69]	-5.590205876	0.000401184
Metabolism;Carbohydratemetabolism;Fructoseandmannosemetabolism[PATH:ko00051];PTS-Fru-EIIB.fruA;PTSsystem.fructose-specificIIBcomponent[EC:2.7.1.69]	-5.59151029	0.000401184
Metabolism;Carbohydratemetabolism;Fructoseandmannosemetabolism[PATH:ko00051];PTS-Fru-EIIC.fruA;PTSsystem.fructose-specificIICcomponent	-5.591510284	0.000401184
Metabolism;Carbohydratemetabolism;Fructoseandmannosemetabolism[PATH:ko00051];PTS-Man-EIID.manZ;PTSsystem.mannose-specificIIDcomponent	1.689056976	0.029533322
Metabolism;Carbohydratemetabolism;Fructoseandmannosemetabolism[PATH:ko00051];PTS-Mtl-EIIB.mtlA;PTSsystem.mannitol-specificIIBcomponent[EC:2.7.1.69]	-11.12095281	0.001653568

Metabolism;Carbohydratemetabolism;Fructoseandmannosemetabolism[PATH:ko00051];PTS-Mtl-EIIC.mtlA;PTSsystem.mannitol-specificIICcomponent	-11.12095281	0.001653568
Metabolism;Carbohydratemetabolism;Fructoseandmannosemetabolism[PATH:ko00051];rhaD;rhamnulose-1-phosphatealdolase[EC:4.1.2.19]	-9.817164929	0.011904792
Metabolism;Carbohydratemetabolism;Fructoseandmannosemetabolism[PATH:ko00051];rhmA;2-dehydro-3-deoxy-L-rhamnonatealdolase[EC:4.1.2.53]	-11.80991106	0.016700344
Metabolism;Carbohydratemetabolism;Fructoseandmannosemetabolism[PATH:ko00051];xylA;xyloseisomerase[EC:5.3.1.5]	-6.406312281	9.13E-08
Metabolism;Carbohydratemetabolism;Galactosemetabolism[PATH:ko00052];bgaB.lacA;beta-galactosidase[EC:3.2.1.23]	-10.58581923	2.42E-07
Metabolism;Carbohydratemetabolism;Galactosemetabolism[PATH:ko00052];E3.2.1.10;oligo-1.6-glucosidase[EC:3.2.1.10]	-5.480195436	2.42E-07
Metabolism;Carbohydratemetabolism;Galactosemetabolism[PATH:ko00052];E3.2.1.22B.galA.rafA;alpha-galactosidase[EC:3.2.1.22]	-5.507313066	2.05E-05
Metabolism;Carbohydratemetabolism;Galactosemetabolism[PATH:ko00052];ebgA;evolvedbeta-galactosidasesubunitalpha[EC:3.2.1.23]	-7.749288161	0.015307292
Metabolism;Carbohydratemetabolism;Galactosemetabolism[PATH:ko00052];glf;UDP-galactopyranosemutase[EC:5.4.99.9]	-1.683382157	0.00882928
Metabolism;Carbohydratemetabolism;Galactosemetabolism[PATH:ko00052];lacC;tagatose6-phosphatekinase[EC:2.7.1.144]	-3.600257432	0.041405281
Metabolism;Carbohydratemetabolism;Galactosemetabolism[PATH:ko00052];lacZ;beta-galactosidase[EC:3.2.1.23]	-5.671584301	3.48E-05
Metabolism;Carbohydratemetabolism;Galactosemetabolism[PATH:ko00052];malZ;alpha-glucosidase[EC:3.2.1.20]	-9.006626157	2.85E-07
Metabolism;Carbohydratemetabolism;Galactosemetabolism[PATH:ko00052];melA;alpha-galactosidase[EC:3.2.1.22]	-12.25454019	0.010249226
Metabolism;Carbohydratemetabolism;Galactosemetabolism[PATH:ko00052];PTS-Aga-EIIA.agaF;PTSsystem.N-acetylgalactosamine-specificIIAcomponent[EC:2.7.1.69]	-6.128638867	1.43E-06
Metabolism;Carbohydratemetabolism;Galactosemetabolism[PATH:ko00052];PTS-Gat-EIIA.gatA;PTSsystem.galactitol-specificIIAcomponent[EC:2.7.1.69]	-3.296106652	0.048719099
Metabolism;Carbohydratemetabolism;Galactosemetabolism[PATH:ko00052];PTS-Gat-EIIC.gatC;PTSsystem.galactitol-specificIICcomponent	-3.771901762	0.005986024
Metabolism;Carbohydratemetabolism;Galactosemetabolism[PATH:ko00052];PTS-Ula-EIIA.ulaC.sgaA;PTSsystem.ascorbate-specificIIAcomponent[EC:2.7.1.69]	-9.175545735	2.62E-07
Metabolism;Carbohydratemetabolism;Galactosemetabolism[PATH:ko00052];PTS-Ula-EIIB.ulaB.sgaB;PTSsystem.ascorbate-specificIIBcomponent[EC:2.7.1.69]	-4.517957613	0.000103131
Metabolism;Carbohydratemetabolism;Galactosemetabolism[PATH:ko00052];PTS-Ula-EIIC.ulaA.sgaT;PTSsystem.ascorbate-specificIICcomponent	-5.798272313	1.36E-06
Metabolism;Carbohydratemetabolism;Galactosemetabolism[PATH:ko00052];UGDH.ugd;UDPglucose6-dehydrogenase[EC:1.1.1.22]	-10.06115434	0.000112561
Metabolism;Carbohydratemetabolism;Galactosemetabolism[PATH:ko00052];UGP2.galU.galF;UTP--glucose-1-phosphateuridylyltransferase[EC:2.7.7.9]	0.713947129	0.032725985
Metabolism;Carbohydratemetabolism;Galactosemetabolism[PATH:ko00052];ulaG;L-ascorbate6-phosphatelactonase[EC:3.1.1.-]	-6.489220836	4.17E-07
Metabolism;Carbohydratemetabolism;Glycolysis/Gluconeogenesis[PATH:ko00010];aceE;pyruvatedehydrogenaseE1component[EC:1.2.4.1]	-11.79245895	0.00766499
Metabolism;Carbohydratemetabolism;Glycolysis/Gluconeogenesis[PATH:ko00010];adhE;acetaldehydedehydrogenase/alcoholdehydrogenase[EC:1.2.1.101.1.1.1]	-2.237328876	0.038521461
Metabolism;Carbohydratemetabolism;Glycolysis/Gluconeogenesis[PATH:ko00010];adhP;alcoholdehydrogenase.propanol-preferring[EC:1.1.1.1]	-3.523029673	2.61E-05
Metabolism;Carbohydratemetabolism;Glycolysis/Gluconeogenesis[PATH:ko00010];DLD.lpd.pdhD;dihydrolipoamidedehydrogenase[EC:1.8.1.4]	-6.375720181	2.61E-05
Metabolism;Carbohydratemetabolism;Glycolysis/Gluconeogenesis[PATH:ko00010];E3.2.1.86B.bglA;6-phospho-beta-glucosidase[EC:3.2.1.86]	-1.560020113	0.012785221
Metabolism;Carbohydratemetabolism;Glycolysis/Gluconeogenesis[PATH:ko00010];ENO.eno;enolase[EC:4.2.1.11]	-5.951624563	6.12E-05

Metabolism;Carbohydratemetabolism;Glycolysis/Gluconeogenesis[PATH:ko00010];FBA.fbaA;fructose-bisphosphatealdolase.classII[EC:4.1.2.13]	-6.482770493	1.53E-05
Metabolism;Carbohydratemetabolism;Glycolysis/Gluconeogenesis[PATH:ko00010];FBP.fbp;fructose-1.6-bisphosphataseI[EC:3.1.3.11]	-11.79608401	0.008749896
Metabolism;Carbohydratemetabolism;Glycolysis/Gluconeogenesis[PATH:ko00010];GAPDH.gapA;glyceraldehyde3-phosphatedehydrogenase[EC:1.2.1.12]	-5.46010237	0.000134338
Metabolism;Carbohydratemetabolism;Glycolysis/Gluconeogenesis[PATH:ko00010];LDH.ldh;L-lactatedehydrogenase[EC:1.1.1.27]	1.073131866	0.038702186
Metabolism;Carbohydratemetabolism;Glycolysis/Gluconeogenesis[PATH:ko00010];PTS-Arb-EIIB.glvB;PTSsystem.arbutin-likeIIBcomponent[EC:2.7.1.69]	-6.651053486	0.011057468
Metabolism;Carbohydratemetabolism;Glycolysis/Gluconeogenesis[PATH:ko00010];PTS-Arb-EIIC.glvC;PTSsystem.arbutin-likeIICcomponent	-6.651053486	0.011057468
Metabolism;Carbohydratemetabolism;Glyoxylateanddicarboxylatemetabolism[PATH:ko00630];accB.bccP;acetyl-CoAcarboxylasebiotincarboxylcarrierprotein	0.846959361	0.049618655
Metabolism;Carbohydratemetabolism;Glyoxylateanddicarboxylatemetabolism[PATH:ko00630];adhE;acetaldehydedehydrogenase/alcoholdehydrogenase[EC:1.2.1.101.1.1.1]	-2.237328876	0.038521461
Metabolism;Carbohydratemetabolism;Glyoxylateanddicarboxylatemetabolism[PATH:ko00630];aldA;lactaldehydedehydrogenase/glycolaldehydedehydrogenase[EC:1.2.1.221.2.1.21]	-9.591392669	0.000124308
Metabolism;Carbohydratemetabolism;Glyoxylateanddicarboxylatemetabolism[PATH:ko00630];butA.budC;meso-butanedioldehydrogenase/(S.S)-butanedioldehydrogenase/diacetylreductase[EC:1.1.1.-1.1.1.761.1.1.304]	-3.717248269	2.80E-05
Metabolism;Carbohydratemetabolism;Glyoxylateanddicarboxylatemetabolism[PATH:ko00630];E2.3.1.54.pflD;formateC-acetyltransferase[EC:2.3.1.54]	-5.45284466	0.002584061
Metabolism;Carbohydratemetabolism;Glyoxylateanddicarboxylatemetabolism[PATH:ko00630];E2.3.1.9.atoB;acetyl-CoAC-acetyltransferase[EC:2.3.1.9]	-3.373422428	0.00346328
Metabolism;Carbohydratemetabolism;Glyoxylateanddicarboxylatemetabolism[PATH:ko00630];E4.1.1.5.alsD;acetolactatedecarboxylase[EC:4.1.1.5]	0.973137017	0.001422087
Metabolism;Carbohydratemetabolism;Glyoxylateanddicarboxylatemetabolism[PATH:ko00630];E5.3.1.22.gip;hydroxypyruvateisomerase[EC:5.3.1.22]	-8.01390043	0.024792564
Metabolism;Carbohydratemetabolism;Glyoxylateanddicarboxylatemetabolism[PATH:ko00630];fadJ;3-hydroxyacyl-CoAdehydrogenase/enoyl-CoAhydratase/3-hydroxybutyryl-CoAepimerase[EC:1.1.1.354.2.1.175.1.2.3]	-12.25582329	0.010739859
Metabolism;Carbohydratemetabolism;Glyoxylateanddicarboxylatemetabolism[PATH:ko00630];fdoG.fdfH;formatedehydrogenasemajorsubunit[EC:1.2.1.2]	-6.762554455	0.004407149
Metabolism;Carbohydratemetabolism;Glyoxylateanddicarboxylatemetabolism[PATH:ko00630];frdA;fumaratereductaseflavoproteinsubunit[EC:1.3.5.4]	-3.39479139	0.012913123
Metabolism;Carbohydratemetabolism;Glyoxylateanddicarboxylatemetabolism[PATH:ko00630];gabD;succinate-semialdehydedehydrogenase/glutarate-semialdehydedehydrogenase[EC:1.2.1.161.2.1.791.2.1.20]	-4.549986885	1.37E-05
Metabolism;Carbohydratemetabolism;Glyoxylateanddicarboxylatemetabolism[PATH:ko00630];gcvH.GCSH;glycinecleavagesystemHprotein	-7.572877698	0.003467562
Metabolism;Carbohydratemetabolism;Glyoxylateanddicarboxylatemetabolism[PATH:ko00630];glcD;glycolateoxidase[EC:1.1.3.15]	-8.769486459	0.039283267
Metabolism;Carbohydratemetabolism;Glyoxylateanddicarboxylatemetabolism[PATH:ko00630];gldA;glyceroldehydrogenase[EC:1.1.1.6]	-5.911646545	2.42E-07
Metabolism;Carbohydratemetabolism;Glyoxylateanddicarboxylatemetabolism[PATH:ko00630];glyA.SHMT;glycinehydroxymethyltransferase[EC:2.1.2.1]	1.57364268	6.61E-07
Metabolism;Carbohydratemetabolism;Glyoxylateanddicarboxylatemetabolism[PATH:ko00630];LDH.ldh;L-lactatedehydrogenase[EC:1.1.1.27]	1.073131866	0.038702186

Metabolism;Carbohydratemetabolism;Glyoxylateanddicarboxylatemetabolism[PATH:ko00630];leuB;3-isopropylmalatedehydrogenase[EC:1.1.1.85]	-1.446471659	0.024792564
Metabolism;Carbohydratemetabolism;Glyoxylateanddicarboxylatemetabolism[PATH:ko00630];leuD;3-isopropylmalate/(R)-2-methylmalatedehydratasesmallsubunit[EC:4.2.1.334.2.1.35]	-2.749564252	0.000118783
Metabolism;Carbohydratemetabolism;Glyoxylateanddicarboxylatemetabolism[PATH:ko00630];puuE;4-aminobutyrateaminotransferase[EC:2.6.1.19]	-5.839008597	1.17E-05
Metabolism;Carbohydratemetabolism;Glyoxylateanddicarboxylatemetabolism[PATH:ko00630];sdhB;succinatedehydrogenaseiron-sulfursubunit[EC:1.3.5.1]	-9.543035019	0.016387029
Metabolism;Carbohydratemetabolism;Pentoseandglucuronateinterconversions[PATH:ko00040];araA;L-arabinoseisomerase[EC:5.3.1.4]	-5.847898782	4.20E-06
Metabolism;Carbohydratemetabolism;Pentoseandglucuronateinterconversions[PATH:ko00040];araD;L-ribulose-5-phosphate4-epimerase[EC:5.1.3.4]	-5.477300859	1.23E-07
Metabolism;Carbohydratemetabolism;Pentoseandglucuronateinterconversions[PATH:ko00040];kduD;2-deoxy-D-gluconate3-dehydrogenase[EC:1.1.1.125]	-5.405432446	1.51E-05
Metabolism;Carbohydratemetabolism;Pentoseandglucuronateinterconversions[PATH:ko00040];rhaD;rhamnulose-1-phosphatealdolase[EC:4.1.2.19]	-9.817164929	0.011904792
Metabolism;Carbohydratemetabolism;Pentoseandglucuronateinterconversions[PATH:ko00040];sgbH;3-dehydro-L-gulonate-6-phosphatedecarboxylase[EC:4.1.1.85]	-5.457570519	4.37E-05
Metabolism;Carbohydratemetabolism;Pentoseandglucuronateinterconversions[PATH:ko00040];sgbU;hexulose-6-phosphateisomerase[EC:5.-.-.-]	-4.509274122	4.02E-05
Metabolism;Carbohydratemetabolism;Pentoseandglucuronateinterconversions[PATH:ko00040];UGDH.ugd;UDPglucose6-dehydrogenase[EC:1.1.1.22]	-10.06115434	0.000112561
Metabolism;Carbohydratemetabolism;Pentoseandglucuronateinterconversions[PATH:ko00040];UGP2.galU.galF;UTP--glucose-1-phosphateuridylyltransferase[EC:2.7.7.9]	0.713947129	0.032725985
Metabolism;Carbohydratemetabolism;Pentoseandglucuronateinterconversions[PATH:ko00040];uidA.GUSB;beta-glucuronidase[EC:3.2.1.31]	-11.78042809	0.003902356
Metabolism;Carbohydratemetabolism;Pentoseandglucuronateinterconversions[PATH:ko00040];xylA;xyloseisomerase[EC:5.3.1.5]	-6.406312281	9.13E-08
Metabolism;Carbohydratemetabolism;Pentoseandglucuronateinterconversions[PATH:ko00040];xylB.XYLB;xylulokinase[EC:2.7.1.17]	-7.128084003	1.81E-09
Metabolism;Carbohydratemetabolism;Pentosephosphatepathway[PATH:ko00030];E2.2.1.2.talA.talB;transaldolase[EC:2.2.1.2]	-11.76644974	0.002995451
Metabolism;Carbohydratemetabolism;Pentosephosphatepathway[PATH:ko00030];E2.7.1.12.gntK.idnK;gluconokinase[EC:2.7.1.12]	-5.702007645	4.30E-05
Metabolism;Carbohydratemetabolism;Pentosephosphatepathway[PATH:ko00030];E4.1.2.9;phosphoketolase[EC:4.1.2.9]	-4.035204468	0.000344147
Metabolism;Carbohydratemetabolism;Pentosephosphatepathway[PATH:ko00030];FBA.fbaA;fructose-bisphosphatealdolase.classII[EC:4.1.2.13]	-6.482770493	1.53E-05
Metabolism;Carbohydratemetabolism;Pentosephosphatepathway[PATH:ko00030];FBP.fbp;fructose-1.6-bisphosphataseI[EC:3.1.3.11]	-11.79608401	0.008749896
Metabolism;Carbohydratemetabolism;Pentosephosphatepathway[PATH:ko00030];G6PD.zwf;glucose-6-phosphate1-dehydrogenase[EC:1.1.1.49]	-4.965135325	4.77E-07
Metabolism;Carbohydratemetabolism;Pentosephosphatepathway[PATH:ko00030];kdgK;2-dehydro-3-deoxygluconokinase[EC:2.7.1.45]	-6.233354308	7.70E-08
Metabolism;Carbohydratemetabolism;Pentosephosphatepathway[PATH:ko00030];PGD.gnd;6-phosphogluconatedehydrogenase[EC:1.1.1.44]	-4.864027831	2.76E-07
Metabolism;Carbohydratemetabolism;Pentosephosphatepathway[PATH:ko00030];pgl;6-phosphogluconolactonase[EC:3.1.1.31]	-6.034832737	6.61E-07
Metabolism;Carbohydratemetabolism;Pentosephosphatepathway[PATH:ko00030];rbsK.RBKS;ribokinase[EC:2.7.1.15]	-5.105090944	7.53E-07
Metabolism;Carbohydratemetabolism;Pyruvatemetabolism[PATH:ko00620];accB.bccP;acetyl-CoAcarboxylasebiotincarboxylcarrierprotein	0.846959361	0.049618655
Metabolism;Carbohydratemetabolism;Pyruvatemetabolism[PATH:ko00620];aceE;pyruvatedehydrogenaseE1component[EC:1.2.4.1]	-11.79245895	0.00766499
Metabolism;Carbohydratemetabolism;Pyruvatemetabolism[PATH:ko00620];acyP;acylphosphatase[EC:3.6.1.7]	-2.916448414	0.002995451

Metabolism;Carbohydratemetabolism;Pyruvatemetabolism[PATH:ko00620];adhE;acetaldehydedehydrogenase/alcoholdehydrogenase[EC:1.2.1.101.1.1.1]	-2.237328876	0.038521461
Metabolism;Carbohydratemetabolism;Pyruvatemetabolism[PATH:ko00620];aldA;lactaldehydedehydrogenase/glycolaldehydedehydrogenase[EC:1.2.1.221.2.1.21]	-9.591392669	0.000124308
Metabolism;Carbohydratemetabolism;Pyruvatemetabolism[PATH:ko00620];DLD.lpd.pdhD;dihydrolipoamidedehydrogenase[EC:1.8.1.4]	-6.375720181	2.61E-05
Metabolism;Carbohydratemetabolism;Pyruvatemetabolism[PATH:ko00620];dld;D-lactatedehydrogenase[EC:1.1.1.28]	-5.496561247	3.05E-05
Metabolism;Carbohydratemetabolism;Pyruvatemetabolism[PATH:ko00620];E1.2.3.3.poxL;pyruvateoxidase[EC:1.2.3.3]	-3.748915635	5.00E-05
Metabolism;Carbohydratemetabolism;Pyruvatemetabolism[PATH:ko00620];E2.3.1.54.pflD;formateC-acetyltransferase[EC:2.3.1.54]	-5.45284466	0.002584061
Metabolism;Carbohydratemetabolism;Pyruvatemetabolism[PATH:ko00620];E2.3.1.9.atoB;acetyl-CoAC-acetyltransferase[EC:2.3.1.9]	-3.373422428	0.00346328
Metabolism;Carbohydratemetabolism;Pyruvatemetabolism[PATH:ko00620];E4.2.1.2B.fumC;fumaratehydratase.classII[EC:4.2.1.2]	-3.270061347	0.027939722
Metabolism;Carbohydratemetabolism;Pyruvatemetabolism[PATH:ko00620];frdA;fumaratereductaseflavoproteinsubunit[EC:1.3.5.4]	-3.39479139	0.012913123
Metabolism;Carbohydratemetabolism;Pyruvatemetabolism[PATH:ko00620];LDH.ldh;L-lactatedehydrogenase[EC:1.1.1.27]	1.073131866	0.038702186
Metabolism;Carbohydratemetabolism;Pyruvatemetabolism[PATH:ko00620];ldhA;D-lactatedehydrogenase[EC:1.1.1.28]	-4.97732117	3.71E-07
Metabolism;Carbohydratemetabolism;Pyruvatemetabolism[PATH:ko00620];ppdK;pyruvate.orthophosphatedikinase[EC:2.7.9.1]	-9.358232779	0.000641867
Metabolism;Carbohydratemetabolism;Pyruvatemetabolism[PATH:ko00620];pps.ppsA;pyruvate.waterdikinase[EC:2.7.9.2]	-3.962193451	0.029219419
Metabolism;Carbohydratemetabolism;Starchandsucrosemetabolism[PATH:ko00500];bcsA;cellulosesynthase(UDP-forming)[EC:2.4.1.12]	-4.859827727	0.003902356
Metabolism;Carbohydratemetabolism;Starchandsucrosemetabolism[PATH:ko00500];bglX;beta-glucosidase[EC:3.2.1.21]	-11.77203009	0.003118189
Metabolism;Carbohydratemetabolism;Starchandsucrosemetabolism[PATH:ko00500];E2.4.1.1.glgP.PYG;starchphosphorylase[EC:2.4.1.1]	-6.048917334	0.001103521
Metabolism;Carbohydratemetabolism;Starchandsucrosemetabolism[PATH:ko00500];E2.4.1.7;sucrosephosphorylase[EC:2.4.1.7]	-6.528183172	1.48E-07
Metabolism;Carbohydratemetabolism;Starchandsucrosemetabolism[PATH:ko00500];E2.4.1.8.mapA;maltosephosphorylase[EC:2.4.1.8]	-5.503252856	6.90E-07
Metabolism;Carbohydratemetabolism;Starchandsucrosemetabolism[PATH:ko00500];E3.2.1.10;oligo-1.6-glucosidase[EC:3.2.1.10]	-5.480195436	2.42E-07
Metabolism;Carbohydratemetabolism;Starchandsucrosemetabolism[PATH:ko00500];E3.2.1.122.glvA;maltose-6'-phosphateglucosidase[EC:3.2.1.122]	-7.062080919	0.002705541
Metabolism;Carbohydratemetabolism;Starchandsucrosemetabolism[PATH:ko00500];E3.2.1.28.treA.treF;alpha.alpha-trehalase[EC:3.2.1.28]	-13.30306937	0.001164129
Metabolism;Carbohydratemetabolism;Starchandsucrosemetabolism[PATH:ko00500];malQ;4-alpha-glucanotransferase[EC:2.4.1.25]	-6.694938881	0.003049744
Metabolism;Carbohydratemetabolism;Starchandsucrosemetabolism[PATH:ko00500];malZ;alpha-glucosidase[EC:3.2.1.20]	-9.006626157	2.85E-07
Metabolism;Carbohydratemetabolism;Starchandsucrosemetabolism[PATH:ko00500];otsA;trehalose6-phosphatesynthase[EC:2.4.1.15]	-12.2522972	0.009026929
Metabolism;Carbohydratemetabolism;Starchandsucrosemetabolism[PATH:ko00500];pgmB;beta-phosphoglucomutase[EC:5.4.2.6]	-7.691695791	0.003902356
Metabolism;Carbohydratemetabolism;Starchandsucrosemetabolism[PATH:ko00500];treC;trehalose-6-phosphatehydrolase[EC:3.2.1.93]	-3.017443165	0.038521461
Metabolism;Carbohydratemetabolism;Starchandsucrosemetabolism[PATH:ko00500];treX.glgX;glycogenoperonprotein[EC:3.2.1.-]	-12.24041236	0.003851629
Metabolism;Carbohydratemetabolism;Starchandsucrosemetabolism[PATH:ko00500];UGDH.ugd;UDPglucose6-dehydrogenase[EC:1.1.1.22]	-10.06115434	0.000112561
Metabolism;Carbohydratemetabolism;Starchandsucrosemetabolism[PATH:ko00500];UGP2.galU.galF;UTP--glucose-1-phosphateuridylyltransferase[EC:2.7.7.9]	0.713947129	0.032725985
Metabolism;Carbohydratemetabolism;Starchandsucrosemetabolism[PATH:ko00500];uidA.GUSB;beta-glucuronidase[EC:3.2.1.31]	-11.78042809	0.003902356
Metabolism;Carbohydratemetabolism;Starchandsucrosemetabolism[PATH:ko00500];xynB;xylan1.4-beta-xylosidase[EC:3.2.1.37]	-6.215045099	1.45E-06

Metabolism;Energymetabolism;Nitrogenmetabolism[PATH:ko00910];arcC;carbamatekinase[EC:2.7.2.2]	-11.15287326	0.003472935
Metabolism;Energymetabolism;Nitrogenmetabolism[PATH:ko00910];cynT.can;carbonicanhydrase[EC:4.2.1.1]	2.451903192	0.016049109
Metabolism;Energymetabolism;Nitrogenmetabolism[PATH:ko00910];gltB;glutamatesynthase(NADPH/NADH)largechain[EC:1.4.1.131.4.1.14]	-7.841446025	0.000132326
Metabolism;Energymetabolism;Nitrogenmetabolism[PATH:ko00910];gltD;glutamatesynthase(NADPH/NADH)smallchain[EC:1.4.1.131.4.1.14]	-12.19765424	0.00100271
Metabolism;Energymetabolism;Nitrogenmetabolism[PATH:ko00910];ncd2.npd;nitronatemonooxygenase[EC:1.13.12.16]	-10.33978025	0.042908627
Metabolism;Energymetabolism;Nitrogenmetabolism[PATH:ko00910];nirB;nitritereductase(NADH)largesubunit[EC:1.7.1.15]	-11.78042809	0.003902356
Metabolism;Energymetabolism;Nitrogenmetabolism[PATH:ko00910];NRT.narK.nrtP.nasA;MFStransporter.NNPfamily.nitrate/nitritetransporter	-12.87188337	0.003472935
Metabolism;Energymetabolism;Oxidativephosphorylation[PATH:ko00190];ATPF0A.atpB;F-typeH+-transportingATPasesubunita	-1.529677463	0.023044057
Metabolism;Energymetabolism;Oxidativephosphorylation[PATH:ko00190];ATPF0B.atpF;F-typeH+-transportingATPasesubunitb	1.185859681	0.043106084
Metabolism;Energymetabolism;Oxidativephosphorylation[PATH:ko00190];ATPF1B.atpD;F-typeH+-transportingATPasesubunitbeta[EC:3.6.3.14]	-7.079413701	1.13E-05
Metabolism;Energymetabolism;Oxidativephosphorylation[PATH:ko00190];ATPF1D.atpH;F-typeH+-transportingATPasesubunitdelta	1.004732623	0.022656255
Metabolism;Energymetabolism;Oxidativephosphorylation[PATH:ko00190];ATPF1G.atpG;F-typeH+-transportingATPasesubunitgamma	-5.858814818	0.000372819
Metabolism;Energymetabolism;Oxidativephosphorylation[PATH:ko00190];coxA;cytochromecoxidasesubunitI[EC:1.9.3.1]	-11.80991106	0.016700344
Metabolism;Energymetabolism;Oxidativephosphorylation[PATH:ko00190];cydA;cytochromedubiquinoloxidasesubunitI[EC:1.10.3.-]	-5.329192263	1.17E-05
Metabolism;Energymetabolism;Oxidativephosphorylation[PATH:ko00190];cydB;cytochromedubiquinoloxidasesubunitII[EC:1.10.3.-]	-6.06163902	1.23E-07
Metabolism;Energymetabolism;Oxidativephosphorylation[PATH:ko00190];cyoB;cytochromeoubiquinoloxidasesubunitI[EC:1.10.3.-]	-9.486563045	0.006880452
Metabolism;Energymetabolism;Oxidativephosphorylation[PATH:ko00190];frdA;fumaratereductaseflavoproteinsubunit[EC:1.3.5.4]	-3.39479139	0.012913123
Metabolism;Energymetabolism;Oxidativephosphorylation[PATH:ko00190];ndh;NADHdehydrogenase[EC:1.6.99.3]	-4.997215855	5.99E-05
Metabolism;Energymetabolism;Oxidativephosphorylation[PATH:ko00190];nuoA;NADH-quinoneoxidoreductasesubunitA[EC:1.6.5.3]	-8.619570321	0.026503529
Metabolism;Energymetabolism;Oxidativephosphorylation[PATH:ko00190];nuoG;NADH-quinoneoxidoreductasesubunitG[EC:1.6.5.3]	-9.154070597	0.011532103
Metabolism;Energymetabolism;Oxidativephosphorylation[PATH:ko00190];nuoI;NADH-quinoneoxidoreductasesubunitI[EC:1.6.5.3]	-8.619570321	0.026503529
Metabolism;Energymetabolism;Oxidativephosphorylation[PATH:ko00190];nuoM;NADH-quinoneoxidoreductasesubunitM[EC:1.6.5.3]	-11.79536398	0.007765704
Metabolism;Energymetabolism;Oxidativephosphorylation[PATH:ko00190];ppk;polyphosphatekinase[EC:2.7.4.1]	-5.057087872	2.54E-05
Metabolism;Energymetabolism;Oxidativephosphorylation[PATH:ko00190];sdhB;succinatedehydrogenaseiron-sulfursubunit[EC:1.3.5.1]	-9.543035019	0.016387029
Metabolism;Glycanbiosynthesisandmetabolism;Glycosaminoglycandegradation[PATH:ko00531];uidA.GUSB;beta-glucuronidase[EC:3.2.1.31]	-11.78042809	0.003902356
Metabolism;Glycanbiosynthesisandmetabolism;Glycosphingolipidbiosynthesis-globoseries[PATH:ko00603];E3.2.1.22B.galA.rafA;alpha-galactosidase[EC:3.2.1.22]	-5.507313066	2.05E-05
Metabolism;Glycanbiosynthesisandmetabolism;Glycosphingolipidbiosynthesis-globoseries[PATH:ko00603];melA;alpha-galactosidase[EC:3.2.1.22]	-12.25454019	0.010249226
Metabolism;Glycanbiosynthesisandmetabolism;Lipopolysaccharidebiosynthesis[PATH:ko00540];kdsA;2-dehydro-3-deoxyphosphooctonatealdolase(KDO8-Psynthase)[EC:2.5.1.55]	-5.708904552	3.55E-07
Metabolism;Glycanbiosynthesisandmetabolism;Lipopolysaccharidebiosynthesis[PATH:ko00540];lpxB;lipid-A-disaccharidesynthase[EC:2.4.1.182]	-12.87221147	0.003552338
Metabolism;Glycanbiosynthesisandmetabolism;Lipopolysaccharidebiosynthesis[PATH:ko00540];waaB.rfaB;UDP-D-galactose:(glucosyl)LPSalpha-1.6-D-galactosyltransferase[EC:2.4.1.-]	-12.84017433	0.000658913

Metabolism;Glycanbiosynthesisandmetabolism;Lipopolysaccharidebiosynthesis[PATH:ko00540];waaL.rfaL;O-antigenligase[EC:6.-.-.-]	-12.24990814	0.007909432
Metabolism;Glycanbiosynthesisandmetabolism;Lipopolysaccharidebiosynthesisproteins[BR:ko01005];mrdA;penicillin-bindingprotein2	-12.25582329	0.010739859
Metabolism;Glycanbiosynthesisandmetabolism;Lipopolysaccharidebiosynthesisproteins[BR:ko01005];murE;UDP-N-acetylmuramoyl-L-alanyl-D-glutamate--2.6-diaminopimelateligase[EC:6.3.2.13]	-1.319193837	0.035849171
Metabolism;Glycanbiosynthesisandmetabolism;Lipopolysaccharidebiosynthesisproteins[BR:ko01005];pbpA;penicillin-bindingprotein1[EC:3.4.-.-]	-13.09558937	0.001238169
Metabolism;Glycanbiosynthesisandmetabolism;Lipopolysaccharidebiosynthesisproteins[BR:ko01005];pbpB;penicillin-bindingprotein2B	-5.410198846	3.05E-05
Metabolism;Glycanbiosynthesisandmetabolism;Otherglycandegradation[PATH:ko00511];ebgA;evolvedbeta-galactosidasesubunitalpha[EC:3.2.1.23]	-7.749288161	0.015307292
Metabolism;Glycanbiosynthesisandmetabolism;Otherglycandegradation[PATH:ko00511];lacZ;beta-galactosidase[EC:3.2.1.23]	-5.671584301	3.48E-05
Metabolism;Glycanbiosynthesisandmetabolism;Otherglycandegradation[PATH:ko00511];NEU1;sialidase-1[EC:3.2.1.18]	-8.737726906	0.025817995
Metabolism;Lipidmetabolism;Arachidonicacidmetabolism[PATH:ko00590];E1.11.1.9;glutathioneperoxidase[EC:1.11.1.9]	-5.514768616	3.08E-05
Metabolism;Lipidmetabolism;Biosynthesisofunsaturatedfattyacids[PATH:ko01040];fadJ;3-hydroxyacyl-CoAdehydrogenase/enoyl-CoAhydratase/3-hydroxybutyryl-CoAepimerase[EC:1.1.1.354.2.1.175.1.2.3]	-12.25582329	0.010739859
Metabolism;Lipidmetabolism;Fattyacidbiosynthesis[PATH:ko00061];accB.bccP;acetyl-CoAcarboxylasebiotincarboxylcarrierprotein	0.846959361	0.049618655
Metabolism;Lipidmetabolism;Fattyacidbiosynthesis[PATH:ko00061];fabI;enoyl-[acyl-carrierprotein]reductaseI[EC:1.3.1.91.3.1.10]	-4.200496577	0.026264115
Metabolism;Lipidmetabolism;Fattyacidbiosynthesis[PATH:ko00061];fabG;3-oxoacyl-[acyl-carrierprotein]reductase[EC:1.1.1.100]	-12.249148521	0.007708111
Metabolism;Lipidmetabolism;Fattyacidbiosynthesis[PATH:ko00061];fabD;[acyl-carrier-protein]S-malonyltransferase[EC:2.3.1.39]	-5.507314792	2.05E-05
Metabolism;Lipidmetabolism;Fattyacidbiosynthesis[PATH:ko00061];fabB;3-oxoacyl-[acyl-carrier-protein]synthaseI[EC:2.3.1.41]	-5.67158135	3.48E-05
Metabolism;Lipidmetabolism;Fattyacidbiosynthesis[PATH:ko00061];fabH;3-oxoacyl-[acyl-carrier-protein]synthaseIII[EC:2.3.1.180]	-12.25451469	0.010249151
Metabolism;Lipidmetabolism;Fattyacidbiosynthesis[PATH:ko00061];fabA;3-hydroxyacyl-[acyl-carrierprotein]dehydratase/trans-2-decenoyl-	-12.24914542	0.007708597
Metabolism;Lipidmetabolism;Fattyacidbiosynthesis[PATH:ko00061];fabK;enoyl-[acyl-carrierprotein]reductaseII[EC:1.3.1.9]	-23.48454267	0.000247784
Metabolism;Lipidmetabolism;Fattyacidbiosynthesis[PATH:ko00061];MCH;medium-chainacyl-[acyl-carrier-protein]hydrolase[EC:3.1.2.21]	-8.737733964	0.025818789
Metabolism;Lipidmetabolism;Fattyaciddegradation[PATH:ko00071];adhE;acetaldehydedehydrogenase/alcoholdehydrogenase[EC:1.2.1.101.1.1.1]	-7.78137147	3.62E-06
Metabolism;Lipidmetabolism;Fattyaciddegradation[PATH:ko00071];adhP;alcoholdehydrogenase.propanol-preferring[EC:1.1.1.1]	-3.523029673	2.61E-05
Metabolism;Lipidmetabolism;Fattyaciddegradation[PATH:ko00071];E2.3.1.9.atoB;acetyl-CoAC-acetyltransferase[EC:2.3.1.9]	-3.373422428	0.00346328
Metabolism;Lipidmetabolism;Fattyaciddegradation[PATH:ko00071];fadJ;3-hydroxyacyl-CoAdehydrogenase/enoyl-CoAhydratase/3-hydroxybutyryl-CoAepimerase[EC:1.1.1.354.2.1.175.1.2.3]	-12.25582329	0.010739859
Metabolism;Lipidmetabolism;Fattyaciddegradation[PATH:ko00071];hcaD;3-phenylpropionate/trans-cinnamatedioxygenaseferredoxinreductasesubunit[EC:1.18.1.3]	-11.80808924	0.015307575
Metabolism;Lipidmetabolism;GlyceroLipidmetabolism;[PATH:ko00561];E3.2.1.22B.galA.rafA;alpha-galactosidase[EC:3.2.1.22]	-5.507313066	2.05E-05
Metabolism;Lipidmetabolism;GlyceroLipidmetabolism;[PATH:ko00561];gldA;glyceroldehydrogenase[EC:1.1.1.6]	-5.911646545	2.42E-07
Metabolism;Lipidmetabolism;GlyceroLipidmetabolism;[PATH:ko00561];glpK.GK;glycerolkinase[EC:2.7.1.30]	-4.25237447	0.02262111

Metabolism;Lipidmetabolism;GlyceroLipidmetabolism;[PATH:ko00561];melA;alpha-galactosidase[EC:3.2.1.22]	-12.25454019	0.010249226
Metabolism;Lipidmetabolism;GlyceroLipidmetabolism;[PATH:ko00561];plsC;1-acyl-sn-glycerol-3-phosphateacyltransferase[EC:2.3.1.51]	-4.18632634	1.53E-05
Metabolism;Lipidmetabolism;GlycerophosphoLipidmetabolism;[PATH:ko00564];E3.1.4.46.glpQ.ugpQ;glycerophosphoryldiesterphosphodiesterase[EC:3.1.4.46]	-5.491990218	2.31E-07
Metabolism;Lipidmetabolism;GlycerophosphoLipidmetabolism;[PATH:ko00564];glpA.glpD;glycerol-3-phosphatedehydrogenase[EC:1.1.5.3]	-11.17474535	0.015307575
Metabolism;Lipidmetabolism;GlycerophosphoLipidmetabolism;[PATH:ko00564];plsC;1-acyl-sn-glycerol-3-phosphateacyltransferase[EC:2.3.1.51]	-4.18632634	1.53E-05
Metabolism;Lipidmetabolism;GlycerophosphoLipidmetabolism;[PATH:ko00564];ybhO;putativecardiolipinsynthase[EC:2.7.8.-]	-11.80991106	0.016700344
Metabolism;Lipidmetabolism;Steroidhormonebiosynthesis[PATH:ko00140];E3.1.6.1.aslA;arylsulfatase[EC:3.1.6.1]	-12.24908257	0.007708466
Metabolism;Metabolismofcofactorsandvitamins;Biotinmetabolism[PATH:ko00780];bioA;adenosylmethionine-8-amino-7-oxononanoateaminotransferase[EC:2.6.1.62]	-8.238907281	0.019121562
Metabolism;Metabolismofcofactorsandvitamins;Biotinmetabolism[PATH:ko00780];bioF;8-amino-7-oxononanoatesynthase[EC:2.3.1.47]	-11.17670975	0.00346328
Metabolism;Metabolismofcofactorsandvitamins;Biotinmetabolism[PATH:ko00780];birA;BirAfamilyTranscription;alregulator.biotinoperonrepressor/biotin-[acetyl-CoA-carboxylase]ligase[EC:6.3.4.15]	-1.906216563	0.003762574
Metabolism;Metabolismofcofactorsandvitamins;Biotinmetabolism[PATH:ko00780];fabI;enoyl-[acyl-carrierprotein]reductaseI[EC:1.3.1.91.3.1.10]	-4.200496577	0.026264115
Metabolism;Metabolismofcofactorsandvitamins;Lipoicacidmetabolism[PATH:ko00785];lplA;lipoate-proteinligaseA[EC:2.7.7.63]	0.662250911	0.042708858
Metabolism;Metabolismofcofactorsandvitamins;Nicotinateandnicotinamidemetabolism[PATH:ko00760];pncB.NAPRT1;nicotinatephosphoribosyltransferase[EC:6.3.4.21]	-2.194474502	0.012065563
Metabolism;Metabolismofcofactorsandvitamins;Nicotinateandnicotinamidemetabolism[PATH:ko00760];pntA;NAD(P)transhydrogenasesubunitalpha[EC:1.6.1.2]	-11.771402	0.003262402
Metabolism;Metabolismofcofactorsandvitamins;Nicotinateandnicotinamidemetabolism[PATH:ko00760];pntB;NAD(P)transhydrogenasesubunitbeta[EC:1.6.1.2]	-7.584308884	0.001497268
Metabolism;Metabolismofcofactorsandvitamins;Nicotinateandnicotinamidemetabolism[PATH:ko00760];sthA.udhA;NAD(P)transhydrogenase[EC:1.6.1.1]	-8.72149831	0.020955328
Metabolism;Metabolismofcofactorsandvitamins;Nicotinateandnicotinamidemetabolism[PATH:ko00760];ushA;5'-nucleotidase/UDP-sugardiphosphatase[EC:3.1.3.53.6.1.45]	-11.14418291	0.003902356
Metabolism;Metabolismofcofactorsandvitamins;Onecarbonpoolbyfolate[PATH:ko00670];E6.3.3.2;5-formyltetrahydrofolatecyclo-ligase[EC:6.3.3.2]	4.351269696	2.73E-06
Metabolism;Metabolismofcofactorsandvitamins;Onecarbonpoolbyfolate[PATH:ko00670];folD;methylenetetrahydrofolatedehydrogenase(NADP+)/methenyltetrahydrofolatecyclohydrolase[EC:1.5.1.53.5.4.9]	1.161964243	1.48E-06
Metabolism;Metabolismofcofactorsandvitamins;Onecarbonpoolbyfolate[PATH:ko00670];gcvT.AMT;aminomethyltransferase[EC:2.1.2.10]	-12.21984444	0.002190529
Metabolism;Metabolismofcofactorsandvitamins;Onecarbonpoolbyfolate[PATH:ko00670];glyA.SHMT;glycinehydroxymethyltransferase[EC:2.1.2.1]	1.57364268	6.61E-07
Metabolism;Metabolismofcofactorsandvitamins;Onecarbonpoolbyfolate[PATH:ko00670];metF.MTHFR;methylenetetrahydrofolatereductase(NADPH)[EC:1.5.1.20]	-1.73540505	0.007781973
Metabolism;Metabolismofcofactorsandvitamins;Onecarbonpoolbyfolate[PATH:ko00670];MTFMT.fmt;methionyl-tRNAformyltransferase[EC:2.1.2.9]	0.89200961	0.002995451
Metabolism;Metabolismofcofactorsandvitamins;PantothenateandCoAbiosynthesis[PATH:ko00770];acpS;holo-[acyl-carrierprotein]synthase[EC:2.7.8.7]	1.797149853	3.02E-05
Metabolism;Metabolismofcofactorsandvitamins;PantothenateandCoAbiosynthesis[PATH:ko00770];coaA;typeIpantothenatekinase[EC:2.7.1.33]	-3.183807136	0.000132326

Metabolism;Metabolismofcofactorsandvitamins;PantothenateandCoAbiosynthesis[PATH:ko00770];coaBC.dfp;phosphopantothenoylcysteinedecarboxylase/phosphopantothenate--cysteineligase[EC:4.1.1.366.3.2.5]	-4.62304432	0.000161726
Metabolism;Metabolismofcofactorsandvitamins;PantothenateandCoAbiosynthesis[PATH:ko00770];E2.6.1.42.ilvE;branched-chainaminoacidaminotransferase[EC:2.6.1.42]	-2.752257097	0.000346456
Metabolism;Metabolismofcofactorsandvitamins;PantothenateandCoAbiosynthesis[PATH:ko00770];LYS5.acpT;4'-phosphopantetheinyltransferase[EC:2.7.8.-]	-8.454479247	0.000571588
Metabolism;Metabolismofcofactorsandvitamins;Retinolmetabolism[PATH:ko00830];adhP;alcoholdehydrogenase.propanol-preferring[EC:1.1.1.1]	-3.523029673	2.61E-05
Metabolism;Metabolismofcofactorsandvitamins;Retinolmetabolism[PATH:ko00830];cysG;uroporphyrin-IIIC-methyltransferase/precorrin-2dehydrogenase/sirohydrochlorinferrochelatase[EC:2.1.1.1071.3.1.764.99.1.4]	-12.25721895	0.011710324
Metabolism;Metabolismofcofactorsandvitamins;Retinolmetabolism[PATH:ko00830];EARS.gltX;glutamyl-tRNAsynthetase[EC:6.1.1.17]	-8.769486459	0.039283267
Metabolism;Metabolismofcofactorsandvitamins;Retinolmetabolism[PATH:ko00830];uidA.GUSB;beta-glucuronidase[EC:3.2.1.31]	-11.78042809	0.003902356
Metabolism;Metabolismofcofactorsandvitamins;Riboflavinmetabolism[PATH:ko00740];ribE.RIB5;riboflavinsynthase[EC:2.5.1.9]	-11.16547026	0.010541687
Metabolism;Metabolismofcofactorsandvitamins;Thiaminemetabolism[PATH:ko00730];dxs;1-deoxy-D-xylulose-5-phosphatesynthase[EC:2.2.1.7]	-6.229850367	0.019768035
Metabolism;Metabolismofcofactorsandvitamins;Thiaminemetabolism[PATH:ko00730];tenA;thiaminase(Transcription;alactivatorTenA)[EC:3.5.99.2]	-7.098204951	4.37E-08
Metabolism;Metabolismofcofactorsandvitamins;Thiaminemetabolism[PATH:ko00730];thiD;hydroxymethylpyrimidine/phosphomethylpyrimidinekinase[EC:2.7.1.492.7.4.7]	-5.462044542	3.40E-07
Metabolism;Metabolismofcofactorsandvitamins;Thiaminemetabolism[PATH:ko00730];thiE;thiamine-phosphatepyrophosphorylase[EC:2.5.1.3]	-4.842846205	0.000680763
Metabolism;Metabolismofcofactorsandvitamins;Thiaminemetabolism[PATH:ko00730];thiM;hydroxyethylthiazolekinase[EC:2.7.1.50]	-6.265058553	2.32E-07
Metabolism;Metabolismofcofactorsandvitamins;Ubiquinoneandotherterpenoid-quinonebiosynthesis[PATH:ko00130];menA;1.4-dihydroxy-2-naphthoateoctaprenyltransferase[EC:2.5.1.742.5.1.-]	-3.705492221	2.05E-05
Metabolism;Metabolismofcofactorsandvitamins;Ubiquinoneandotherterpenoid-quinonebiosynthesis[PATH:ko00130];menB;naphthoatesynthase[EC:4.1.3.36]	-5.662348449	2.85E-07
Metabolism;Metabolismofcofactorsandvitamins;Ubiquinoneandotherterpenoid-quinonebiosynthesis[PATH:ko00130];menC;O-succinylbenzoatesynthase[EC:4.2.1.113]	-6.150562841	5.96E-06
Metabolism;Metabolismofcofactorsandvitamins;Ubiquinoneandotherterpenoid-quinonebiosynthesis[PATH:ko00130];menD;2-succinyl-5-enolpyruvyl-6-hydroxy-3-cyclohexene-1-carboxylatesynthase[EC:2.2.1.9]	-5.67901844	2.76E-07
Metabolism;Metabolismofcofactorsandvitamins;Ubiquinoneandotherterpenoid-quinonebiosynthesis[PATH:ko00130];menE;O-succinylbenzoicacid--CoAligase[EC:6.2.1.26]	-5.130738797	2.75E-05
Metabolism;Metabolismofcofactorsandvitamins;Ubiquinoneandotherterpenoid-quinonebiosynthesis[PATH:ko00130];menF;menaquinone-specificisochorismatesynthase[EC:5.4.4.2]	-6.584201842	2.31E-07
Metabolism;Metabolismofcofactorsandvitamins;Ubiquinoneandotherterpenoid-quinonebiosynthesis[PATH:ko00130];menH;2-succinyl-6-hydroxy-2.4-cyclohexadiene-1-carboxylatesynthase[EC:4.2.99.20]	-8.457238479	2.32E-08
Metabolism;Metabolismofcofactorsandvitamins;Ubiquinoneandotherterpenoid-quinonebiosynthesis[PATH:ko00130];ubiE;ubiquinone/menaquinonebiosynthesismethyltransferase[EC:2.1.1.1632.1.1.201]	-5.869040184	5.45E-06
Metabolism;Metabolismofcofactorsandvitamins;VitaminB6metabolism[PATH:ko00750];pdxK.pdxY;pyridoxinekinase[EC:2.7.1.35]	-1.729055447	0.00766499
Metabolism;Metabolismofcofactorsandvitamins;VitaminB6metabolism[PATH:ko00750];serC.PSAT1;phosphoserineaminotransferase[EC:2.6.1.52]	-2.871256379	0.000521046

Metabolism;Metabolismofotheraminoacids;beta-Alaninemetabolism[PATH:ko00410];fadJ;3-hydroxyacyl-CoAdehydrogenase/enoyl-CoAhydratase/3-hydroxybutyryl-CoAepimerase[EC:1.1.1.354.2.1.175.1.2.3]	-12.25582329	0.010739859
Metabolism;Metabolismofotheraminoacids;beta-Alaninemetabolism[PATH:ko00410];prr;aminobutyraldehydedehydrogenase[EC:1.2.1.19]	-11.81146043	0.018385998
Metabolism;Metabolismofotheraminoacids;beta-Alaninemetabolism[PATH:ko00410];puuE;4-aminobutyrateaminotransferase[EC:2.6.1.19]	-5.839008597	1.17E-05
Metabolism;Metabolismofotheraminoacids;Cyanoaminoacidmetabolism[PATH:ko00460];bglX;beta-glucosidase[EC:3.2.1.21]	-11.77203009	0.003118189
Metabolism;Metabolismofotheraminoacids;Cyanoaminoacidmetabolism[PATH:ko00460];glyA.SHMT;glycinehydroxymethyltransferase[EC:2.1.2.1]	1.57364268	6.61E-07
Metabolism;Metabolismofotheraminoacids;Glutathionemetabolism[PATH:ko00480];CARP.pepA;leucylaminopeptidase[EC:3.4.11.1]	-5.161530567	0.000625531
Metabolism;Metabolismofotheraminoacids;Glutathionemetabolism[PATH:ko00480];E1.11.1.9;glutathioneperoxidase[EC:1.11.1.9]	-5.514768616	3.08E-05
Metabolism;Metabolismofotheraminoacids;Glutathionemetabolism[PATH:ko00480];E4.1.1.17.ODC1.speC.speF;ornithinedecarboxylase[EC:4.1.1.17]	-9.436250667	0.002995451
Metabolism;Metabolismofotheraminoacids;Glutathionemetabolism[PATH:ko00480];G6PD.zwf;glucose-6-phosphate1-dehydrogenase[EC:1.1.1.49]	-4.965135325	4.77E-07
Metabolism;Metabolismofotheraminoacids;Glutathionemetabolism[PATH:ko00480];IDH1.IDH2.icd;isocitratedehydrogenase[EC:1.1.1.42]	-2.763414464	0.038521461
Metabolism;Metabolismofotheraminoacids;Glutathionemetabolism[PATH:ko00480];pepB;PepBaminopeptidase[EC:3.4.11.23]	-5.706615237	0.000287658
Metabolism;Metabolismofotheraminoacids;Glutathionemetabolism[PATH:ko00480];pepD;dipeptidaseD[EC:3.4.13.-]	-7.596152366	0.000603532
Metabolism;Metabolismofotheraminoacids;Glutathionemetabolism[PATH:ko00480];pepN;aminopeptidaseN[EC:3.4.11.2]	-7.651072919	1.47E-06
Metabolism;Metabolismofotheraminoacids;Glutathionemetabolism[PATH:ko00480];PGD.gnd;6-phosphogluconatedehydrogenase[EC:1.1.1.44]	-4.864027831	2.76E-07
Metabolism;Metabolismofotheraminoacids;Selenocompoundmetabolism[PATH:ko00450];metC;cystathioninebeta-lyase[EC:4.4.1.8]	1.143510255	0.007708466
Metabolism;Metabolismofotheraminoacids;Taurineandhypotaurinemetabolism[PATH:ko00430];ald;alaninedehydrogenase[EC:1.4.1.1]	-5.521648089	0.009264107
Metabolism;Metabolismofterpenoidsandpolyketides;Biosynthesisofsiderophoregroupnonribosomalpeptides[PATH:ko01053];menF;menaquinone-specificisochorismatesynthase[EC:5.4.4.2]	-6.584201842	2.31E-07
Metabolism;Metabolismofterpenoidsandpolyketides;Biosynthesisofvancomycingroupantibiotics[PATH:ko01055];E4.2.1.46.rfbB.rffG;dTDP-glucose4.6-dehydratase[EC:4.2.1.46]	1.072919012	0.035526824
Metabolism;Metabolismofterpenoidsandpolyketides;Carotenoidbiosynthesis[PATH:ko00906];crtB;phytoenesynthase[EC:2.5.1.32]	-6.365725146	6.94E-05
Metabolism;Metabolismofterpenoidsandpolyketides;Carotenoidbiosynthesis[PATH:ko00906];crtN;4.4'-diapophytoenedesaturase[EC:1.3.8.2]	-13.30782884	3.48E-05
Metabolism;Metabolismofterpenoidsandpolyketides;Geranioldegradation[PATH:ko00281];fadJ;3-hydroxyacyl-CoAdehydrogenase/enoyl-CoAhydratase/3-hydroxybutyryl-CoAepimerase[EC:1.1.1.354.2.1.175.1.2.3]	-12.25582329	0.010739859
Metabolism;Metabolismofterpenoidsandpolyketides;Limoneneandpinenedegradation[PATH:ko00903];fadJ;3-hydroxyacyl-CoAdehydrogenase/enoyl-CoAhydratase/3-hydroxybutyryl-CoAepimerase[EC:1.1.1.354.2.1.175.1.2.3]	-12.25582329	0.010739859
Metabolism;Metabolismofterpenoidsandpolyketides;Nonribosomalpeptidestructures[PATH:ko01054];E5.1.1.13;aspartateracemase[EC:5.1.1.13]	-4.942524921	0.012913123
Metabolism;Metabolismofterpenoidsandpolyketides;Polyketidesugarunitbiosynthesis[PATH:ko00523];E4.2.1.46.rfbB.rffG;dTDP-glucose4.6-dehydratase[EC:4.2.1.46]	1.072919012	0.035526824
Metabolism;Metabolismofterpenoidsandpolyketides;Terpenoidbackbonebiosynthesis[PATH:ko00900];dxs;1-deoxy-D-xylulose-5-phosphatesynthase[EC:2.2.1.7]	-6.229850367	0.019768035

Metabolism;Metabolismofterpenoidsandpolyketides;Terpenoidbackbone biosynthesis[PATH:ko00900];E1.17.7.1.gcpE.ispG;(E)-4-hydroxy-3-methylbut-2-enyl-diphosphatesynthase[EC:1.17.7.1]	-11.16530237	0.001348297
Metabolism;Metabolismofterpenoidsandpolyketides;Terpenoidbackbone biosynthesis[PATH:ko00900];E2.3.1.9.atoB;acetyl-CoAC-acetyltransferase[EC:2.3.1.9]	-3.373422428	0.00346328
Metabolism;Metabolismofterpenoidsandpolyketides;Terpenoidbackbone biosynthesis[PATH:ko00900];hepST;heptaprenyldiphosphatesynthase[EC:2.5.1.30]	-5.468788894	7.53E-07
Metabolism;Metabolismofterpenoidsandpolyketides;Terpenoidbackbone biosynthesis[PATH:ko00900];ispD;2-C-methyl-D-erythritol4-phosphatecytidylyltransferase[EC:2.7.7.60]	-11.77098915	0.00346328
Metabolism;Metabolismofterpenoidsandpolyketides;Terpenoidbackbone biosynthesis[PATH:ko00900];MVD.mvaD;diphosphomevalonatedecarboxylase[EC:4.1.1.33]	0.613401277	0.039704551
Metabolism;Metabolismofterpenoidsandpolyketides;Tetracyclinebiosynthesis[PATH:ko00253];accB.bccP;acetyl-CoAcarboxylasebiotincarboxylcarrierprotein	0.846959361	0.049618655
Metabolism;Metabolismofterpenoidsandpolyketides;Zeatinbiosynthesis[PATH:ko00908];miaA.TRIT1;tRNAdimethylallyltransferase[EC:2.5.1.75]	-1.383946873	0.03049584
Metabolism;Nucleotidemetabolism;Purinemetabolism[PATH:ko00230];ade;adeninedeaminase[EC:3.5.4.2]	-5.402962856	2.50E-05
Metabolism;Nucleotidemetabolism;Purinemetabolism[PATH:ko00230];APRT.apt;adeninephosphoribosyltransferase[EC:2.4.2.7]	-1.931540789	0.010739859
Metabolism;Nucleotidemetabolism;Purinemetabolism[PATH:ko00230];arcC;carbamatekinase[EC:2.7.2.2]	-11.15287326	0.003472935
Metabolism;Nucleotidemetabolism;Purinemetabolism[PATH:ko00230];dgt;dGTPase[EC:3.1.5.1]	-12.184505	0.000642141
Metabolism;Nucleotidemetabolism;Purinemetabolism[PATH:ko00230];DPO3D1.holA;DNApolymeraseIIIsubunitdelta[EC:2.7.7.7]	-2.057134442	0.001769558
Metabolism;Nucleotidemetabolism;Purinemetabolism[PATH:ko00230];DPO3E.dnaQ;DNApolymeraseIIIsubunitepsilon[EC:2.7.7.7]	-2.281934658	0.014968485
Metabolism;Nucleotidemetabolism;Purinemetabolism[PATH:ko00230];E1.17.4.1A.nrdA.nrdE;ribonucleoside-diphosphatereductasealphachain[EC:1.17.4.1]	-1.864005504	0.016073075
Metabolism;Nucleotidemetabolism;Purinemetabolism[PATH:ko00230];E1.17.4.1B.nrdB.nrdF;ribonucleoside-diphosphatereductasebetachain[EC:1.17.4.1]	-1.671837133	0.016557857
Metabolism;Nucleotidemetabolism;Purinemetabolism[PATH:ko00230];E1.7.1.7.guaC;GMPreductase[EC:1.7.1.7]	-7.20639124	0.000767427
Metabolism;Nucleotidemetabolism;Purinemetabolism[PATH:ko00230];nudF;ADP-ribosepyrophosphatase[EC:3.6.1.13]	-1.93227125	0.009171493
Metabolism;Nucleotidemetabolism;Purinemetabolism[PATH:ko00230];ppx-gppA;exopolyphosphatase/guanosine-5'-triphosphate.3'-diphosphatepyrophosphatase[EC:3.6.1.113.6.1.40]	-5.507859918	7.70E-08
Metabolism;Nucleotidemetabolism;Purinemetabolism[PATH:ko00230];purB.ADSL;adenylosuccinatelyase[EC:4.3.2.2]	1.408979944	0.000660017
Metabolism;Nucleotidemetabolism;Purinemetabolism[PATH:ko00230];purD;phosphoribosylamine--glycineligase[EC:6.3.4.13]	0.959229612	4.69E-05
Metabolism;Nucleotidemetabolism;Purinemetabolism[PATH:ko00230];purE;5-(carboxyamino)imidazoleribonucleotidemutase[EC:5.4.99.18]	0.900720113	0.001873837
Metabolism;Nucleotidemetabolism;Purinemetabolism[PATH:ko00230];purK;5-(carboxyamino)imidazoleribonucleotidesynthase[EC:6.3.4.18]	0.747616177	0.00158021
Metabolism;Nucleotidemetabolism;Purinemetabolism[PATH:ko00230];purL.PFAS;phosphoribosylformylglycinamidinesynthase[EC:6.3.5.3]	-5.800440217	0.000280125
Metabolism;Nucleotidemetabolism;Purinemetabolism[PATH:ko00230];rpoZ;DNA-directedRNApolymerasesubunitomega[EC:2.7.7.6]	1.140001556	0.003577006
Metabolism;Nucleotidemetabolism;Purinemetabolism[PATH:ko00230];ushA;5'-nucleotidase/UDP-sugardiphosphatase[EC:3.1.3.53.6.1.45]	-11.14418291	0.003902356
Metabolism;Nucleotidemetabolism;Purinemetabolism[PATH:ko00230];ygeT.xdhB;xanthinedehydrogenaseFAD-bindingsubunit[EC:1.17.1.4]	-9.543035019	0.016387029
Metabolism;Nucleotidemetabolism;Pyrimidinemetabolism[PATH:ko00240];codA;cytosinedeaminase[EC:3.5.4.1]	-3.331975975	0.02302363
Metabolism;Nucleotidemetabolism;Pyrimidinemetabolism[PATH:ko00240];DPO3D1.holA;DNApolymeraseIIIsubunitdelta[EC:2.7.7.7]	-2.057134442	0.001769558

Metabolism;Nucleotidemetabolism;Pyrimidinemetabolism[PATH:ko00240];DPO3E.dnaQ;DNApolymeraseIIIsubunitepsilon[EC:2.7.7.7]	-2.281934658	0.014968485
Metabolism;Nucleotidemetabolism;Pyrimidinemetabolism[PATH:ko00240];E1.17.4.1A.nrdA.nrdE;ribonucleoside-diphosphatereductasealphachain[EC:1.17.4.1]	-1.864005504	0.016073075
Metabolism;Nucleotidemetabolism;Pyrimidinemetabolism[PATH:ko00240];E1.17.4.1B.nrdB.nrdF;ribonucleoside-diphosphatereductasebetachain[EC:1.17.4.1]	-1.671837133	0.016557857
Metabolism;Nucleotidemetabolism;Pyrimidinemetabolism[PATH:ko00240];E2.4.2.6;nucleosidedeoxyribosyltransferase[EC:2.4.2.6]	-5.649851152	2.54E-05
Metabolism;Nucleotidemetabolism;Pyrimidinemetabolism[PATH:ko00240];pyrB.PYR2;aspartatecarbamoyltransferasecatalyticsubunit[EC:2.1.3.2]	0.766457577	0.047424644
Metabolism;Nucleotidemetabolism;Pyrimidinemetabolism[PATH:ko00240];pyrE;orotatephosphoribosyltransferase[EC:2.4.2.10]	1.328705156	0.002813251
Metabolism;Nucleotidemetabolism;Pyrimidinemetabolism[PATH:ko00240];pyrR;pyrimidineoperonattenuationprotein/uracilphosphoribosyltransferase[EC:2.4.2.9]	-2.227924036	0.004861897
Metabolism;Nucleotidemetabolism;Pyrimidinemetabolism[PATH:ko00240];rpoZ;DNA-directedRNApolymerasesubunitomega[EC:2.7.7.6]	1.140001556	0.003577006
Metabolism;Nucleotidemetabolism;Pyrimidinemetabolism[PATH:ko00240];tdk.TK;thymidinekinase[EC:2.7.1.21]	-1.497942543	0.011295792
Metabolism;Nucleotidemetabolism;Pyrimidinemetabolism[PATH:ko00240];tmk.DTYMK;dTMPkinase[EC:2.7.4.9]	-1.232727497	0.03859021
Metabolism;Nucleotidemetabolism;Pyrimidinemetabolism[PATH:ko00240];udk.UCK;uridinekinase[EC:2.7.1.48]	-2.215277024	0.002602884
Metabolism;Nucleotidemetabolism;Pyrimidinemetabolism[PATH:ko00240];udp.UPP;uridinephosphorylase[EC:2.4.2.3]	-8.044482887	0.002872349
Metabolism;Nucleotidemetabolism;Pyrimidinemetabolism[PATH:ko00240];upp.UPRT;uracilphosphoribosyltransferase[EC:2.4.2.9]	1.001057449	0.014968485
Metabolism;Nucleotidemetabolism;Pyrimidinemetabolism[PATH:ko00240];ushA;5'-nucleotidase/UDP-sugardiphosphatase[EC:3.1.3.53.6.1.45]	-11.14418291	0.003902356
Metabolism;OverviewBiosynthesisofaminoacids[PATH:ko01230];argC;N-acetyl-gamma-glutamyl-phosphatereductase[EC:1.2.1.38]	-3.656685122	0.004196998
Metabolism;OverviewBiosynthesisofaminoacids[PATH:ko01230];argD;acetylornithine/N-succinyldiaminopimelateaminotransferase[EC:2.6.1.112.6.1.17]	-11.79756423	0.009303631
Metabolism;OverviewBiosynthesisofaminoacids[PATH:ko01230];argG.ASS1;argininosuccinatesynthase[EC:6.3.4.5]	-2.488905339	0.001164129
Metabolism;OverviewBiosynthesisofaminoacids[PATH:ko01230];argH.ASL;argininosuccinatelyase[EC:4.3.2.1]	-3.714692095	1.59E-05
Metabolism;OverviewBiosynthesisofaminoacids[PATH:ko01230];aroA;3-phosphoshikimate1-carboxyvinyltransferase[EC:2.5.1.19]	1.24780215	0.005802781
Metabolism;OverviewBiosynthesisofaminoacids[PATH:ko01230];AROA2.aroA;3-deoxy-7-phosphoheptulonatesynthase[EC:2.5.1.54]	-5.965682707	3.03E-05
Metabolism;OverviewBiosynthesisofaminoacids[PATH:ko01230];aroB;3-dehydroquinatesynthase[EC:4.2.3.4]	0.925983145	0.048249327
Metabolism;OverviewBiosynthesisofaminoacids[PATH:ko01230];dapA;4-hydroxy-tetrahydrodipicolinatesynthase[EC:4.3.3.7]	-1.322063751	0.038521461
Metabolism;OverviewBiosynthesisofaminoacids[PATH:ko01230];dapB;4-hydroxy-tetrahydrodipicolinatereductase[EC:1.17.1.8]	1.3854821	2.54E-05
Metabolism;OverviewBiosynthesisofaminoacids[PATH:ko01230];dapC;N-succinyldiaminopimelateaminotransferase[EC:2.6.1.17]	-11.141225	0.001653568
Metabolism;OverviewBiosynthesisofaminoacids[PATH:ko01230];E1.1.1.3;homoserinedehydrogenase[EC:1.1.1.3]	-1.457338016	0.02429635
Metabolism;OverviewBiosynthesisofaminoacids[PATH:ko01230];E1.5.1.2.proC;pyrroline-5-carboxylatereductase[EC:1.5.1.2]	-1.183816916	0.024547842
Metabolism;OverviewBiosynthesisofaminoacids[PATH:ko01230];E2.2.1.2.talA.talB;transaldolase[EC:2.2.1.2]	-11.76644974	0.002995451
Metabolism;OverviewBiosynthesisofaminoacids[PATH:ko01230];E2.6.1.42.ilvE;branched-chainaminoacidaminotransferase[EC:2.6.1.42]	-2.752257097	0.000346456
Metabolism;OverviewBiosynthesisofaminoacids[PATH:ko01230];E2.7.1.71.aroK.aroL;shikimatekinase[EC:2.7.1.71]	1.050467488	0.021690776
Metabolism;OverviewBiosynthesisofaminoacids[PATH:ko01230];E3.1.3.15B;histidinol-phosphatase(PHPfamily)[EC:3.1.3.15]	-7.431575909	1.14E-08
Metabolism;OverviewBiosynthesisofaminoacids[PATH:ko01230];ENO.eno;enolase[EC:4.2.1.11]	-5.951624563	6.12E-05

Metabolism;OverviewBiosynthesisofaminoacids[PATH:ko01230];FBA.fbaA;fructose-bisphosphatealdolase.classII[EC:4.1.2.13]	-6.482770493	1.53E-05
Metabolism;OverviewBiosynthesisofaminoacids[PATH:ko01230];GAPDH.gapA;glyceraldehyde3-phosphatedehydrogenase[EC:1.2.1.12]	-5.46010237	0.000134338
Metabolism;OverviewBiosynthesisofaminoacids[PATH:ko01230];gltB;glutamatesynthase(NADPH/NADH)largechain[EC:1.4.1.131.4.1.14]	-7.841446025	0.000132326
Metabolism;OverviewBiosynthesisofaminoacids[PATH:ko01230];gltD;glutamatesynthase(NADPH/NADH)smallchain[EC:1.4.1.131.4.1.14]	-12.19765424	0.00100271
Metabolism;OverviewBiosynthesisofaminoacids[PATH:ko01230];glyA.SHMT;glycinehydroxymethyltransferase[EC:2.1.2.1]	1.57364268	6.61E-07
Metabolism;OverviewBiosynthesisofaminoacids[PATH:ko01230];hisA;phosphoribosylformimino-5-aminoimidazolecarboxamideribotideisomerase[EC:5.3.1.16]	-1.487524168	0.046606821
Metabolism;OverviewBiosynthesisofaminoacids[PATH:ko01230];hisB;imidazoleglycerol-phosphatedehydratase[EC:4.2.1.19]	-2.975566053	0.000500234
Metabolism;OverviewBiosynthesisofaminoacids[PATH:ko01230];hisC;histidinol-phosphateaminotransferase[EC:2.6.1.9]	-4.325886491	1.99E-06
Metabolism;OverviewBiosynthesisofaminoacids[PATH:ko01230];hisD;histidinoldehydrogenase[EC:1.1.1.23]	-2.518577829	0.001771241
Metabolism;OverviewBiosynthesisofaminoacids[PATH:ko01230];hisE;phosphoribosyl-ATPpyrophosphohydrolase[EC:3.6.1.31]	-2.744707468	0.001947491
Metabolism;OverviewBiosynthesisofaminoacids[PATH:ko01230];hisF;cyclase[EC:4.1.3.-]	-2.060923175	0.010567335
Metabolism;OverviewBiosynthesisofaminoacids[PATH:ko01230];hisH;glutamineamidotransferase[EC:2.4.2.-]	-2.214467993	0.004442549
Metabolism;OverviewBiosynthesisofaminoacids[PATH:ko01230];hisI;phosphoribosyl-AMPcyclohydrolase[EC:3.5.4.19]	-2.184568523	0.008514636
Metabolism;OverviewBiosynthesisofaminoacids[PATH:ko01230];IDH1.IDH2.icd;isocitratedehydrogenase[EC:1.1.1.42]	-2.763414464	0.038521461
Metabolism;OverviewBiosynthesisofaminoacids[PATH:ko01230];leuB;3-isopropylmalatedehydrogenase[EC:1.1.1.85]	-1.446471659	0.024792564
Metabolism;OverviewBiosynthesisofaminoacids[PATH:ko01230];leuD;3-isopropylmalate/(R)-2-methylmalatedehydratasesmallsubunit[EC:4.2.1.334.2.1.35]	-2.749564252	0.000118783
Metabolism;OverviewBiosynthesisofaminoacids[PATH:ko01230];lysC;aspartatekinase[EC:2.7.2.4]	-2.470426967	0.000868497
Metabolism;OverviewBiosynthesisofaminoacids[PATH:ko01230];metC;cystathioninebeta-lyase[EC:4.4.1.8]	1.143510255	0.007708466
Metabolism;OverviewBiosynthesisofaminoacids[PATH:ko01230];OTC.argF.argI;ornithinecarbamoyltransferase[EC:2.1.3.3]	-2.288340744	0.002121995
Metabolism;OverviewBiosynthesisofaminoacids[PATH:ko01230];patA;aminotransferase[EC:2.6.1.-]	-3.048929303	3.84E-05
Metabolism;OverviewBiosynthesisofaminoacids[PATH:ko01230];serA.PHGDH;D-3-phosphoglyceratedehydrogenase[EC:1.1.1.95]	-3.36727392	5.99E-05
Metabolism;OverviewBiosynthesisofaminoacids[PATH:ko01230];serC.PSAT1;phosphoserineaminotransferase[EC:2.6.1.52]	-2.871256379	0.000521046
Metabolism;OverviewBiosynthesisofaminoacids[PATH:ko01230];thrA;bifunctionalaspartokinase/homoserinedehydrogenase1[EC:2.7.2.41.1.1.3]	-13.32943573	0.014525754
Metabolism;OverviewBiosynthesisofaminoacids[PATH:ko01230];thrB1;homoserinekinase[EC:2.7.1.39]	-1.925287818	0.00346328
Metabolism;OverviewBiosynthesisofaminoacids[PATH:ko01230];trpC;indole-3-glycerolphosphatesynthase[EC:4.1.1.48]	-4.542391636	0.000331713
Metabolism;OverviewBiosynthesisofaminoacids[PATH:ko01230];trpF;phosphoribosylanthranilateisomerase[EC:5.3.1.24]	0.705447491	0.034342378
Metabolism;OverviewCarbonmetabolism[PATH:ko01200];accB.bccP;acetyl-CoAcarboxylasebiotincarboxylcarrierprotein	0.846959361	0.049618655
Metabolism;OverviewCarbonmetabolism[PATH:ko01200];aceE;pyruvatedehydrogenaseE1component[EC:1.2.4.1]	-11.79245895	0.00766499
Metabolism;OverviewCarbonmetabolism[PATH:ko01200];arcC;carbamatekinase[EC:2.7.2.2]	-11.15287326	0.003472935
Metabolism;OverviewCarbonmetabolism[PATH:ko01200];DLD.lpd.pdhD;dihydrolipoamidedehydrogenase[EC:1.8.1.4]	-6.375720181	2.61E-05
Metabolism;OverviewCarbonmetabolism[PATH:ko01200];E2.2.1.2.talA.talB;transaldolase[EC:2.2.1.2]	-11.76644974	0.002995451
Metabolism;OverviewCarbonmetabolism[PATH:ko01200];E2.3.1.9.atoB;acetyl-CoAC-acetyltransferase[EC:2.3.1.9]	-3.373422428	0.00346328

Metabolism;OverviewCarbonmetabolism[PATH:ko01200];E2.7.1.12.gntK.idnK;gluconokinase[EC:2.7.1.12]	-5.702007645	4.30E-05
Metabolism;OverviewCarbonmetabolism[PATH:ko01200];E4.2.1.2B.fumC;fumaratehydratase.classII[EC:4.2.1.2]	-3.270061347	0.027939722
Metabolism;OverviewCarbonmetabolism[PATH:ko01200];ENO.eno;enolase[EC:4.2.1.11]	-5.951624563	6.12E-05
Metabolism;OverviewCarbonmetabolism[PATH:ko01200];fadJ;3-hydroxyacyl-CoAdehydrogenase/enoyl-CoAhydratase/3-hydroxybutyryl-CoAepimerase[EC:1.1.1.354.2.1.175.1.2.3]	-12.25582329	0.010739859
Metabolism;OverviewCarbonmetabolism[PATH:ko01200];FBA.fbaA;fructose-bisphosphatealdolase.classII[EC:4.1.2.13]	-6.482770493	1.53E-05
Metabolism;OverviewCarbonmetabolism[PATH:ko01200];FBP.fbp;fructose-1.6-bisphosphataseI[EC:3.1.3.11]	-11.79608401	0.008749896
Metabolism;OverviewCarbonmetabolism[PATH:ko01200];fdoG.fdfH;formatedehydrogenasemajorsubunit[EC:1.2.1.2]	-6.762554455	0.004407149
Metabolism;OverviewCarbonmetabolism[PATH:ko01200];folD;methylenetetrahydrofolatedehydrogenase(NADP+)/methenyltetrahydrofolatecyclohydrolase[EC:1.5.1.53.5.4.9]	1.161964243	1.48E-06
Metabolism;OverviewCarbonmetabolism[PATH:ko01200];frdA;fumaratereductaseflavoproteinsubunit[EC:1.3.5.4]	-3.39479139	0.012913123
Metabolism;OverviewCarbonmetabolism[PATH:ko01200];G6PD.zwf;glucose-6-phosphate1-dehydrogenase[EC:1.1.1.49]	-4.965135325	4.77E-07
Metabolism;OverviewCarbonmetabolism[PATH:ko01200];GAPDH.gapA;glyceraldehyde3-phosphatedehydrogenase[EC:1.2.1.12]	-5.46010237	0.000134338
Metabolism;OverviewCarbonmetabolism[PATH:ko01200];gcvT.AMT;aminomethyltransferase[EC:2.1.2.10]	-12.21984444	0.002190529
Metabolism;OverviewCarbonmetabolism[PATH:ko01200];GLDC.gcvP;glycinedehydrogenase[EC:1.4.4.2]	-8.292996831	0.024593612
Metabolism;OverviewCarbonmetabolism[PATH:ko01200];glyA.SHMT;glycinehydroxymethyltransferase[EC:2.1.2.1]	1.57364268	6.61E-07
Metabolism;OverviewCarbonmetabolism[PATH:ko01200];IDH1.IDH2.icd;isocitratedehydrogenase[EC:1.1.1.42]	-2.763414464	0.038521461
Metabolism;OverviewCarbonmetabolism[PATH:ko01200];kdgK;2-dehydro-3-deoxygluconokinase[EC:2.7.1.45]	-6.233354308	7.70E-08
Metabolism;OverviewCarbonmetabolism[PATH:ko01200];metF.MTHFR;methylenetetrahydrofolatereductase(NADPH)[EC:1.5.1.20]	-1.73540505	0.007781973
Metabolism;OverviewCarbonmetabolism[PATH:ko01200];PGD.gnd;6-phosphogluconatedehydrogenase[EC:1.1.1.44]	-4.864027831	2.76E-07
Metabolism;OverviewCarbonmetabolism[PATH:ko01200];pgl;6-phosphogluconolactonase[EC:3.1.1.31]	-6.034832737	6.61E-07
Metabolism;OverviewCarbonmetabolism[PATH:ko01200];ppdK;pyruvate.orthophosphatedikinase[EC:2.7.9.1]	-9.358232779	0.000641867
Metabolism;OverviewCarbonmetabolism[PATH:ko01200];pps.ppsA;pyruvate.waterdikinase[EC:2.7.9.2]	-3.962193451	0.029219419
Metabolism;OverviewCarbonmetabolism[PATH:ko01200];sdhB;succinatedehydrogenaseiron-sulfursubunit[EC:1.3.5.1]	-9.543035019	0.016387029
Metabolism;OverviewCarbonmetabolism[PATH:ko01200];serA.PHGDH;D-3-phosphoglyceratedehydrogenase[EC:1.1.1.95]	-3.36727392	5.99E-05
Metabolism;OverviewCarbonmetabolism[PATH:ko01200];serC.PSAT1;phosphoserineaminotransferase[EC:2.6.1.52]	-2.871256379	0.000521046
Metabolism;OverviewDegradationofaromaticcompounds[PATH:ko01220];adhE;acetaldehydedehydrogenase/alcoholdehydrogenase[EC:1.2.1.10 1.1.1.1]	-2.237328876	0.038521461
Metabolism;OverviewDegradationofaromaticcompounds[PATH:ko01220];adhP;alcoholdehydrogenase.propanol-preferring[EC:1.1.1.1]	-3.523029673	2.61E-05
Metabolism;OverviewDegradationofaromaticcompounds[PATH:ko01220];hcaD;3-phenylpropionate/trans-cinnamatedioxygenaseferredoxinreductasesubunit[EC:1.18.1.3]	-11.80808924	0.015307575
Metabolism;OverviewDegradationofaromaticcompounds[PATH:ko01220];pcaC;4-carboxymuconolactonedecarboxylase[EC:4.1.1.44]	-11.2802081	0.000379071
Metabolism;OverviewFattyacidmetabolism[PATH:ko01212];accB.bccP;acetyl-CoAcarboxylasebiotincarboxylcarrierprotein	0.846959361	0.049618655
Metabolism;OverviewFattyacidmetabolism[PATH:ko01212];E2.3.1.9.atoB;acetyl-CoAC-acetyltransferase[EC:2.3.1.9]	-3.373422428	0.00346328
Metabolism;OverviewFattyacidmetabolism[PATH:ko01212];fabI;enoyl-[acyl-carrierprotein]reductaseI[EC:1.3.1.91.3.1.10]	-4.200496577	0.026264115

Metabolism;OverviewFattyacidmetabolism[PATH:ko01212];fadJ;3-hydroxyacyl-CoAdehydrogenase/enoyl-CoAhydratase/3-hydroxybutyryl-CoAepimerase[EC:1.1.1.354.2.1.175.1.2.3]	-12.25582329	0.010739859
Metabolism;OverviewOxocarboxylicacidmetabolism[PATH:ko01210];argC;N-acetyl-gamma-glutamyl-phosphatereductase[EC:1.2.1.38]	-3.656685122	0.004196998
Metabolism;OverviewOxocarboxylicacidmetabolism[PATH:ko01210];argD;acetylornithine/N-succinyldiaminopimelateaminotransferase[EC:2.6.1.112.6.1.17]	-11.79756423	0.009303631
Metabolism;OverviewOxocarboxylicacidmetabolism[PATH:ko01210];E2.6.1.42.ilvE;branched-chainaminoacidaminotransferase[EC:2.6.1.42]	-2.752257097	0.000346456
Metabolism;OverviewOxocarboxylicacidmetabolism[PATH:ko01210];IDH1.IDH2.icd;isocitratedehydrogenase[EC:1.1.1.42]	-2.763414464	0.038521461
Metabolism;OverviewOxocarboxylicacidmetabolism[PATH:ko01210];leuB;3-isopropylmalatedehydrogenase[EC:1.1.1.85]	-1.446471659	0.024792564
Metabolism;OverviewOxocarboxylicacidmetabolism[PATH:ko01210];leuD;3-isopropylmalate/(R)-2-methylmalatedehydratasesmallsubunit[EC:4.2.1.334.2.1.35]	-2.749564252	0.000118783
Metabolism;OverviewOxocarboxylicacidmetabolism[PATH:ko01210];lysC;aspartatekinase[EC:2.7.2.4]	-2.470426967	0.000868497
Organismalsystem;Cellmobility;Bacterialchemotaxis[PATH:ko02030];cheR;chemotaxisproteinmethyltransferaseCheR[EC:2.1.1.80]	-13.11070865	0.003472935
Organismalsystem;Cellmobility;Bacterialchemotaxis[PATH:ko02030];cheV;two-componentsystem.chemotaxisfamily.responseregulatorCheV	-11.80327053	0.012048496
Organismalsystem;Cellmobility;Bacterialchemotaxis[PATH:ko02030];malE;maltose/maltodextrintransportsystemsubstrate-bindingprotein	-7.030153597	0.001616572
Organismalsystem;Cellmobility;Bacterialchemotaxis[PATH:ko02030];mcp;methyl-acceptingchemotaxisprotein	-4.13974575	0.043106084
Organismalsystem;Cellmobility;Bacterialchemotaxis[PATH:ko02030];rbsB;ribosetransportsystemsubstrate-bindingprotein	-6.526422376	1.42E-08
Organismalsystem;Cellmobility;Bacterialchemotaxis[PATH:ko02030];trg;methyl-acceptingchemotaxisproteinIII.riboseandgalactosesensorreceptor	-13.0990089	0.001572765

*Negative fold changes indicate genes up-regulated in cheese ripened at higher temperature.

Table S5. Description of the samples analysed in the two experiments carried out in this study.

**Sample ID**	**Description**	**Ripening conditions**	**RNA-seq***
First experiment			
**RM**	Raw Milk	NA	yes
**TM**	Thermized Milk	NA	yes
**NWC**	Natural Whey Culture	NA	yes
**Cu**	Curd before stretching	NA	yes
**Mol**	Cheese after molding	NA	yes
**t0.CO**	Cheese after brining and drying, before entering the ripening chamber - core	NA	yes
**t0.CR**	Cheese after brining and drying, before entering the ripening chamber - crust	NA	yes
**t10.CO**	Ripened cheese at 10 days - core	Standard	yes
**t10.CR**	Ripened cheese at 10 days - crust	Standard	yes
**t20.CO**	Ripened cheese at 20 days - core	Standard	yes
**t20.CR**	Ripened cheese at 20 days - crust	Standard	yes
**t30.CO**	Ripened cheese at 30 days - core	Standard	yes
**t30.CR**	Ripened cheese at 30 days - crust	Standard	yes
**t60.CO**	Ripened cheese at 60 days - core	Standard	yes
**t60.CR**	Ripened cheese at 60 days - crust	Standard	yes
Second experiment			
**RM**	Raw Milk	NA	yes
**TM**	Thermized Milk	NA	yes
**NWC**	Natural Whey Culture	NA	yes

**Cu**	Curd before stretching	NA	yes
**Mol**	Cheese after molding	NA	yes
**t0.CO**	Cheese after brining and drying, before entering the ripening chamber - core	NA	yes
**t0.CR**	Cheese after brining and drying, before entering the ripening chamber - crust	NA	yes
**t10.CO.A**	Ripened cheese at 10 days - core	Standard	yes
**t10.CO.B**	Ripened cheese at 10 days - core	Higher temperature	yes
**t10.CO.C**	Ripened cheese at 10 days - core	Lower RH	no
**t10.CR.A**	Ripened cheese at 10 days - crust	Standard	yes
**t10.CR.B**	Ripened cheese at 10 days - crust	Higher temperature	yes
**t10.CR.C**	Ripened cheese at 10 days - crust	Lower RH	no
**t20.CO.A**	Ripened cheese at 20 days - core	Standard	no
**t20.CO.B**	Ripened cheese at 20 days - core	Higher temperature	yes
**t20.CO.C**	Ripened cheese at 20 days - core	Lower RH	no
**t20.CR.A**	Ripened cheese at 20 days - crust	Standard	no
**t20.CR.B**	Ripened cheese at 20 days - crust	Higher temperature	yes
**t20.CR.C**	Ripened cheese at 20 days - crust	Lower RH	no
**t30.CO.A**	Ripened cheese at 30 days - core	Standard	yes
**t30.CO.B**	Ripened cheese at 30 days - core	Higher temperature	yes
**t30.CO.C**	Ripened cheese at 30 days - core	Lower RH	no
**t30.CR.A**	Ripened cheese at 30 days - crust	Standard	yes
**t30.CR.B**	Ripened cheese at 60 days - crust	Higher temperature	yes
**t30.CR.C**	Ripened cheese at 10 days - crust	Lower RH	no

NA: not applicable; Standard: ripening at 16°C and 75% RH. Higher temperature: ripening at 20°C and 75% RH. Lower RH: ripening at 16°C and 65% RH. *16S rRNA sequencing was carried out for all the samples of both the experiments.

Table S6. Bacterial genomes included in the reference database used in this study.

Genomes	NCBI genome ID
**downloaded from the NCBI RefSeq database**	
Enterobacter aerogenes KCTC 2190	NC_015663.1
Enterobacter cloacae EcWSU1	NC_016514.1
Enterobacter cloacae EcWSU1, plasmid pEcWSU1_A	NC_016515.1
Enterobacter cloacae SCF1	NC_014618.1
Enterobacter cloacae subsp. *cloacae* ATCC 13047	NC_014121.1
Enterobacter cloacae subsp. *cloacae* ATCC 13047, plasmid pECL_A	NC_014107.1
Enterobacter cloacae subsp. *cloacae* ATCC 13047, plasmid pECL_B	NC_014108.1
Enterococcus faecalis V583	NC_004668.1
Enterococcus faecalis V583, plasmid pTEF1	NC_004669.1
Enterococcus faecalis V583, plasmid pTEF2	NC_004671.1
Enterococcus faecalis V583, plasmid pTEF3	NC_004670.1
Erwinia amylovorans CFBP1430	NC_013961.1
Erwinia amylovorans CFBP1430, plasmid pEA29	NC_013957.1
Erwinia billingiae Eb661	NC_014306.1
Erwinia billingiae Eb661, plasmid pEB102	NC_014304.1
Erwinia billingiae Eb661, plasmid pEB170	NC_014305.1
Erwinia pyrifoliae Ep1/96	NC_012214.1
Erwinia pyrifoliae Ep1/96, plasmid pEP03	NC_013264.1
Erwinia pyrifoliae Ep1/96, plasmid pEP05	NC_013265.1
Erwinia pyrifoliae Ep1/96, plasmid pEP2.6	NC_013954.1
Erwinia pyrifoliae Ep1/96, plasmid pEP36	NC_013263.1
Erwinia tasmaniensis Et1/99	NC_010694.1
Erwinia tasmaniensis Et1/99, plasmid pET09	NC_010695.1
Erwinia tasmaniensis Et1/99, plasmid pET35	NC_010696.1
Erwinia tasmaniensis Et1/99, plasmid pET45	NC_010699.1
Erwinia tasmaniensis Et1/99, plasmid pET46	NC_010693.1
Erwinia tasmaniensis Et1/99, plasmid pET49	NC_010697.1
Escherichia coli 536	NC_008253.1
Escherichia coli ATCC 8739	NC_010468.1
Escherichia coli CF073	NC_004431.1
Escherichia coli K12 substr. DH10B	NC_010473.1
Escherichia coli K-12 substr. W3110	NC_007779.1

Escherichia coli O157:H7	NC_002655.2
Lactobacillus acidophilus 30SC	NC_015214.1
Lactobacillus acidophilus 30SC, plasmid pRKC30SC1	NC_015213.1
Lactobacillus acidophilus 30SC, plasmid pRKC30SC2	NC_015218.1
Lactobacillus acidophilus La-14	NC_021181.2
Lactobacillus acidophilus NCFM	NC_006814.3
Lactobacillus brevis ATCC367	NC_008497.1
Lactobacillus brevis ATCC367, plasmid 1	NC_008498.1
Lactobacillus brevis ATCC367, plasmid 2	NC_008499.1
Lactobacillus brevis KB290	NC_020819.1
Lactobacillus brevis KB290, plasmid pKB290-1	NC_020820.1
Lactobacillus brevis KB290, plasmid pKB290-2	NC_020821.1
Lactobacillus brevis KB290, plasmid pKB290-3	NC_020826.1
Lactobacillus brevis KB290, plasmid pKB290-4	NC_020822.1
Lactobacillus brevis KB290, plasmid pKB290-5	NC_020823.1
Lactobacillus brevis KB290, plasmid pKB290-6	NC_020827.1
Lactobacillus brevis KB290, plasmid pKB290-7	NC_020824.1
Lactobacillus brevis KB290, plasmid pKB290-8	NC_020828.1
Lactobacillus brevis KB290, plasmid pKB290-9	NC_020825.1
Lactobacillus buchneri CD034	NC_018610.1
Lactobacillus buchneri CD034, plasmid pCD034-1	NC_016035.1
Lactobacillus buchneri CD034, plasmid pCD034-2	NC_016034.1
Lactobacillus buchneri CD034, plasmid pCD034-3	NC_018611.1
Lactobacillus buchneri NRRL B-30929	NC_015428.1
Lactobacillus buchneri NRRL B-30929, plasmid pLBUC01	NC_015420.1
Lactobacillus buchneri NRRL B-30929, plasmid pLBUC02	NC_015429.1
Lactobacillus buchneri NRRL B-30929, plasmid pLBUC03	NC_015421.1
Lactobacillus casei ATCC334	NC_008526.1
Lactobacillus casei ATCC334, plasmid 1	NC_008502.1
Lactobacillus casei BD-II	NC_017474.1
Lactobacillus casei BD-II, plasmid pBD-II	NC_017476.1
Lactobacillus casei BL23	NC_010999.1
Lactobacillus casei LC2W	NC_017473.1
Lactobacillus casei LC2W, plasmid pLC2W	NC_017475.1
Lactobacillus casei LOCK919	NC_021721.1

Lactobacillus casei LOCK919, plasmid pLOCK919	NC_021722.1
Lactobacillus casei str. Zhang	NC_014334.1
Lactobacillus casei str. Zhang, plasmid plca36	NC_011352.1
Lactobacillus casei W56	NC_018641.1
Lactobacillus casei W56, plasmid pW56	NC_020057.1
Lactobacillus crispatus ST1	NC_014106.1
Lactobacillus delbrueckii subsp. *bulgaricus* 2038	NC_017469.1
Lactobacillus delbrueckii subsp. *bulgaricus* ATCC 11842	NC_008054.1
Lactobacillus delbrueckii subsp. *bulgaricus* ATCC BAA-365	NC_008529.1
Lactobacillus delbrueckii subsp. *bulgaricus* ND02	NC_014727.1
Lactobacillus delbrueckii subsp. *bulgaricus* ND02, plasmid unnamed	NC_014728.1
Lactobacillus fermentum CECT 5716	NC_017465.1
Lactobacillus fermentum F-6	NC_021235.1
Lactobacillus fermentum IFO 3956	NC_010610.1
Lactobacillus gasseri ATCC 33323	NC_008530.1
Lactobacillus helveticus CNRZ32	NC_021744.1
Lactobacillus helveticus DPC 4571	NC_010080.1
Lactobacillus helveticus H10	NC_017467.1
Lactobacillus helveticus H10, plasmid pH10	NC_017468.1
Lactobacillus helveticus R0052	NC_018528.1
Lactobacillus johnsonii FI9785	NC_012552.1
Lactobacillus johnsonii DPC 6026	NC_017477.1
Lactobacillus johnsonii FI9785	NC_013504.1
Lactobacillus johnsonii FI9786, plasmid p9785L	NC_013505.1
Lactobacillus johnsonii N6.2	NC_022909.1
Lactobacillus johnsonii NCC533	NC_005362.1
Lactobacillus kefiranofaciens ZW3	NC_015602.1
Lactobacillus kefiranofaciens ZW3, plasmid pWW1	NC_015598.1
Lactobacillus kefiranofaciens ZW3, plasmid pWW2	NC_015603.1
Lactobacillus paracasei subsp. *paracasei* 8700:2	NC_022112.1
Lactobacillus paracasei subsp. *paracasei* 8700:2, plasmid 1	NC_022114.1
Lactobacillus paracasei subsp. *paracasei* 8700:2, plasmid 2	NC_022123.1
Lactobacillus plantarum 16	NC_021514.1
Lactobacillus plantarum 16, plasmid Lp16A	NC_021515.1
Lactobacillus plantarum 16, plasmid Lp16B	NC_021525.1

Lactobacillus plantarum 16, plasmid Lp16C	NC_021516.1
Lactobacillus plantarum 16, plasmid Lp16D	NC_021526.1
Lactobacillus plantarum 16, plasmid Lp16E	NC_021517.1
Lactobacillus plantarum 16, plasmid Lp16F	NC_021518.1
Lactobacillus plantarum 16, plasmid Lp16G	NC_021527.1
Lactobacillus plantarum 16, plasmid Lp16H	NC_021519.1
Lactobacillus plantarum 16, plasmid Lp16I	NC_021528.1
Lactobacillus plantarum 16, plasmid Lp16L	NC_021520.1
Lactobacillus plantarum JDM1	NC_012984.1
Lactobacillus plantarum subsp. *plantarum* P-8	NC_021224.1
Lactobacillus plantarum subsp. *plantarum* P-8, plasmid LBPp1	NC_021233.1
Lactobacillus plantarum subsp. *plantarum* P-8, plasmid LBPp2	NC_021225.1
Lactobacillus plantarum subsp. *plantarum* P-8, plasmid LBPp3	NC_021226.1
Lactobacillus plantarum subsp. *plantarum* P-8, plasmid LBPp4	NC_021234.1
Lactobacillus plantarum subsp. *plantarum* P-8, plasmid LBPp5	NC_021227.1
Lactobacillus plantarum subsp. *plantarum* P-8, plasmid LBPp6	NC_021228.1
Lactobacillus plantarum subsp. *plantarum* ST-III	NC_014554.1
Lactobacillus plantarum subsp. *plantarum* ST-III, plasmid pST-III	NC_014558.2
Lactobacillus plantarum WCFS1	NC_004567.2
Lactobacillus plantarum WCFS1, plasmid pWCFS101	NC_006375.1
Lactobacillus plantarum WCFS1, plasmid pWCFS102	NC_006376.1
Lactobacillus plantarum WCFS1, plasmid pWCFS103	NC_006377.1
Lactobacillus plantarum ZJ316	NC_020229.1
Lactobacillus plantarum ZJ316, plasmid pLP-ZJ101	NC_021903.1
Lactobacillus plantarum ZJ316, plasmid pLP-ZJ102	NC_021904.1
Lactobacillus plantarum ZJ316, plasmid pLP-ZJ103	NC_021912.1
Lactobacillus reuteri DSM20016	NC_009513.1
Lactobacillus reuteri I5007	NC_021494.1
Lactobacillus reuteri I5007, plasmid pLRI01	NC_021503.1
Lactobacillus reuteri I5007, plasmid pLRI02	NC_021496.1
Lactobacillus reuteri I5007, plasmid pLRI03	NC_021495.1
Lactobacillus reuteri I5007, plasmid pLRI04	NC_021504.1
Lactobacillus reuteri I5007, plasmid pLRI05	NC_021497.1
Lactobacillus reuteri I5007, plasmid pLRI06	NC_021498.1
Lactobacillus reuteri JCM 1112	NC_010609.1

Lactobacillus reuteri SD2112	NC_015697.1
Lactobacillus reuteri SD2112, plasmid pLR580	NC_015699.1
Lactobacillus reuteri SD2112, plasmid pLR581	NC_015700.1
Lactobacillus reuteri SD2112, plasmid pLR584	NC_015701.1
Lactobacillus reuteri SD2112, plasmid pLR585	NC_015698.1
Lactobacillus reuteri TD1	NC_021872.1
Lactobacillus rhamnosus ATCC 8530	NC_017491.1
Lactobacillus rhamnosus GG ATCC 53103	NC_017482.1
Lactobacillus rhamnosus Lc 705	NC_013199.1
Lactobacillus rhamnosus Lc 705, plasmid pLC1	NC_013200.1
Lactobacillus rhamnosus LOCK900	NC_021723.1
Lactobacillus rhamnosus LOCK908	NC_021725.1
Lactobacillus ruminis ATCC 27782	NC_015975.1
Lactobacillus sakei subsp. *sakei* 23K	NC_007576.1
Lactobacillus salivarius CECT 5713	NC_017481.1
Lactobacillus salivarius CECT 5713, plasmid pHN1	NC_017479.1
Lactobacillus salivarius CECT 5713, plasmid pHN2	NC_017480.1
Lactobacillus salivarius CECT 5713, plasmid pHN3	NC_017499.1
Lactobacillus salivarius UCC118	NC_007929.1
Lactobacillus salivarius UCC118, plasmid pMP118	NC_007930.1
Lactobacillus salivarius UCC118, plasmid pSF118-20	NC_006529.1
Lactobacillus salivarius UCC118, plasmid pSF118-44	NC_006530.1
Lactobacillus sanfranciscensis TMW 11.304	NC_015978.1
Lactobacillus sanfranciscensis TMW 11.304, plasmid pLS1	NC_015979.1
Lactobacillus sanfranciscensis TMW 11.304, plasmid pLS2	NC_015980.1
Lactococcus garviae ATCC 49156	NC_015930.1
Lactococcus garviae Lg2	NC_017490.1
Lactococcus lactis subsp. *cremoris* A76	NC_017492.1
Lactococcus lactis subsp. *cremoris* A76, plasmid pQA504	NC_017497.1
Lactococcus lactis subsp. *cremoris* A76, plasmid pQA518	NC_017495.1
Lactococcus lactis subsp. *cremoris* A76, plasmid pQA549	NC_017493.1
Lactococcus lactis subsp. *cremoris* A76, plasmid pQA554	NC_017496.1
Lactococcus lactis subsp. *cremoris* KW2	NC_022369.1
Lactococcus lactis subsp. *cremoris* MG1363	NC_009004.1
Lactococcus lactis subsp. *cremoris* NZ9000	NC_017949.1

Lactococcus lactis subsp. *cremoris* plasmid 1	NC_008503.1
Lactococcus lactis subsp. *cremoris* plasmid 2	NC_008504.1
Lactococcus lactis subsp. *cremoris* plasmid 3	NC_008505.1
Lactococcus lactis subsp. *cremoris* plasmid 4	NC_008506.1
Lactococcus lactis subsp. *cremoris* plasmid 5	NC_008507.1
Lactococcus lactis subsp. *cremoris* SK11	NC_008527.1
Lactococcus lactis subsp. *cremoris* UC509.9	NC_019435.1
Lactococcus lactis subsp. *cremoris* UC509.9, plasmid pCIS1	NC_019438.1
Lactococcus lactis subsp. *cremoris* UC509.9, plasmid pCIS2	NC_019434.1
Lactococcus lactis subsp. *cremoris* UC509.9, plasmid pCIS3	NC_019433.1
Lactococcus lactis subsp. cremoris UC509.9, plasmid pCIS4	NC_019437.1
Lactococcus lactis subsp. cremoris UC509.9, plasmid pCIS5	NC_019432.1
Lactococcus lactis subsp. *cremoris* UC509.9, plasmid pCIS6	NC_019436.1
Lactococcus lactis subsp. *cremoris* UC509.9, plasmid pCIS7	NC_019431.1
Lactococcus lactis subsp. *cremoris* UC509.9, plasmid pCIS8	NC_019430.1
Lactococcus lactis subsp. *lactis* CV56	NC_017486.1
Lactococcus lactis subsp. *lactis* CV56, plasmid pCV56A	NC_017483.1
Lactococcus lactis subsp. *lactis* CV56, plasmid pCV56B	NC_017487.1
Lactococcus lactis subsp. *lactis* CV56, plasmid pCV56C	NC_017484.1
Lactococcus lactis subsp. *lactis* CV56, plasmid pCV56D	NC_017485.1
Lactococcus lactis subsp. *lactis* CV56, plasmid pCV56E	NC_017488.1
Lactococcus lactis subsp. *lactis* Il1403	NC_002662.1
Lactococcus lactis subsp. *lactis* IO-1	NC_020450.1
Lactococcus lactis subsp. *lactis* KF147	NC_013656.1
Lactococcus lactis subsp. *lactis* KF148, plasmid pKF147A	NC_013657.1
Lactococcus lactis subsp. *lactis* KLDS 4.0325	NC_022593.1
Leuconostoc carnosum JB16	NC_018673.1
Leuconostoc carnosum JB16, plasmid pKLC1	NC_018674.1
Leuconostoc carnosum JB16, plasmid pKLC2	NC_018698.1
Leuconostoc carnosum JB16, plasmid pKLC3	NC_018675.1
Leuconostoc carnosum JB16, plasmid pKLC4	NC_018699.1
Leuconostoc citreum KM20	NC_010471.1
Leuconostoc citreum KM20, plasmid pLCK1	NC_010470.1
Leuconostoc citreum KM20, plasmid pLCK2	NC_010466.1
Leuconostoc citreum KM20, plasmid pLCK3	NC_010467.1

Leuconostoc citreum KM20, plasmid pLCK4	NC_010469.1
Leuconostoc gasicomitatum LMG 18811	NC_014319.1
Leuconostoc gelidum JB7	NC_018631.1
Leuconostoc kimkii IMSNU 11154	NC_014136.1
Leuconostoc kimkii IMSNU 11154, plasmid LkipL4701	NC_014131.1
Leuconostoc kimkii IMSNU 11154, plasmid LkipL4704	NC_014132.1
Leuconostoc kimkii IMSNU 11154, plasmid LkipL4719	NC_014133.1
Leuconostoc kimkii IMSNU 11154, plasmid LkipL4726	NC_014134.1
Leuconostoc kimkii IMSNU 11154, plasmid LkipL48	NC_014135.1
Leuconostoc mesenteroides subsp. *mesenteroides* ATCC8293	NC_008531.1
Leuconostoc mesenteroides subsp. *mesenteroides* ATCC8293, plasmid pLEUM 1	NC_008496.1
Leuconostoc mesenteroides subsp. *mesenteroides* J18	NC_016805.1
Leuconostoc mesenteroides subsp. *mesenteroides* J18, plasmid pKLE01	NC_016827.1
Leuconostoc mesenteroides subsp. *mesenteroides* J18, plasmid pKLE02	NC_016820.1
Leuconostoc mesenteroides subsp. *mesenteroides* J18, plasmid pKLE03	NC_016821.1
Leuconostoc mesenteroides subsp. *mesenteroides* J18, plasmid pKLE04	NC_016828.1
Leuconostoc mesenteroides subsp. *mesenteroides* J18, plasmid pKLE05	NC_016806.1
Leuconostoc sp. C2	NC_015734.1
Pantoea ananatis LMG 20103	NC_013956.2
Pantoea sp. At-9b	NC_014837.1
Pantoea sp. At-9b, plasmid pPAT9B01	NC_014838.1
Pantoea sp. At-9b, plasmid pPAT9B02	NC_014839.1
Pantoea sp. At-9b, plasmid pPAT9B03	NC_014840.1
Pantoea sp. At-9b, plasmid pPAT9B04	NC_014841.1
Pantoea sp. At-9b, plasmid pPAT9B05	NC_014842.1
Pediococcus claussenii ATCC BAA-344	NC_016605.1
Pediococcus claussenii ATCC BAA-344, plasmid pPECL-1	NC_016635.1
Pediococcus claussenii ATCC BAA-344, plasmid pPECL-2	NC_016606.1
Pediococcus claussenii ATCC BAA-344, plasmid pPECL-3	NC_016636.1
Pediococcus claussenii ATCC BAA-344, plasmid pPECL-4	NC_016607.1
Pediococcus claussenii ATCC BAA-344, plasmid pPECL-5	NC_016608.1
Pediococcus claussenii ATCC BAA-344, plasmid pPECL-6	NC_017017.1
Pediococcus claussenii ATCC BAA-344, plasmid pPECL-7	NC_017018.1
Pediococcus claussenii ATCC BAA-344, plasmid pPECL-8	NC_017019.1
Pediococcus pentosaceus ATCC 25745	NC_008525.1

Pediococcus pentosaceus SL4	NC_022780.1
Propionibacterium acnes KPA17120	NC_006085.1
Propionibacterium freudenreichii subsp. *shermanii* CIRM-BIA1	NC_014215.1
Pseudomonas putida GB-1	NC_010322.1
Pseudomonas putida KT2440	NC_002947.3
Psychrobacter arcticus 273-4	NC_007204.1
Psychrobacter cryohalolentis K5	NC_007969.1
Psychrobacter cryohalolentis K5, plasmid 1	NC_007968.1
Psychrobacter sp. PRwf-1	NC_009524.1
Psychrobacter sp. PRwf-1, plasmid pRWF101	NC_009516.1
Psychrobacter sp. PRwf-1, plasmid pRWF102	NC_009517.1
Rahnella aquatilis ATCC 33071	NC_016818.1
Rahnella aquatilis ATCC 33071, plasmid	NC_016819.1
Rahnella aquatilis ATCC 33071, plasmid	NC_016835.1
Rahnella aquatilis ATCC 33071, plasmid	NC_017092.1
Ralstonia solanacearum Po82	NC_017574.1
Ralstonia solanacearum Po82, megaplasmid	NC_017575.1
Serratia proteamaculans 568	NC_009832.1
Serratia proteamaculans 568, plasmid pSPRO01	NC_009829.1
Shewanella denitrificans OS217	NC_007954.1
Shewanella frigidimarina NCIMB 400	NC_008345.1
Shewanella oneidensis MR-1	NC_004347.2
Shewanella oneidensis MR-1, plasmid	NC_004349.1
Shewanella piezotolerans WP3	NC_011566.1
Shewanella putrefaciens CN-32	NC_009438.1
Shewanella sp. ANA-3	NC_008577.1
Shewanella sp. ANA-3, plasmid 1	NC_008573.1
Shigella flexneri 2°	NC_004337.2
Shigella flexneri 2a, plasmid pCP301	NC_004851.1
Staphylococcus haemolyticus JCSC1435	NC_007168.1
Staphylococcus haemolyticus JCSC1435, plasmid pSHaeA	NC_007169.1
Staphylococcus haemolyticus JCSC1435, plasmid pSHaeB	NC_007170.1
Staphylococcus haemolyticus JCSC1435, plasmid pSHaeC	NC_007171.1
Staphylococcus pseudointermedius ED99	NC_017568.1
Staphylococcus aureus subsp. *aureus* NCTC 8325	NC_007795.1

Staphylococcus carnosus subsp. *carnosus* TM300	NC_012121.1
Streptococcus agalactiae NEM316	NC_004368.1
Streptococcus dysgalactiae subsp. *equisimilis* ATCC 12394	NC_017567.1
Streptococcus macedonicus ACA-DC 198	NC_016749.1
Streptococcus macedonicus ACA-DC 198, plasmid pSMA198	NC_016750.1
Streptococcus mitis B6	NC_013853.1
Streptococcus oralis Uo5	NC_015291.1
Streptococcus pneumoniae R6	NC_003098.1
Streptococcus pneumoniae TIGR4	NC_003028.3
Streptococcus pyogenes SF370	NC_002737.1
Streptococcus salivarius 57.I	NC_017594.1
Streptococcus suis 05ZYH33	NC_009442.1
Streptococcus thermophilus CNRZ1066	NC_006449.1
Streptococcus thermophilus JIM 8232	NC_017581.1
Streptococcus thermophilus LMD-9	NC_008532.1
Streptococcus thermophilus LMD-9, plasmid 1	NC_008500.1
Streptococcus thermophilus LMD-9, plasmid 2	NC_008501.1
Streptococcus thermophilus LMG18311	NC_006448.1
Streptococcus thermophilus MN-ZLW-002	NC_017927.1
Streptococcus thermophilus ND03	NC_017563.1
Streptococcus uberis 0140J	NC_012004.1
**downloaded from http://patricbrc.org/portal/**	
Enterococcus casseliflavus EC20	NA
Enterococcus gallinarum EG2	NA
Lactobacillus reuteri 100-23	NA
Pediococcus acidilactici DSM20284	NA
